# Engineered ACE2 receptor traps potently neutralize SARS-CoV-2

**DOI:** 10.1073/pnas.2016093117

**Published:** 2020-10-22

**Authors:** Anum Glasgow, Jeff Glasgow, Daniel Limonta, Paige Solomon, Irene Lui, Yang Zhang, Matthew A. Nix, Nicholas J. Rettko, Shoshana Zha, Rachel Yamin, Kevin Kao, Oren S. Rosenberg, Jeffrey V. Ravetch, Arun P. Wiita, Kevin K. Leung, Shion A. Lim, Xin X. Zhou, Tom C. Hobman, Tanja Kortemme, James A. Wells

**Affiliations:** ^a^Department of Bioengineering and Therapeutic Sciences, University of California, San Francisco, CA 94158;; ^b^Department of Pharmaceutical Chemistry, University of California, San Francisco, CA 94158;; ^c^Li Ka Shing Institute of Virology, University of Alberta, Edmonton, AB T6G 2E1, Canada;; ^d^Department of Cell Biology, University of Alberta, Edmonton, AB T6G 2H7, Canada;; ^e^Department of Laboratory Medicine, University of California, San Francisco, CA 94143;; ^f^Department of Medicine, University of California, San Francisco, CA 94143;; ^g^Laboratory of Molecular Genetics and Immunology, Rockefeller University, New York, NY 10065;; ^h^Department of Medical Microbiology and Immunology, University of Alberta, Edmonton, AB T6G 2R3, Canada;; ^i^Department of Cellular and Molecular Pharmacology, University of California, San Francisco, CA 94158

**Keywords:** SARS-CoV-2, antiviral therapeutics, computational design, yeast display, receptor trap

## Abstract

An essential mechanism for severe acute respiratory syndrome coronavirus 1 (SARS-CoV-1) and severe acute respiratory syndrome coronavirus 2 (SARS-CoV-2) infection begins with the viral spike protein binding to the human receptor protein angiotensin-converting enzyme II (ACE2). Here, we describe a stepwise engineering approach to generate a set of affinity optimized, enzymatically inactivated ACE2 variants that potently block SARS-CoV-2 infection of cells. These optimized receptor traps tightly bind the receptor binding domain (RBD) of the viral spike protein and prevent entry into host cells. We first computationally designed the ACE2–RBD interface using a two-stage flexible protein backbone design process that improved affinity for the RBD by up to 12-fold. These designed receptor variants were affinity matured an additional 14-fold by random mutagenesis and selection using yeast surface display. The highest-affinity variant contained seven amino acid changes and bound to the RBD 170-fold more tightly than wild-type ACE2. With the addition of the natural ACE2 collectrin domain and fusion to a human immunoglobulin crystallizable fragment (Fc) domain for increased stabilization and avidity, the most optimal ACE2 receptor traps neutralized SARS-CoV-2–pseudotyped lentivirus and authentic SARS-CoV-2 virus with half-maximal inhibitory concentrations (IC50s) in the 10- to 100-ng/mL range. Engineered ACE2 receptor traps offer a promising route to fighting infections by SARS-CoV-2 and other ACE2-using coronaviruses, with the key advantage that viral resistance would also likely impair viral entry. Moreover, such traps can be predesigned for viruses with known entry receptors for faster therapeutic response without the need for neutralizing antibodies isolated from convalescent patients.

## Significance

During the ongoing COVID-19 pandemic, protein engineering offers a rapid and powerful approach for building therapeutics to treat severe acute respiratory syndrome coronavirus 2 (SARS-CoV-2) infections. We use computational design, affinity maturation, and fusion to dimerization domains to engineer “receptor traps” based on wild-type angiotensin-converting enzyme II (ACE2), the target for viral spike-mediated SARS-CoV-2 entry into cells. The optimized ACE2 receptor traps neutralize authentic SARS-CoV-2 infections as effectively as high-affinity antibodies isolated from convalescent patients and also bind viral spike proteins from other coronaviruses known to cause respiratory diseases. ACE2 receptor traps have large binding interfaces and block the entire receptor binding interface, limiting the potential impact of viral escape mutations.

There is an urgent need for broadly effective therapeutics to treat severe acute respiratory syndrome coronavirus 2 (SARS-CoV-2) infections during the ongoing COVID-19 pandemic (1, 2). Antibodies isolated from convalescent patient sera and recombinant antibodies cloned from the B cells of recovered patients have been effective in past and recent pandemics, and much of the ongoing drug development effort is based on these approaches (3–8). However, strategies for antibody development necessarily follow widespread viral spread and infection, which costs precious time in a rapidly developing pandemic.

Protein engineering approaches to identify binders to viral entry proteins offer a rapid alternative, without the prerequisite for an infected population. In the first step of a severe acute respiratory syndrome coronavirus 1 (SARS-CoV-1) or SARS-CoV-2 infection, the receptor binding domain (RBD) of the trimeric spike protein on the surface of the virus binds to the membrane-bound receptor angiotensin-converting enzyme II (ACE2) to enter human cells (3, 4, 8). Most neutralizing antibodies to SARS-CoV-1 and SARS-CoV-2 block viral entry by binding to the ACE2 binding site on the RBD. Ongoing efforts by our laboratory and others use in vitro methods, such as phage display or yeast display, from naïve libraries to generate recombinant antibodies or other formatted domains to block viral entry (9, 10).

As an alternate strategy, we pursued development of ACE2 “receptor traps”: affinity-optimized soluble variants of the ACE2 extracellular domain that block the viral spike protein from binding cellular ACE2 and facilitating entry (11). This approach has the potential advantage that viral resistance to an ACE2 receptor trap would also inhibit the ability of the virus to enter via binding to the ACE2 entry receptor. Receptor traps would also be useful for both pandemic SARS-CoV-1 and SARS-CoV-2 as well as other emerging variant strains that use ACE2 as a common entry port. Furthermore, the soluble extracellular domain of wild-type (WT) human recombinant ACE2 (APN01) was found to be safe in healthy volunteers (12) and in a small cohort of patients with acute respiratory distress syndrome (13) by virtue of ACE2’s intrinsic angiotensin-converting activity, which is not required for viral entry. APN01 is currently in phase 2 clinical trials in Europe for treatment of SARS-CoV-2 (14) (NCT04335136). However, we and others have shown that WT ACE2 binds the SARS-CoV-2 spike RBD with only modest affinity ($K_{D}$ ∼ 15 nM) (15–17). ACE2 is therefore a good candidate for affinity optimization, especially because potent blocking antibodies to the spike protein can be isolated with binding affinity ($K_{D}$) values in the mid- to low-picomolar range (3, 4, 6, 8, 9, 18–20).

Here, we improve the binding affinity of ACE2 for the monomeric spike RBD by 170-fold using a hybrid computational and experimental protein engineering approach. We demonstrate that after fusion to a human immunoglobulin (IgG) crystallizable fragment (Fc) domain and the natural collectrin domain of ACE2, our most effective ACE2-Fc variant has a half-maximal inhibitory concentration (IC50) of 28 ng/mL in pseudotyped SARS-CoV-2 neutralization assays and comparable neutralization in authentic SARS-CoV-2 infection assays, reducing viral replication to almost undetectable levels. ACE2 receptor traps are promising therapeutic candidates, especially given the potential for viral escape mutations to impact antibody efficacy (5, 21) and low neutralizing antibody levels in a subset of recovered patients (6).

## Results

**Fig. 1.**
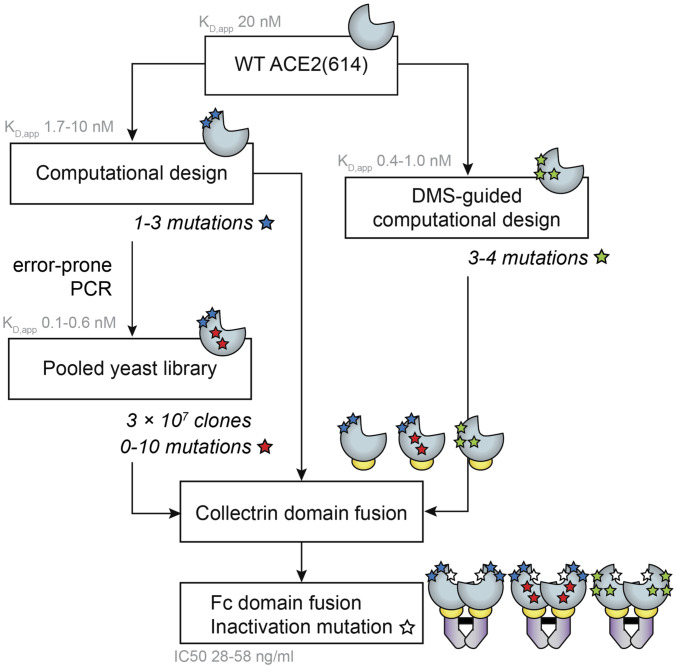
ACE2 receptor trap strategy. Two computational design strategies were used to predict mutations to ACE2(614) (light blue shape) that enhance its affinity for the SARS-CoV-2 spike RBD: 1) saturation mutagenesis at computational alanine scanning hot spots, followed by local ACE2 redesign (blue star mutations) and 2) combining mutations from computational saturation mutagenesis and experimental DMS data (green star mutations). Designed ACE2 variants were mutagenized and screened for binding to the RBD, and additional mutations that improved the binding affinity were isolated (red star mutations). Engineered ACE2(614) variants were fused to the ACE2 collectrin domain (residues 615 to 740, yellow ovals) and expressed as Fc fusions (purple shapes) with an additional mutation to inactivate ACE2 peptidase activity (white star mutations). $K_{D,app}$ values represent apparent binding affinities measured between yeast surface-displayed ACE2(614) variants and the monomeric RBD. IC50 values represent those measured for the four most potent neutralizing variants in SARS-CoV-2 pseudotyped virus assays.

We reengineered the soluble extracellular domain of ACE2 [residues 18 to 614, ACE2(614)] to bind the RBD of the SARS-CoV-2 spike protein using a combined computational/experimental protein engineering strategy (Fig. 1). First, we computationally redesigned ACE2(614) using the Rosetta macromolecular modeling suite, introducing sets of mutations that improved the $K_{D}$ of an ACE2(614)-Fc fusion protein for the SARS-CoV-2 spike RBD from 3- to 11-fold over the WT ACE2(614)-Fc protein in biolayer interferometry (BLI) binding assays. Then, we affinity matured the improved ACE2(614) designs in a pooled yeast display format. Additional mutations discovered through yeast display conferred a further 14-fold improvement in the apparent binding affinity ($K_{D,app}$) for the RBD over the computationally designed parent ACE2(614), as measured on the surface of yeast. The final ACE2 variants have $K_{D,app}$ close to 100 pM for the monomeric spike RBD.

**Fig. 2.**
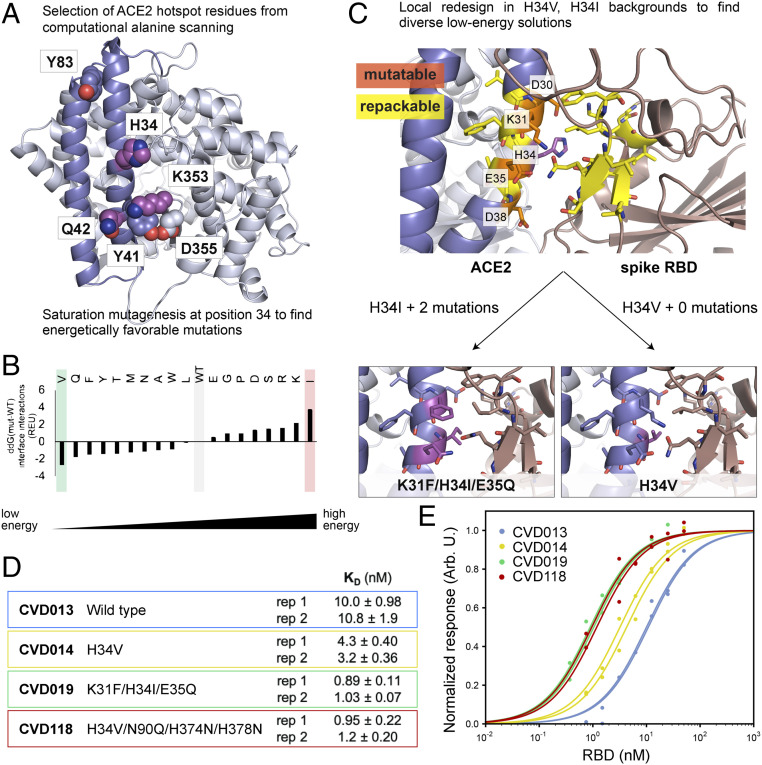
Computational design of ACE2 for improved binding affinity to the spike RBD. (*A*) Computational alanine scanning identified ACE2 hot spot residues (shown as spheres) that contribute strongly to binding the spike RBD. Residues 18 to 90 are shown in blue, and residues 91 to 614 are shown in light blue. H34, Q42, and K353, shown as magenta spheres, were selected for computational saturation mutagenesis. (*B*) Computational saturation mutagenesis predicted several stabilizing mutations to H34. H34V and H34I were selected for further design to generate diverse ACE2 receptor trap models. (*C*) Flexible backbone design was performed around V34 ACE2 and I34 ACE2 (WT residue H34 shown in magenta). ACE2 residues that were permitted to change amino acid identity (“mutable” residues) are labeled and shown in orange; ACE2 and RBD residues that were allowed to change rotameric conformations and/or backbone atom positions (“repackable” residues) are shown in yellow. Design around H34I resulted in two additional mutations, shown in magenta. (*D* and *E*) In vitro BLI measurements show that designed ACE2(614)-Fc binding affinities to the RBD are improved 2- to 11-fold over WT ACE2(614)-Fc. To generate CVD118, the N90Q glycan knockout mutation from DMS (24) was added to V34 ACE2, as well as two histidine mutations to inactivate ACE2 peptidase activity (25). Each “rep” is a separate biological replicate. The table in *D* lists the $K_{D}$ values for the designed ACE2 variants, with errors of the fits for titration curves shown in *E*.

High-resolution ACE2–RBD structures (22, 23) show a large, flat binding interface primarily comprising the N-terminal helices of ACE2 (residues 18 to 90), with secondary interaction sites spanning residues 324 to 361 (Fig. 2*A*). To computationally redesign ACE2(614) for increased binding affinity with the RBD, we first determined which amino acid side chains are most crucial to the ACE2–RBD interaction (“hot spots”) by performing a computational alanine scan on the binding interface using an established method in Rosetta (26, 27). Then, we systematically redesigned a subset of hot spot residues and their local environment to generate models for interfaces and selected the lowest (best)-scoring ACE2 designs for testing (Fig. 2 *A*–*C*).

Computational alanine scanning suggested that the binding affinity of the ACE2–RBD interaction depends most crucially on six amino acid side chains (H34, Y41, Q42, Y83, K353, and D355) on ACE2 as assessed by values of the predicted change in binding energy upon mutation to alanine [DDG(complex)] greater than 1 Rosetta Energy Unit (REU) (Fig. 2*A* and *SI Appendix*, Table S1). To determine which of these hot spots to target for computational design, we evaluated each hot spot residue by two metrics: the per-residue energy and the contribution of the residue to the interface energy (*Materials and Methods*). Higher energies indicate lower stability. Hot spot residues H34, Q42, and K353 were targeted for further design.

To determine whether point mutations at these positions could improve the ACE2–RBD binding affinity, we performed computational saturation mutagenesis at these positions (excluding mutations to cysteine to avoid potential disulfide bond formation) and recalculated the interface energy for each model (*SI Appendix*, Table S2). While we found no amino acid substitutions at positions 42 and 353 that were predicted to be stabilizing, several substitutions at position 34 were predicted to improve the interaction energy between ACE2 and the RBD (Fig. 2*B*). Histidine 34 was mutated to a valine in the lowest-energy model because we anticipated favorable hydrophobic interactions with leucine 455 in the RBD. In the highest-energy model, histidine 34 was mutated to an overly bulky isoleucine.

We reasoned that both H34V and H34I, as the “best” and “worst” point mutants, were predicted to substantially affect the interface energy in the context of their chemical environment and that additional local mutations might improve binding affinity in both models to yield different viable solutions. We applied the Rosetta “Coupled Moves” flexible backbone design protocol (28) to redesign the local environment of V34 and I34 in each model (Fig. 2*C*). ACE2 side chains within 4 Å of residue 34 were allowed to mutate, while other ACE2 and RBD side chains within 8 Å of ACE2 residue 34 could change rotamer and/or backbone conformations (“repacking”) (*Materials and Methods*). This approach did not identify additional favorable mutations to H34V ACE2 in any of the models, but there were one to four additional mutations in the H34I ACE2 models. The lowest energy-designed protein based on H34I ACE2 had two additional mutations: K31F and E35Q (Fig. 2*C* and *SI Appendix*, Fig. S1). In this solution, ACE2 Q35 made a hydrogen bond with a repositioned RBD Q493, and ACE2 I34 packed against a repositioned RBD L455. On the ACE2 side of the interface, F31 made a favorable hydrophobic interaction with the methylene in Q35. For both lowest-energy redesigned interfaces, the rmsds of the mutable and repackable positions (atoms corresponding to ACE2 residues 29 to 39 and RBD residues 416 to 418, 452 to 456, and 492 to 494) in the model vs. the WT structure were less than 1 Å, and the total summed predicted pairwise interface energies for both design solutions were comparable (*SI Appendix*, Fig. S1).

Next, we characterized the binding affinities of the computationally designed ACE2 variants as Fc fusions for spike RBD in BLI assays using purified proteins. We transiently expressed ACE2(614) with a C-terminal human IgG Fc domain fusion for improved affinity to spike, as shown in previous studies (11, 29), in Expi293 cells (*Materials and Methods*). The BLI-measured $K_{D}$ values of computationally designed H34V ACE2(614)-Fc and K31F/H34I/E35Q ACE2(614)-Fc for the RBD were measured to be 3 and 11 times lower than the WT ACE2(614)-Fc, respectively (Fig. 2 *D* and *E*). We also tested binding of the RBD to H34V/N90Q/H374N/H378N ACE2(614)-Fc to determine the impact of removing a glycan that is adjacent to the interface at N90, as well as the protein’s native peptidase activity. A recent deep mutational scanning (DMS) study reported enrichment for ACE2 variants with mutations at the N90 glycosylation site (24), and histidines in positions 374 and 378 together coordinate a ${Zn}^{2+}$ ion necessary for enzymatic activity (*SI Appendix*, Fig. S2) (11). In the BLI assay, H34V/N90Q/H374N/H378N ACE2(614)-Fc showed 10-fold improved affinity over WT ACE2(614)-Fc (Fig. 2 *D* and *E*).

**Fig. 3.**
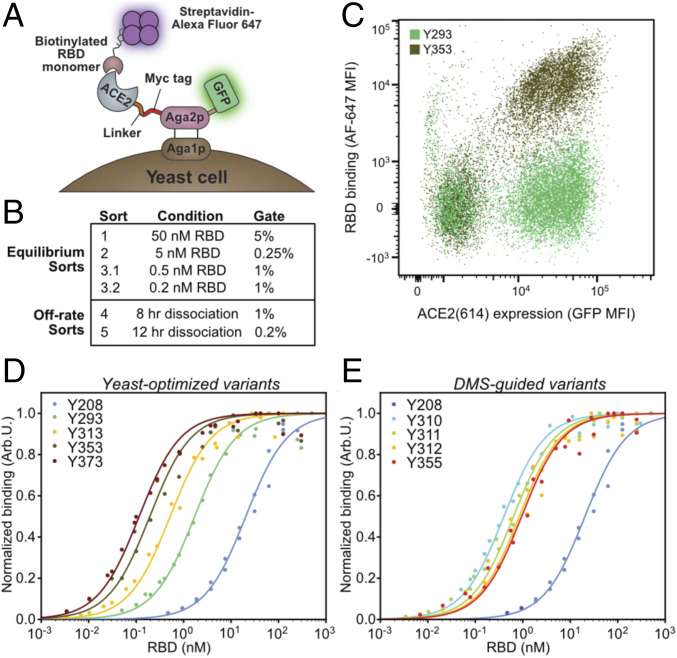
Affinity maturation by yeast surface display of pooled error-prone PCR libraries on designed ACE2 variants and DMS-guided design. (*A*) Monomeric ACE2(614) variants were expressed as Aga2p-GFP fusions on the surface of yeast cells. Binding of ACE2(614) to the spike RBD was quantified by incubation of the cells in a solution of biotinylated RBD, followed by staining the cells with streptavidin Alexa Fluor 647, washing the cells, and measuring fluorescent populations by flow cytometry. (*B*) Stringency was increased in each round of yeast display to enrich the cell population for ACE2(614) variants that bound the RBD tightly. Equilibrium sorts with decreasing RBD concentrations were used for sorts 1 to 3, and off-rate sorts with increasing dissociation times and decreasing gate size were used for the last two sorts. (*C*) Representative cell populations by flow cytometry for yeast expressing a tight binding ACE2(614) variant from sort 4 compared with yeast expressing the computationally designed parent, K31F/H34I/E35Q ACE2(614), incubated with 0.1 nM RBD monomer. The additional mutations in the sort 4 variant shift the cell population higher on the *y* axis due to tighter RBD binding. (*D*) On-yeast titration curves for cells expressing ACE2(614) variants chosen from sorts 3 (Y313), 4 (Y353), and 5 (Y373) that bind the RBD most tightly, compared with WT ACE2(614) (Y208) and K31F/H34I/E35Q ACE2(614) (Y293). (*E*) On-yeast titration curves for cells expressing the ACE2(614) variants generated by DMS-guided design, compared with WT ACE2(614) (Y208). Titration curves in *D* and *E* are fit to biological duplicates, shown as points. Variant names, mutations, and $K_{D,app}$ values are in *SI Appendix*, Table S3. MFI, median fluorescence intensity.

To further improve the binding affinity of the designed proteins for the spike RBD, we expressed a mutagenized library of ACE2(614) variants as Aga2p fusions for surface display in yeast cells, without the Fc domain to avoid avidity effects that might dominate affinity maturation. We chose four ACE2(614) variants as starting templates for a randomized yeast-displayed library, with the following mutations: H34V, N90Q, H34V/N90Q, and K31F/H34I/E35Q. Each of these variants, in addition to WT ACE2(614), was cloned as fusions to a Myc tag, Aga2p, and C-terminal enhanced GFP for a simple readout of induction and surface display level (Fig. 3*A*) (30). The expression of WT ACE2(614) was first induced on EBY100 cells in synthetic galactose medium supplemented with casamino acids (SGCAA) media at 20 °C and 30 °C, and we confirmed binding using biotinylated spike RBD-Fc and streptavidin Alexa Fluor 647 (*SI Appendix*, Fig. S3 *A* and *B*). Binding of the RBD was well correlated with GFP expression, precluding the need for Myc-tag (expression) staining (*SI Appendix*, Fig. S3 *A* and *B*).

Sixteen sublibraries of ACE2 residues 18 to 103 were made by homologous recombination into ACE2(614) using the four input templates, each mutagenized at four different rates using deoxyribonucleotide triphosphate (dNTP) analogues (31). After transformation into EBY100 cells for a total library size of 2.7 × $10^{7}$ members, sequencing of 24 presort clones showed an even distribution of mutations across residues 18 to 103 with representation from all four input sequences (*SI Appendix*, Fig. S3 *C* and *D*). We carried out sorts of increasing stringency using different concentrations of RBD monomer as outlined in Fig. 3*B* and *SI Appendix*, Fig. S4 and analyzed individual clones along the way. Sort 3 was performed with multiple binding stringencies and expression temperatures. For example, sorts of ACE2 induced at 30 °C did not show increased expression in subsequent rounds, but high-affinity clones were observed from both 500 pM (for sort 3.1) and 200 pM (for sort 3.2) equilibrium sorts. The sequences of 21 clones from an 8-h off-rate sort 4 did not show clonal convergence but were enriched in favorable mutations in agreement with published DMS data (24). A very stringent fifth sort, in which surface-displayed ACE2 was allowed to dissociate from RBD for 12 h at room temperature and only 0.2% of the cell population was collected, still did not result in sequence convergence but showed enrichment of one clone derived from the computationally designed K31F/H34I/E35Q ACE2(614) variant. The enriched ACE2(614) variant had the following seven mutations: Q18R, K31F, N33D, H34S, E35Q, W69R, and Q76R. Interestingly, a majority of sequences from sorts 4 and 5 were derived from the K31F/H34I/E35Q ACE2(614) parent, but many of these had mutations at I34 to serine or alanine (*SI Appendix*, Fig. S5). This observation suggested that while I34 led to an alternate design variant, the isoleucine was not always the ideal amino acid at this position. A small number of additional mutations appeared in variants originating from different parents, including F40L/S, N49D/S, M62T/I/V, and Q101R, while others appeared only in the K31F/H34I/E35Q background, such as L79P/F and L91P.

Following sorts 3 through 5, 18 to 24 individual yeast clones were picked from each sort for further characterization. After growth and induction, we analyzed each population for high-affinity mutants by staining with decreasing concentrations of the RBD monomer. The best mutants from each sort were sequenced (mutations are listed in *SI Appendix*, Table S3), and their $K_{D,app}$ values for the monomeric spike RBD were measured by on-yeast titrations (Fig. 3*D* and *SI Appendix*, Table S3). The best-characterized mutants from sorts 3.1, 3.2, 4, and 5 had affinities of 0.52, 0.45, 0.19, and 0.12 nM, respectively [between 39- and 170-fold higher affinity than WT ACE2(614)]. Although each sort contained a variety of mutants, the highest-affinity clones contained N33D and H34S mutations and were derived from the K31F/H34I/E35Q ACE2(614) variant. The low likelihood of multiple base mutations in a single codon in error-prone PCR mutagenesis likely favored the I34S mutation from this background; interestingly, ACE2 receptors from pangolin species that are hypothesized to be SARS-CoV-2 reservoirs also include a serine at position 34 (32, 33). ACE2 N33 does not directly contact the RBD in the crystal structure, but the enrichment of the N33D mutation in our affinity maturation was consistent with the enrichment of this point mutant in the DMS study (24).

As an orthogonal approach to generate affinity-enhanced ACE2 variants, we leveraged the results from the DMS experiment by Procko (24) to perform an additional round of DMS-guided computational design. Our original computational design strategy (Fig. 2 *A*–*C*) targeted alanine scan hot spots, as described. The DMS experiment of Procko (24) identified beneficial ACE2 point mutations in the ACE2–RBD interface at nonhot-spot positions, which could improve binding affinity by direct interactions with the RBD, as well as outside the binding interface, which might serve a stabilizing role. These two classes of mutations would not have been predicted by our computational design strategy. Thus, we performed another round of computational saturation mutagenesis at nonhot-spot ACE2 positions in the ACE2–RBD interface (A25, T27, K31), as well as the noninterface residue W69, to predict additional mutations. We generated a set of DMS-guided, computationally designed ACE2 variants with three to four mutations each that included at least two mutations outside the interface chosen directly from the DMS dataset, combined with one to two mutations from the computational saturation mutagenesis at nonhot-spot positions that were also enriched in DMS (*SI Appendix*, Fig. S6 and Table S3). These designed proteins were displayed on the surface of yeast as ACE2(614)-Aga2p fusions. We measured the $K_{D,app}$ of the DMS-guided ACE2(614) variants for the monomeric RBD to be between 0.4 and 1 nM by on-yeast titrations, which is between 21- and 51-fold higher than WT ACE2(614) (Fig. 3*E* and *SI Appendix*, Table S3). The ACE2(614) variant from DMS-guided computational design with the lowest $K_{D,app}$ had the following mutations: A25V, T27Y, H34A, and F40D.

**Fig. 4.**
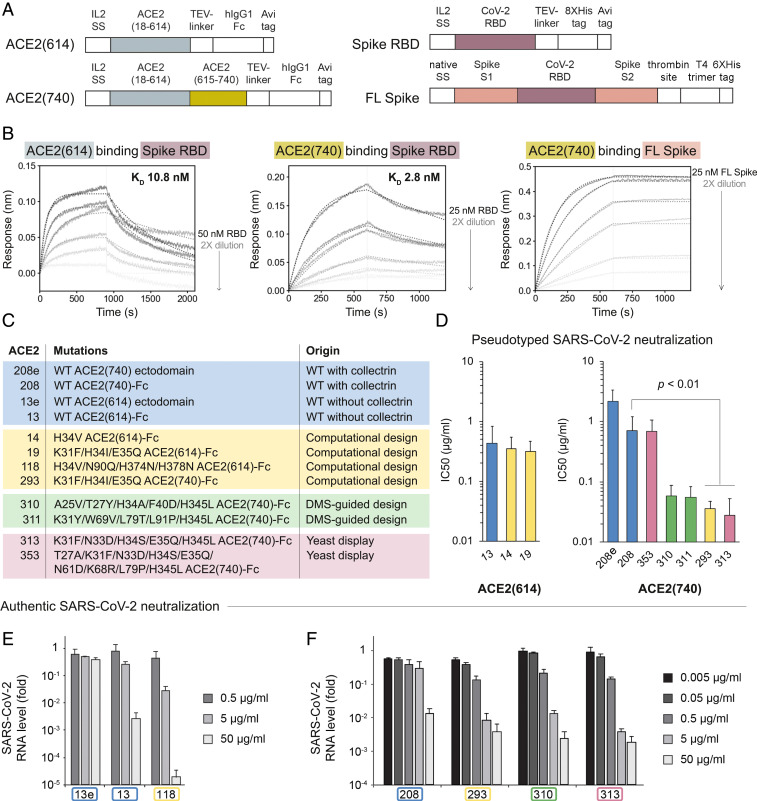
Increased stability, affinity, and avidity effects result in tighter binding between ACE2(740)-Fc and FL spike and potent viral neutralization. (*A*) Plasmid constructs for expression of ACE2(614) and ACE2(740) (*Left*) and spike in the monomeric RBD and full-length forms (*Right*). Avi tag, target sequence for intracellular biotinylation by BirA; hIgG1-Fc, human immunoglobulin 1 Fc domain; interleukin-2 (IL2) SS, IL2 signal sequence (cleaved); native SS, native spike signal sequence; thrombin site, thrombin cleavage site; T4 trimer, T4 bacteriophage fibritin trimerization motif; tobacco etch virus (TEV)-linker, TEV protease cleavage sequence and glycine-serine linker; 8X- or 6XHis tag, polyhistidine tag. (*B*) BLI measurements show decreased $K_{D}$ for ACE2(740)-Fc compared with ACE2(614)-Fc for the interaction with the spike RBD. Binding between ACE2-Fc and the FL spike protein results in ${a\;K}_{D}$ less than 100 pM due to very low off rates. Solid lines show response curves for twofold dilution titration spanning 0.37 to 50 nM RBD. Dotted lines show calculated fits. (*C*) Table of all ACE2 variants with scaffolds and mutations tested in pseudotyped and authentic SARS-CoV-2 viral neutralization assays, listed with their origin. (*D*) Fc fusion, inclusion of the collectrin domain, and affinity-enhancing mutations improve neutralization of ACE2 constructs against pseudotyped SARS-CoV-2 virus, except for misfolded variant 353. Error bars represent SDs over all technical replicates from two to four biological replicates. Biological replicates were separate experiments using different preparations of the ACE2 variant and pseudovirus, each with two or four technical replicates. Statistical significance with *P* < 0.01 was determined using a homoscedastic two-tailed *t* test. Authentic SARS-CoV-2 viral neutralization experiments with ACE2 variants in VeroE6 cells showed that (*E*) Fc fusion (CVD013) and addition of computationally predicted mutations (CVD118) enhance neutralization by greater than 50,000-fold over a control anti-GFP IgG antibody sample and that (*F*) inclusion of the collectrin domain, computationally predicted mutations (CVD293), DMS-guided mutations (CVD310), and mutations from affinity maturation in yeast (CVD313) enhance neutralization potency over a control anti-GFP IgG antibody sample to IC50 values of 73 to 136 ng/mL. *E* and *F* show results from different experiments in biological duplicate. Error bars represent the SEM.

We next characterized binding of WT, computationally designed, and affinity-matured ACE2 variants in different Fc fusion formats. Using BLI, we tested whether inclusion of the natural C-terminal ACE2 collectrin domain (residues 615 to 740) could improve the protein’s binding affinity for spike (Fig. 4*A*). A recent cryo-electron microscopy structure shows ACE2 as a dimer, with the collectrin domain connecting the extracellular peptidase domain of ACE2 to its transmembrane helix (34). The structure also reveals additional intercollectrin domain contacts and C-terminal contacts between peptidase domains (*SI Appendix*, Fig. S7). Fc fusions of WT ACE2 containing the collectrin domain were also recently shown to be more effective in blocking viral infection (29), perhaps by repositioning the ACE2 monomers for improved binding to spike. Furthermore, the three RBDs in the spike trimer can independently bind ACE2 (35, 36). We hypothesized that the inclusion of the ACE2 collectrin domain and the additional two spike RBDs would increase the strength of the ACE2–spike interaction through stabilization and avidity effects. We tested binding of ACE2-Fc with the collectrin domain [ACE2(740)-Fc] to spike RBD and full-length spike (FL spike) using BLI. Compared with the ACE2(614)-Fc interaction with the spike RBD, we indeed saw a 3.7-fold decrease in the monovalent $K_{D}$ of WT ACE2(740)-Fc for the spike RBD and a dramatic decrease in the $K_{D}$ of ACE2-Fc with or without the collectrin domain for FL spike (Fig. 4*B* and *SI Appendix*, Fig. S8 *A–E*). Both WT and computationally designed ACE2(740)-Fc binding interactions with the FL spike were too tight to be accurately measured by BLI due to the massively decreased off rates (Fig. 4*B* and *SI Appendix*, Fig. S8 *A–E*). ACE2(740)-Fc variants from DMS-guided design and affinity maturation in yeast also had greatly reduced off rates for monovalent interactions with the RBD (*SI Appendix*, Fig. S8 *F* and *G*). The highest-affinity mutants from our yeast display campaign were poorly expressed as Fc fusions, indicating that despite numerous reports of stabilizing mutations from yeast display, ACE2 variant expression on yeast did not translate to well-folded soluble protein (37, 38).

The soluble domain of ACE2 converts angiotensin II to angiotensin(1–7), a vasodilator, and was shown to be safe in clinical trials (12, 13). The RBD binds outside the enzyme active site. We inactivated the peptidase activity of ACE2 to avoid off-target vasodilation effects without affecting the binding affinity for the RBD (Fig. 2 *D* and *E* and *SI Appendix*, Fig. S2) (11). Our original inactivation mutations, H374N and H378N, ablated ${Zn}^{2+}$ binding. However, protein stability is also an important factor to consider in engineering an optimal ACE2-based therapeutic scaffold. The relative destabilization of active site mutations is difficult to predict using computational methods in the absence of multiple structures representing the catalytic cycle. Incorporation of the ACE2 collectrin domain in our Fc-fused constructs improved the apparent melting temperature of the ACE2-Fc variants as measured by circular dichroism (CD) spectroscopy, but the H374N/H378N enzymatic inactivation mutations were destabilizing (*SI Appendix*, Fig. S9). Therefore, we adapted the ACE2(740)-Fc scaffold to include the inactivation mutation H345L instead, which is important for substrate binding and is not destabilizing (*SI Appendix*, Figs. S2 and S9) (39). H345L ACE2(740)-Fc does not have detectable peptidase activity in an activity assay and does not impact binding to the spike RBD in a BLI assay (*SI Appendix*, Fig. S10), but it maintains the thermal stability of WT ACE2.

We found that the binding affinity improvements to ACE2 were robust to the method of measurement (BLI vs. binding on yeast) and well correlated with neutralization efficacy. To evaluate their efficacy in neutralizing SARS-CoV-2 infections, several affinity-improved ACE2 variants from computational design, DMS-guided design, and yeast display were expressed in the H345L ACE2(740)-Fc format, purified, and assayed for viral neutralization against pseudotyped lentivirus and authentic SARS-CoV-2 (Fig. 4 *C*–*F*). In the pseudotyped viral neutralization assay in ACE2-expressing human embryonic kidney (HEK) cells, different ACE2(740)-Fc molecules with mutations derived from computational design, DMS-guided design, and affinity maturation using yeast surface display neutralized SARS-CoV-2 with IC50 values of 58, 55, 36, and 28 ng/mL, demonstrating multiple paths to significant improvements in efficacy (variants 310, 311, 293, and 313, respectively, in Fig. 4*D*; *SI Appendix*, Table S4). We also confirmed that the affinity-enhanced ACE2 variant 353 [T27A/K31F/N33D/H34S/E35Q/N61D/K68R/L79P/H345L ACE2(740)-Fc] does not effectively neutralize SARS-CoV-2, despite this molecule’s enrichment in the yeast display campaign and low $K_{D,app}$, possibly because the variant is unstable or otherwise misfolded (Fig. 4*D* and *SI Appendix*, Fig. S11). Taken together, the neutralization data revealed that the mutations to ACE2 from computational design and affinity maturation, addition of the collectrin domain, and fusion to the Fc domain significantly improve neutralization over unmodified ACE2(740). The unmodified ACE2(740) (Fig. 4 *C* and *D*, variant 208e) is similar to the molecule APN01 that is currently in clinical trials for treating SARS-CoV-2 infections (Fig. 4*D* and *SI Appendix*, Fig. S11 and Table S4) (16, 17).

Data from viral neutralization assays in which bona fide SARS-CoV-2 was used to infect VeroE6 cells in a biosafety level 3 facility closely reflected the results from the pseudotyped viral neutralization assays. We determined that fusion of the Fc domain to ACE2(614) improved neutralization by 370-fold over monomeric ACE2(614), and additional inclusion of computationally predicted mutations H34V and N90Q improved neutralization by more than 50,000-fold over an anti-GFP IgG control, at the highest concentration tested (Fig. 4*E*, 50 μg/mL). The IC50 for this intermediate affinity-enhanced ACE2(614)-Fc variant, CVD118, was less than 0.5 μg/mL.

Addition of the ACE2 collectrin domain further improved neutralization potency, with ACE2(740)-Fc variants originating from computational design, DMS-guided design, and affinity maturation in yeast demonstrating efficient neutralization in the neutralization assay using bona fide SARS-CoV-2 (Fig. 4*F*). WT ACE2(740)-Fc (variant 208), computationally designed variant 293, DMS-guided design 310, and yeast affinity-matured variant 313 were tested at concentrations from 0.005 to 50 μg/mL. Variants 293, 310, and 313 each considerably diminished viral RNA levels at concentrations starting at 0.05 μg/mL, while WT ACE2(740)-Fc only had neutralization efficacy at 5 μg/mL. Variants 310 and 313 displayed the most neutralization potency, with IC50s of approximately 89 and 73 ng/mL, respectively (Fig. 4*F* and *SI Appendix*, Table S4). This neutralization potency is comparable with recently reported antibodies isolated from convalescent COVID-19 patients (6, 40). None of the ACE2 variants induced cytotoxicity in uninfected cells at the concentrations used in the neutralization assay (*SI Appendix*, Fig. S12). Additional live SARS-CoV-2 neutralization experiments with shorter incubation times (16 h rather than 26 h) and a different SARS-CoV-2 strain were conducted in a different laboratory to ensure reproducibility and measure the effect of the affinity-enhanced ACE2(740)-Fc molecules on viral entry more directly and yielded similar results: IC50 values were in the range of 0.1 to 1 μg/mL for variant 293 and lower for variants 310 and 313 (*SI Appendix*, Fig. S13).

The inclusion of the ACE2 collectrin domain and the human IgG Fc domain dramatically increased the neutralization potency of the ACE2 variants through improved affinity, stability, and avidity (Fig. 4 and *SI Appendix*, Figs. S2 and S7). The Fc fusion results in ACE2 dimerization, but the collectrin domain may serve to position the ACE2 molecules closer together than would be achieved with the Fc alone (34). We also observed that the interaction of dimeric ACE2-Fc with the full-length trimeric spike protein is stronger than with the monomeric RBD due to avidity effects. As a combined result of these effects, the use of the H345L ACE2(740)-Fc scaffold is central to the neutralization potency of the affinity-enhanced variants.

**Fig. 5.**
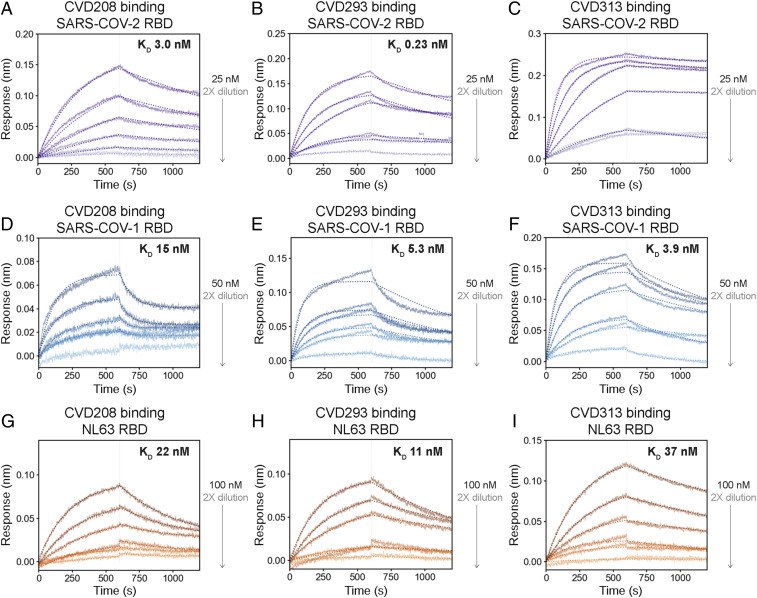
WT and engineered ACE2(740)-Fc bind the SARS-CoV-1 RBD and the HCoV-NL63 RBD. Representative BLI measurements for (*A*, *D*, and *G*) WT ACE2(740)-Fc, (*B*, *E*, and *H*) K31F/H34I/E35Q ACE2(740)-Fc, and (*C*, *F*, and *I*) K31F/N33D/H34S/E35Q/H345L ACE2(740)-Fc interactions with the SARS-CoV-2 RBD (*A–C*), the SARS-CoV-1 RBD (*D–F*), and the HCoV-NL63 RBD (*G–I*) at concentrations of 0.375 to 100 nM RBD, with the highest RBD concentration tested and $K_{D}$ values as indicated. Dotted lines show calculated fits. The extremely slow off rate observed in *C* precluded $K_{D}$ determination.

ACE2-based therapeutics could be used to treat other respiratory infections with ACE2-dependent cell entry mechanisms, such as those caused by SARS-CoV-1 and human coronavirus (HCoV)-NL63 coronaviruses (25, 41). We tested whether WT ACE2(740)-Fc and our most robustly expressed ACE2(740)-Fc variants could bind the SARS-CoV-1 and NL63 spike RBDs (Fig. 5). Indeed, WT ACE2(740)-Fc, a receptor trap from affinity maturation in yeast (variant 313, with K31F, N33D, H34S, E35Q, and enzymatic inactivation mutation H345L) and its computationally designed parent (variant 293, K31F, H34I, E35Q) bound with nanomolar $K_{D}$ to the SARS-CoV-1 RBD (Fig. 5 *D*–*F*) and tens of nanomolar $K_{D}$ to the NL63 RBD (Fig. 5 *G*–*I*), which is close to previous observations for the NL63 RBD–WT ACE2 interaction (41). The weaker binding affinity for the NL63 RBD interaction with ACE2-Fc is likely due to its low structure/sequence similarity to the SARS-CoV-2 RBD, while the SARS-CoV-1 and SARS-CoV-2 RBDs have similar structures and 73% sequence identity (2) (*SI Appendix*, Fig. S14). In contrast, the ACE2 variants did not bind appreciably to the Middle East respiratory syndrome (MERS) RBD up to 150 nM (*SI Appendix*, Fig. S15); MERS-CoV particles enter cells not via ACE2 but through interactions with the dipeptidyl pepdidase IV (DPP4; also known as CD26) membrane protein (42).

## Discussion

Engineered receptor traps have been used extensively as biotherapeutics for binding vascular endothelial growth factor, tumor necrosis factor alpha, and other cytokines and produced at therapeutic scale (31, 43–45). Affinity is often sufficiently improved by simply presenting these proteins in dimeric form fused to human-engineered Fc to achieve avidity and afford long half-lives. There are no Food and Drug Administration-approved examples of receptor traps as antiviral biologics (46, 47), although some have entered clinical trials for HIV treatment (48). Here, we show it is possible using both computational design and selection methods to dramatically improve the binding for dimeric ACE2 for the SARS-CoV-2 spike RBD to the range of high-affinity antibodies.

We used several computational design methods in Rosetta to predict mutations that could enhance the affinity of ACE2 for the SARS-CoV-2 RBD, with one innovation: following computational saturation mutagenesis, we proceeded to the local redesign step with the lowest-energy point mutant (H34V) as well as the highest-energy point mutant (H34I). Our hypothesis was that mutating and optimizing the positions of surrounding residues would allow us to identify alternative low-energy solutions for the ACE2–RBD interaction, which proved to be the case. We experimentally validated that the variant designed from H34I ACE2 improved binding affinity, pseudotyped viral neutralization, and authentic viral neutralization in two independent laboratories compared with other computationally predicted mutations, despite the fact that no single mutation in this design conferred a fitness advantage in DMS (Figs. 2 and 4 and *SI Appendix*, Table S1) (24). In our strategy, we did not include a sublibrary based on the purely WT sequence, instead opting for N90Q, which both removes the variable of heterogenous glycosylation in yeast and enhances affinity. Strikingly, the computationally designed H34I-containing variant outcompeted the N90Q ACE2 mutant and was the parent of the most effective ACE2 molecules to emerge from affinity maturation (*SI Appendix*, Fig. S5 and Table S3). It is highly unlikely that the K31F, H34I, and E35Q mutations would have arisen using a purely experimental evolution strategy due to mutational bias in error-prone PCR. While yeast display affinity maturation can be done in the span of several weeks, developing the computational design pipeline and testing the resulting designed ACE2 variants only took 1 week. This demonstrates the potential benefits of developing computational design methods that include steps to diversify solutions.

Engineered ACE2 receptor traps present key advantages in treating SARS-CoV-2 infections. Several groups have isolated antibodies from convalescent patients, confirmed their neutralization potencies, characterized their affinities to the RBD, and in some cases, determined their structures. A subset of the neutralizing antibodies blocks ACE2, while the remainder binds spike epitopes outside the ACE2 binding interface (3, 4, 6–10, 18–20, 40). By contrast, ACE2 receptor traps directly compete with the essential viral entry mechanism. Recent reports indicate that RBD binding antibodies are also susceptible to diminished affinity at lower pH, which could lead to lower viral neutralization and potentially, reinfection (36). Finally, viral escape mutations can render antibody therapeutics ineffective, but escape mutations that reduce the efficacy of an ACE2 receptor trap binding are also likely to reduce viral invasion. Many of the affinity-enhancing mutations that we described are in the receptor binding site, and so, it is conceivable that they could be selectively targeted by a mutant virus; however, we show that even the NL63 RBD can still bind our ACE2 variants despite its significant sequence and structure divergence from the SARS-CoV-2 RBD. Receptor traps based on WT ACE2 (11, 49), antibody fusions (50), or affinity-enhanced mutants (29), which include naturally occurring mutations that were also found in our engineering efforts (33), have also been reported to neutralize pseudovirus and spike-based cell fusion. Our work harnesses the power of protein engineering approaches to build on these lines of research by engineering orders of magnitude higher affinity and demonstrating potent neutralization of authentic SARS-CoV-2 virus. We also anticipate that engineered ACE2 receptor traps could synergize in a mixture with neutralizing antibodies that bind the RBD outside the ACE2 binding site to treat viral infections (20).

The systematic two-pronged affinity optimization approach for engineering ACE2 receptor traps was achieved by a small team in several months, which is comparable with antibody isolation from convalescent patients or selection by in vitro methods (10). Thus, it represents a rapid and orthogonal approach to generating therapeutic candidates for treating future viral pandemics, without any prerequisite for an infected human population. Engineered receptor traps can be stockpiled as potentially useful drug candidates for multiple viruses that use the same port of entry as we show for HCoV-NL63 and pandemic viruses SARS-CoV-1 and SARS-CoV-2 and decrease the likelihood of viral resistance, although the impact of mutations on the immunogenicity of the molecule is unknown and would need to be monitored in human subjects. Our study on ACE2 provides a systematic road map to redesigning an entry receptor as a therapeutic, and we believe the same strategy could be applied to other entry receptors such as DPP4 for treating MERS and aminopeptidase N (ANPEP) for treating upper respiratory tract infections by HCoV-229E (42, 51).

## Materials and Methods

Structural modeling and computational protein design are in *SI Appendix*, *Supplemental computational methods: Command lines and input files* (command lines and input files for each section). We used the 2019.38 release of Rosetta 3.12 (Git SHA1 hash number: 2019.38.post.dev+231.master.04d3e581085 04d3e581085629b0f0c46f1e1aef9e61978e0eeb).

### Preparation of ACE2–Spike Structure for Modeling.

To model ACE2–spike interactions, we used the 2.50-Å resolution X-ray structure of the spike RBD complexed with the soluble extracellular domain of ACE2 as determined by Wang et al. (23) (Protein Data Bank [PDB] ID code 6LZG). This structure was downloaded from the PDB, relaxed with coordinate constraints on backbone and side chain heavy atoms, and minimized in Rosetta without constraints using default options using the beta_nov16 score function (command lines are in *SI Appendix*, *Supplemental computational methods: Command lines and input files*).

### Identification of ACE2 Residues That Contribute to Binding in the ACE2–Spike Interface and Are Chosen for Design.

To determine which residues contribute most strongly to the ACE2–spike interaction, we used the Robetta Computational Interface Alanine Scanning Server (26, 27) (publicly available at http://robetta.bakerlab.org/alascansubmit.jsp) to perform a computational alanine scan on the relaxed and minimized input structure of the complex. The alanine scan identified 18 ACE2 residues in the interface, 6 of which had DDG(complex) values greater than one. These hot spots represent amino acid side chains that are predicted to significantly destabilize the interface when mutated to alanine. *SI Appendix*, Table S1 shows the full results of computational alanine scanning.

We used two metrics to determine which hot spots could most likely be mutated to improve the ACE2–spike binding affinity: 1) the total per-residue energy as evaluated by the Rosetta score function to be the sum of all one-body and half the sum of all two-body energies for that residue and 2) the total contribution of the residue to the interface energy, which is the sum of pairwise residue energies over all residue pairs (R1, R2) where R1 belongs to ACE2 and R2 belongs to the spike RBD. We classified hot-spot residues that had total residue energies in the top 30% of all residues in ACE2 as well as total cross-interface interaction energies greater than 0.5 REU as residues in the ACE2–spike interface to be targeted for design. These residues were H34, Q42, and K353.

### Computational Saturation Mutagenesis at Targeted ACE2 Interface Residue Positions.

We systematically computationally mutated H34, Q42, and K353 in ACE2 to every other amino acid except cysteine, allowing all residues with side chain heavy atoms in ACE2 or spike within 6 Å of the mutated position to repack (change rotameric conformation); minimized the entire complex; and recalculated all of the pairwise interaction energies across the ACE2–spike interface and various interface metrics. These interface metrics were the solvent-accessible surface area buried at the interface; the change in energy when ACE2 and spike RBD are separated vs. when they are complexed; the energy of separated chains per unit interface area; the number of buried and unsatisfied hydrogen bonds at the interface; a packing statistic score for the interface; the binding energy of the interface calculated with cross-interface energy terms; a binding energy calculated using Rosetta’s centroid mode and score 3 score function; the number of residues at the interface; the average energy of each residue at the interface; the energy of each side of the interface; the average per-residue energy for each side of the interface; the average energy of a residue in the complex; the total number of cross-interface hydrogen bonds; and the interface energy from cross-interface hydrogen bonds. Each point mutation was modeled five times using this protocol, and the lowest of the summed cross-interface pairwise interaction energies from the five trials was used for comparison with the WT interface value.

### Redesign of ACE2 Interface Residues Incorporating H34V or H34I Mutations.

We generated two additional sets of models to select constructs for experimental testing that incorporated the most and least energetically favorable point mutations to H34 from the computational saturation mutagenesis. In these simulations, we first mutated H34 to either valine or isoleucine. Residues within 6 Å of the interface were repacked, and minimization was applied to the interface backbone and side chain torsion angles. A flexible backbone design algorithm (Coupled Moves) (28) was applied to allow neighboring ACE2 residues 30, 31, 35, and 38 to change amino acid identities while allowing ACE2 residues 29, 32, 33, 34, 36, and 37 and RBD residues 416, 417, 418, 452, 453, 455, 456, 492, 493, and 494 to repack. Changes in the positions of backbone atoms were allowed for ACE2 residues 30 to 38 and RBD residues 417, 453 to 455, and 493. The whole complex in the lowest-energy solution for the redesigned interface was again repacked and minimized, and the final structure was scored. The lowest of the summed cross-interface pairwise interaction energies from 20 trials was used.

### Determination of Binding Affinity to Spike Using BLI.

In our BLI experiments, the biotinylated ACE2 variant is tethered to an optically transparent biosensor tip by a biotin–streptavidin interaction, and spike is present as the analyte in solution in the microplate. ACE2 gene sequences and mutations are listed in *SI Appendix*, Appendix S1. Affinity measurements were carried out at room temperature using an Octet RED96 system and streptavidin-coated biosensor tips (Pall ForteBio). Biotinylated ACE2 variants were diluted to 10 nM in phosphate buffered saline (PBS) with 0.2% bovine serum albumin (BSA) and 0.05% Tween-20 (PBS-T), pH 7.4, to be used as the antigen. Antigen-bound streptavidin tips were washed in PBS-T, pH 7.4; separately exposed to the spike solutions at concentrations ranging from 0 to 100 nM spike in the same buffer during an association period; and then returned to the washing well during a dissociation period. The binding protocol was as follows: rinse tips in PBS-T, 60 s; load tips with antigen, 180 s; establish baseline by rinsing tips in PBS-T buffer, 180 s; association with analyte, 600 s; dissociation in baseline wells, 900 s. Raw data were fit to 1:1 binding curves in Octet Data Analysis HT software version 10.0 using curve-fitting kinetic analysis with global fitting. The theoretical equilibrium binding signal response data (R equilibrium) were normalized by the steady-state group maximum response values, and the steady-state affinity was determined using the Hill equation. Noncooperative binding kinetics were assumed. All fits to BLI data had $R^{2}$ (goodness of fit) > 0.90.

### Determination of Stability by CD Spectroscopy with Thermal Denaturation.

CD data were collected on a Jasco J-710 spectrometer using purified ACE2 variant solutions in 1-mm quartz cuvettes. ACE2 variants were diluted in 300 μL PBS, pH 7.4, to concentrations ranging from 2 to 3 μM. Melting curves at 225 nm were measured by increasing the temperature from 25 °C to 80 °C using a rate of 1 °C per minute. CD spectra from 200 to 280 nm were measured at 25 °C and 80 °C. Melting curve data were normalized using an average of the before-melt baseline as 0% and an average of the after-melt baseline as 100%, and the apparent melting temperature, $T_{m,app}$, was determined to be the temperature at which 50% of the protein was denatured between these points. Melting was irreversible for WT ACE2.

### ACE2 Proteolytic Activity Assay.

Hydrolysis of (7-methoxycoumarin-4-yl)acetyl-Ala-Pro-Lys(2,4-dinitrophenyl)-OH (Mca-APK-DNP; Enzo Life Sciences) was used to quantify ACE2 peptidase activity; 50 μL each of solutions of ACE2 variants (diluted to 0.3 nM) and 100 μM Mca-APK-DNP in 50 mM 2-(*N*-morpholino)ethanesulfonic acid (MES) buffer, 1 M NaCl, and 10 μM ZnCl_2_ were mixed in a 96-well plate. Fluorescence increase over time was monitored (320 nm excitation/405 nm emission).

### Yeast Transformations.

Electrocompetent EBY100 were prepared by the method of Benatuil et al. (52). *SI Appendix* has full methods.

### Analysis of Yeast Library.

To analyze sequences, plasmids were isolated from yeast using a modified version of Singh and Weil (53). *SI Appendix* has full methods.

### SARS-CoV-2 Pseudotyped Virus Neutralization Assay.

Pseudotyped reporter virus assays were conducted as previously described (54). Pseudovirus plasmids were a gift from the laboratory of Peter Kim, Stanford University, Stanford, CA. HEK-ACE2 cells were cultured in D10 media (Dulbecco’s modified Eagle medium + 1% penicillin/streptomycin solution + 10% heat-inactivated fetal bovine serum [FBS]). Briefly, spike pseudovirus with a luciferase reporter gene was prepared by transfecting plasmids into HEK-293T cells. After 24 h, the transfection solution was replaced with D10 media, and the virus was propagated for 48 h before harvest and filtration of supernatants. To titer each virus batch, HEK-ACE2 was seeded at 10,000 cells and infected with twofold dilution series of stock virus for 60 h. Cellular expression of luciferase reporter indicating viral infection was determined using the Bright-Glo Luciferase Assay System (Promega). For neutralization assays, virus stock was diluted to 3 to 5 × $10^{5}$ luminescence units. Pseudovirus neutralization assays were performed on HEK-ACE2 cells seeded at 10,000 cells per well in 40 μL of D10. To determine IC50, blocker dose series were prepared at 3× concentration in D10 media. In 96-well format, 50 μL of 3× blocker and 50 μL of virus were mixed in each well, and the virus and blocker solution was incubated for 1 h at 37 °C. After preincubation, 80 μL of the virus and blocker inoculum were transferred to HEK-ACE2 cells. Infection was carried out for 60 h at 37 °C, at which point the intracellular luciferase signal was measured using the Bright-Glo Luciferase Assay (Promega). Neutralization was determined by normalizing the luminescent signal to the average value of the no blocker control. IC50 average values and SDs were calculated using four to eight technical replicates (repeated experiments run at the same time) from two to four biological replicates (using different virus stocks and different ACE2 variant preparations).

### SARS-CoV-2 Neutralization Assay at Biosafety Level 3.

VeroE6 cells were plated in a 96-well plate at 1.2 × $10^{4}$ cells per well and incubated overnight. At biosafety level 3, blocking or control (anti-GFP antibody) proteins and the Canadian clinical isolate of SARS-CoV-2 (SARS-CoV-2/CANADA/VIDO 01/2020) were mixed in fresh media supplemented with 3% FBS (Gibco) and preincubated for 1 h at 37 °C. The cells were washed once with PBS and infected at the multiplicity of infection (MOI) of 0.1 with the proteins for 1 h at 37 °C and 5% ${CO}_{2}$. Next, the mix was removed, and the cells were washed twice with PBS. Complete culture medium with the proteins was added, cells were added to each well, and cells were incubated at 37 °C and 5% ${CO}_{2}$ for 24 h followed by cell lysates collection for viral quantitation by qPCR. Mock cells were incubated with the culture supernatant from uninfected VeroE6.

### SARS-CoV-2 qRT-PCR Assay.

For RNA analysis, total RNA was extracted using the NucleoSpin RNA kit (Macherey-Nagel) following the manufacturer’s protocol. Total RNA was reverse transcribed using 0.5 to 1 μg of total RNA and ImProm-II Reverse Transcriptase (Promega) according to the manufacturer’s protocol. qRT-PCR was performed with PerfeCTa SYBR Green SuperMix (Quanta BioSciences) in the CFX96 Touch Real-Time PCR Detection System (Bio-Rad). The cycling conditions were 45 cycles of 94 °C for 30 s, 55° C for 60 s, and 68° C for 20 s. Gene expression (fold change) was calculated using the 2(−ΔΔCT) method using human β-actin messenger RNA transcript as the internal control. The following forward and reverse primer pairs were used for PCR: β-actin 5′-TGGATCAGCAAGCAGGAGTATG-3′ and 5′-GCATTTGCGGTGGACGAT-3′, SARS-CoV-2 spike 5′-CAATGGTTTAACAGGCACAGG-3′ and 5′-CTCAAGTGTCTGTGGATCACG-3′ (2).

### Additional SARS-CoV-2 Neutralization Assays at Biosafety Level 3.

Results in *SI Appendix*, Fig. S13 were generated from live SARS-CoV-2 neutralization assays performed as previously described (15) at the University of California, San Francisco. Of note, the clinical strain used in the assay was SARS-CoV-2 virus clinical isolate 2019-nCoV/USA-WA1/2020 (BEI resources), and the infection duration was 16 h instead of 24 h.

### Cytotoxicity Assay.

The CellTiter-Glo Luminescent Cell Viability Assay (Promega) was used for quantitation of adenosine triphosphate (ATP) in cultured cells. Cells lysates were assayed after mixing 100 μL of complete media with 100 μL of reconstituted CellTiter-Glo Reagent (buffer plus substrate) following the manufacturer’s instructions. Samples were mixed by shaking the plates, after which luminescence was recorded with a GloMax Explorer Model GM3510 (Promega) 10 min after adding the reagent.

### Supporting Information.

Detailed computational methods, additional experimental methods, supporting tables, and supporting figures are available in *SI Appendix*.

## Supplementary Material

Supplementary File

## Data Availability

All study data are included in the article and *SI Appendix*.
