# Supplementary material for: Engineered ACE2 receptor traps potently neutralize SARS-CoV-2
Source: Proc Natl Acad Sci U S A. 2020 Oct 22;117(45):28046–55. doi: 10.1073/pnas.2016093117 (PMC7668070; doi:10.1073/pnas.2016093117)
Supplement: Supplementary File [file pnas.2016093117.sapp.pdf]

SI appendix for

Engineered ACE2 receptor traps potently neutralize SARS-CoV-2

www.pnas.org/cgi/doi/10.1073/pnas.2016093117

Supplemental computational methods: command lines and input files

Preparation of ACE2-spike structure for modeling.

Command line for relax:

```
~/Rosetta/source/bin/relax.macosclangrelease -in:file:s 6lzg.pdb -database
~/Rosetta/database -relax:constrain_relax_to_start_coords -
relax:coord_constrain_sidechains -out:suffix _relaxed -beta_nov16 -
corrections::beta_nov16
```

Command line for minimize:

```
~/Rosetta/source/bin/minimize.macosclangrelease -in:file:s
6lzg_relaxed_0001.pdb -database ~/Rosetta/database -out:suffix _min -
beta_nov16 -corrections::beta_nov16
```

Identification of ACE2 residues that contribute to binding in the ACE2-RBD interface and are chosen for design.

Command line for determining the energy of each pairwise interaction across the ACE2-Spike interface:

```
~/Rosetta/source/bin/interface_energy.macosclangrelease -in:file:s
6lzg_rm.pdb -face1 face1_ -face2 face2_ -beta_nov16 -corrections::beta_nov16
```

Computational saturation mutagenesis at selected ACE2 interface residue positions.

Example command line for running saturation mutagenesis protocol using RosettaScript XML (1):

```
~/Rosetta/Rosetta/main/source/bin/rosetta_scripts.linuxgccrelease -
parser:protocol H34A.xml -in:file:s ../6lzg_rm.pdb @../flags.txt -database
/home/anum/Rosetta/Rosetta/main/database -out:suffix _H34A
```

Example XML:

```
<ROSETTASCRIPTS>

    <SCOREFXNS>
      <ScoreFunction
            name="beta"
            weights="beta_nov16"/>
    </SCOREFXNS>

    <TASKOPERATIONS>
    </TASKOPERATIONS>

    <MOVERS>
      <InterfaceAnalyzerMover
            name="int_ddG"
            scorefxn="beta"
            fixedchains="A_B"/>
```

```
        <MutateResidue
              name="mutate_residue_1"
              target="34A"
              new_res="ALA"/>
        <MinMover
              name="minimize"
              scorefxn="beta"
              chi="1"
              bb="1"
              tolerance="0.005"/>
        <RepackMinimize
              name="repack_interface"
              scorefxn_repack="beta"
              scorefxn_minimize="beta"
              repack_partner1="1"
              repack_partner2="1"
              design_partner1="0"
              design_partner2="0"
              interface_cutoff_distance="6.0"
              repack_non_ala="1"
              minimize_bb="1"
              minimize_rb="1"
              minimize_sc="1"
              optimize_fold_tree="1"/>
    </MOVERS>

    <PROTOCOLS>
      <Add mover_name="mutate_residue_1"/>
      <Add mover_name="repack_interface"/>
      <Add mover_name="minimize"/>
      <Add mover_name="int_ddG"/>
    </PROTOCOLS>

</ROSETTASCRIPTS>
```

Example flags file:

```
-packing
    -ex1
    -ex1aro
    -extrachi_cutoff 0
    -ex2
-nstruct 5
-overwrite
-mute core.util.prof
-mute core.io.database
-corrections::beta_nov16
```

Redesign of ACE2 interface residues incorporating H34V or H34I mutations.

Example command line for running Coupled Moves using RosettaScript XML (1, 2):

```
~/Rosetta/Rosetta/main/source/bin/rosetta_scripts.linuxgccrelease -
parser:protocol H34V.xml @H34_flags.txt -database
/home/anum/Rosetta/Rosetta/main/database -out:suffix _CM
```

Example XML:

```
<ROSETTASCRIPTS>

    <SCOREFXNS>
      <ScoreFunction
            name="beta"
            weights="beta_nov16"/>
    </SCOREFXNS>

    <TASKOPERATIONS>
      <ReadResfile
            name="resfile"
            filename="H34.res"/>
    </TASKOPERATIONS>

    <MOVERS>
      <InterfaceAnalyzerMover
            name="int_ddG"
            scorefxn="beta"
            fixedchains="A_B"/>
      <MutateResidue
            name="mutate_residue_1"
            target="34A"
            new_res="VAL"/>
      <MinMover
            name="minimize"
            scorefxn="beta"
            chi="1"
            bb="1"
            tolerance="0.005"/>
      <RepackMinimize
            name="repack_interface"
            scorefxn_repack="beta"
            scorefxn_minimize="beta"
            repack_partner1="1"
            repack_partner2="1"
            design_partner1="0"
            design_partner2="0"
            interface_cutoff_distance="6.0"
            repack_non_ala="1"
            minimize_bb="1"
            minimize_rb="1"
            minimize_sc="1"
            optimize_fold_tree="1"/>
      <CoupledMovesProtocol
            name="coupled_moves"
            task_operations="resfile"/>
    </MOVERS>

    <PROTOCOLS>
```

```
      <Add mover_name="mutate_residue_1"/>
      <Add mover_name="repack_interface"/>
      <Add mover_name="coupled_moves"/>
      <Add mover_name="repack_interface"/>
      <Add mover_name="minimize"/>
      <Add mover_name="int_ddG"/>
    </PROTOCOLS>

</ROSETTASCRIPTS>
```

Example flags file:

```
-in
    -file
        -s 6lzg_rm.pdb

-packing
    -ex1
    -ex1aro
    -extrachi_cutoff 0
    -ex2
-number_ligands 0
-coupled_moves
    -initial_repack false
    -ligand_mode false
    -ligand_weight 0.0
-resfile H34.res
-nstruct 20
-min_pack true
-beta_nov16
-overwrite
-mute core.util.prof
-mute core.io.database
```

Example resfile:

```
NATRO
START

29 A NATAA
30 - 31 A ALLAAxc
32 - 34 A NATAA
35 A ALLAAxc
36 - 37 A NATAA
38 A ALLAAxc
39 A NATAA
416 - 418 B NATAA
452 - 456 B NATAA
492 - 494 B NATAA
```

Supplemental experimental methods

Cloning.

SARS-CoV-2 spike RBD and ACE2 variants were cloned into a pFUSE-based vector for mammalian expression using the Gibson method, transformed into XL10-Gold cells, and grown on low-salt LB + 25 µg/ml Zeocin. The genes encoding the SARS-CoV-2 RBD (328-533), ACE2(18-614) or ACE2(18-740) were inserted between two SpeI sites with a 5' mutated IL-2 signal sequence for secretion and 3' sequences for Gly-Ser linker, TEV protease cut site, human IgG1 hinge and Fc, and AviTag. The RBD monomer was also cloned into a similar construct where the Fc domain was replaced with an 8XHis tag. ACE2 point mutations were made by stitching 5' and 3' fragments encoding the desired mutation by PCR and inserting the resulting gene into ACE2 vectors digested with KasI and BsiWI with the Gibson method. Wild-type ACE2(18-614) was inserted into NheI/BamHI-double digested pCL2 as follows: fragments encoding amino acids 18-105 and 106-614 were PCR-amplified with 25-bp Gibson overlap regions introducing a silent mutation at S105 to introduce a new BamHI site, and a 3' silent mutation to eliminate the BamHI site in pCL2. These fragments were stitched together by PCR and inserted into pCL2, transformed into XL10-Gold, and grown on LB + 50 µg/ml carbenicillin. ACE2 variants in pCL2 were exchanged by digesting this vector with NheI/BamHI. ACE2 variants from yeast display were amplified and cloned into the ACE2(18-740)-Fc fusion vector in between the BlpI restriction sites on the IL-2 signal peptide and in the ACE2 gene.

Transfections.

A cell line derived from Expi293 cells expressing an ER-localized biotin ligase (BirA) gene was grown in Expi293 media (Thermofisher Scientific) supplemented with 100 µM biotin (GoldBio) under 8% CO_2 at 37 °C and used for all protein expressions. Transfections were carried out in 30 ml media as described in the Expi293 manual: 75 million cells at >98% viability were suspended in 25.5 ml media with 100 µM biotin. Expifectamine (81 µl) and plasmid DNA (30 µg total) were mixed separately with 1.5 ml OptiMEM each, incubated at room temperature for 5 minutes, combined, and incubated for 20 minutes. The mixture was added to the cells, and after 20 hours of growth Enhancers 1 and 2 (150 µl and 1.5 ml, respectively) were added. Proteins were allowed to express for 5 days.

Protein purification.

Expi293 cells were spun down at 3000 × g for 20 minutes, and the supernatant from each culture containing protein of interest was collected, filtered through a 0.22 µM syringe filter and neutralized with 10X phosphate buffered saline (PBS, 0.01 M phosphate buffer, 0.0027 M KCl and 0.137 M NaCl, Millipore Sigma P4417-100TAB), pH 7.4. The supernatants were purified using a peristaltic pump and a HiTrap Protein A column (GE Healthcare). Fc-fused proteins were acid-eluted into 1X PBS, pH 7.4, from protein A columns, and then buffer-exchanged into 1X PBS, pH 7.4, using spin concentration columns (Millipore Sigma). Later ACE2(740)-Fc constructs were eluted with 50 mM Tris pH 7.2, 4 M $MgCl_2$ and similarly buffer exchanged. Biotinylation was quantified by denaturing proteins at 0.1 mg/ml in Lamelli buffer with 5 mM DTT for 5 minutes at 95 °C, followed by addition of a molar excess of avidin and SDS-PAGE. Proteins were >95% pure and >95% biotinylated as determined by ImageJ analysis.

Library preparation.

ACE2 H34V, N90Q, H34V/N90Q, and K31F/H34I/E35Q were cloned into pCL2 as N-terminal fusions to Aga2p followed by eGFP. A silent BamHI site was incorporated at G104/S105, and the BamHI site at the C-terminal linker in pCL2 was deleted for downstream library generation. 1 ng was used as template for initial error-prone PCR using 8-oxo-GTP and dPTP (TriLink Biotechnologies). Four 50 µl PCR

reactions were performed on each template with increasing concentrations of mutagenic nucleotides (5 μM, 10 μM, 50 μM, and 100 μM). The 5 and 10 μM reactions were carried out using Taq polymerase in standard buffer under the following conditions: Initial denaturation at 95 °C for 30 seconds, 20 cycles of PCR (95 °C for 20 seconds, 55 °C for 20 sec, 68 °C for 45 sec), and final elongation at 68 °C for 300 sec. These reactions were loaded onto a 2% agarose gel, and the resulting bands at 330 bp were excised and purified. The 50 and 100 μM mutagenic PCRs were carried out similarly except with 5 cycles of PCR followed by DpnI digestion of the template for several hours at 37 °C. After heat inactivation of the DpnI, 5 μl of crude PCR reaction mixture were used as template for a standard Phusion PCR and gel purified as above. To generate enough DNA for yeast transformation 150 ng from each of these 16 PCRs were used as templates for Phusion PCRs (2 × 50 μl reactions each). After spot-checking several reactions for the expected products by agarose gel, these reactions were pooled into 4 tubes and ethanol precipitated with 0.1 volumes of 3 M sodium acetate pH 5.2, 0.1 μg glycogen, and 3 volumes of ethanol. These were incubated several hours at room temperature followed by overnight at -80 °C and centrifuged for 20 minutes at 16,000 × g. Pellets were washed with cold 70% ethanol and centrifuged again. After removing the supernatant, the pellets were air-dried and suspended in 20 μl of sterile water and centrifuged again.

Yeast transformations.

Briefly, a stationary phase culture of EBY100 was subcultured to an OD600 of 0.3 in 200 ml YPD media and grown at 30 °C with shaking at 250 rpm for 4.5 hours. Upon reaching OD600 = 1.6, cells were centrifuged at 3000 × g for 3 minutes, washed twice with 100 ml ice-cold MilliQ water and once with 100 ml ice-cold electroporation buffer (1 M sorbitol, 1 mM $CaCl_2$). Cells were resuspended in 50 ml 0.1 M lithium acetate/0.01 M DTT and incubated at 30 °C for 30 minutes with shaking at 250 rpm. Cells were pelleted and washed once more with 100 ml electroporation buffer and resuspended in a minimal volume of electroporation buffer. Sublibraries prepared above were pooled into four separate electroporation cuvettes by parental ACE2 variant. A total of 30 μg of each pool was mixed with 10 μg pCL2-ACE2(18-614)BamHI-Aga2-sfGFP previously digested with NheI-HF and BamHI-HF and mixed with 400 μl electrocompetent EBY100 and incubated on ice for 5 minutes. Cells were electroporated using a Biorad Gene Pulser Xcell with an exponential pulse (2.5 kV and 25 μF), pooled, and recovered in 40 ml of 1:1 YPD:electroporation buffer at 30 °C with shaking at 250 rpm. After 2 hours the cells were centrifuged and resuspended in SDCAA and diluted up to 500 ml in SDCAA. Serial dilutions starting at 1/100 were plated on SDCAA agar plates. After 4 days at 30 °C, 56 colonies were counted on a 1/100 dilution plate for a total library size of 2.8×10^7.

Analysis of yeast library.

5-10 ml of saturated SDCAA culture were pelleted and resuspended in 200 μl buffer P1. 10 μl of Zymolyase (Zymo Research E1004) or 50 μl Lyticase from *Arthrobacter luteus* (Sigma L4025-25KU) were added and the cells were incubated at 37 °C for 1-2 hours. An equal volume of buffer P2 was added and cells were incubated 10 minutes at RT with gentle mixing. 350 μl buffer N3 was added and the lysate was centrifuged at 16,200 × g for 10 minutes. The supernatant was applied to an EconoSpin miniprep column, washed with 500 μl of buffer PB followed by 750 μl buffer PE, and eluted in 50 μl buffer EB. Library pools were transformed into XL10-Gold competent cells and plated for individual colonies on LB + 50 μg/ml carbenicillin agar plates, while individual clones were amplified directly by PCR.

Library screening.

Cells were sorted using a BD FACS Aria II. For sort 1, approximately 10^8 induced library cells were washed twice with PBSA and stained with 50 nM biotinylated RBD monomer (10 ml) for 1 hour at room temperature. After washing three times with 5 ml ice-cold PBSA, cells were incubated on ice with a 1/1000 dilution of streptavidin Alexa Fluor 647 conjugate. Cells were washed twice with 10 ml PBSA

and immediately used to sort binding clones. For sort 1, the top 5% of RBD binders from 5×10^7 cells were sorted into 3 ml SDCAA. These were pelleted at 3000 × g for 3 minutes and used to inoculate 50 ml in SDCAA. After overnight growth at 30 °C, cells were spun down and used to start a 50 ml, OD = 1 culture in SGCAA. For sort 2, 10^7 cells were washed and stained with 10 ml of 5 nM biotinylated RBD monomer and processed similarly to round 1. The top 0.25% of RBD binders were sorted into 3 ml SDCAA, diluted to a 5 ml culture in SDCAA, and grown at 30 °C overnight. For sorts 3.1 and 3.2, cells from sort 2 were subcultured (starting OD = 1 in SGCAA) and induced at 20 °C overnight. 10^7 cells were washed with PBSA stained with 500 pM or 200 pM RBD monomer (80 ml) for 4 hours at RT followed by washing and secondary staining as above. Approximately 6×10^6 cells were analyzed and the top 1% were collected and cultured in 20 ml SDCAA. For sort 4, cells from sort 3 were subcultured as above. 5×10^6 cells were washed in PBSA and stained with 5 ml 5 nM RBD monomer for 30 minutes at RT, followed by 3 washes with PBSA. Cells were resuspended in 10 ml PBSA with 20 nM H34V-ACE2(614)-Fc and incubated at RT for 8 hours with rotation. Cells were washed once and stained with 2 ml 1/1000 streptavidin Alexa Fluor 647 for 20 minutes, washed twice with PBSA and resuspended in 1 ml PBSA. The top 1% of cells were sorted and cultured in 20 ml SDCAA. 100 µl of a 1/100 dilution of the culture was plated on SDCAA agar to isolated individual clones for analysis. Sort 5 was performed similarly to sort 4 but with a 12-hour dissociation with 20 nM soluble H34V-ACE2(614)-Fc as a competitor. The top 0.2% of cells were collected and cultured as above.

Flow cytometry.

Flow cytometry analysis of individual ACE2-expressing yeast clones was carried out using a Beckman Coulter Cytoflex flow cytometer. Approximately 50,000 cells from an overnight SGCAA culture were pelleted by centrifugation at 3,000 × g for 3 minutes and washed in HyClone PBS + 3% BSA (PBSA). Cells were resuspended in 100 µl PBSA, and appropriate volumes of biotinylated RBD monomer were added to avoid ligand depletion (estimating 50,000 copies of ACE2 per cell). These were incubated 2-4 hours at room temperature with rotation to reach equilibrium, washed three times with PBSA, and stained for 20 minutes on ice with 1 µg/ml Alexa Fluor 647-conjugated streptavidin (Thermofisher Scientific S21374). After two more washes with HyClone PBS (without BSA) cells were suspended in 200 µl and analyzed. To fit yeast binding data to K_D values, Alexa Fluor 647 mean fluorescence intensities were extracted from the GFP-positive population, background subtracted using secondary only controls, normalized to the highest fluorescent population, and fit to the Hill equation without cooperativity in Python.

Supplemental tables

Table S1. Computational alanine scanning results using established protocols (3, 4)**.** Columns contain: pdb#: PDB residue number; chain: PDB chain ID (chain A is ACE2, chain B is the spike RBD); int_id: equal to 1 if at least one atom in the residue is within 4 Å of an atom on the other chain, and 0 otherwise; aa: amino acid type; DDG(complex): predicted change in binding energy upon alanine mutation; DG(partner): predicted change in stability of the mutated complex partner upon alanine mutation; DMS beneficial mutations: for ACE2, beneficial point mutations predicted in Procko (5).

pdb#	chain	int_id	aa	DDG(complex)	DG(partner)	DMS beneficial mutations
417	B	1	LYS	0.21	0.78	
449	B	1	TYR	1.44	1.25	
453	B	1	TYR	1.37	3.11	
455	B	1	LEU	1	1.6	
456	B	1	PHE	1.87	1.61	
486	B	1	PHE	2.31	-0.6	
487	B	1	ASN	1.71	0.64	
489	B	1	TYR	1.86	1.56	
493	B	1	GLN	1.63	-0.29	
494	B	0	SER	-0.02	0.21	
498	B	1	GLN	1.31	1.4	
500	B	1	THR	-0.03	0.3	
501	B	1	ASN	0.22	1.61	
503	B	0	VAL	0.03	-0.1	
505	B	1	TYR	2.09	0.57	
19	A	1	SER	0.71	-0.42	V, W, Y, F, P
24	A	1	GLN	0.59	1.01	T
27	A	1	THR	0.73	-0.06	M, L, A, D, K H, W, Y, F, C
28	A	1	PHE	0.17	3.88	none
30	A	1	ASP	0.14	-0.98	I, V, E
31	A	1	LYS	0.61	-0.47	W, Y
34	A	1	HIS	1.86	-0.59	V, A, S, P
35	A	1	GLU	0.66	-0.24	M, V, D, C
38	A	1	ASP	0.35	-0.67	none
41	A	1	TYR	2.39	3.13	R
42	A	1	GLN	2.71	-0.59	M, L, I, V, K, R, H, C
45	A	0	LEU	0.26	0.93	none
79	A	1	LEU	0.6	0.93	M, I, V, T, R, W, Y, F, P
82	A	1	MET	0.5	0.4	R, G, C
83	A	1	TYR	2.21	3.79	none
351	A	0	LEU	0.02	3.19	F
353	A	1	LYS	1.23	1.01	none
355	A	1	ASP	3.23	2.22	none

Table S2. Computational saturation mutagenesis at ACE2 positions 34, 42 and 353 scored using Rosetta total score and interface energies. Columns contain the following information: Mutation: single-letter amino acid identifier to which original sidechain was mutated: Total energy: Rosetta score for the complex (in REU); Interface: the lowest calculated interface energy of five trials (in REU); delta: the difference between the interface energy of the wild-type complex and the interface energy of the point mutant (in REU). Interface energies lower than those determined for the WT complex are highlighted in yellow (see "Computational saturation mutagenesis at targeted ACE2 interface residue positions," Methods).

	H34			Q42			K353		
**mutation**	Total energy	Interface	delta	Total energy	Interface	delta	Total energy	Interface	delta
**A**	-2079.41	-58.1059	-0.974	-2075.896	-53.869	3.263	-2074.771	-54.8121	2.320
**R**	-2075.486	-55.5158	1.617	-2079.445	-55.8825	1.250	-2073.901	-52.8174	4.315
**N**	-2077.512	-58.2728	-1.141	-2077.357	-55.039	2.093	-2074.215	-57.2794	-0.147
**D**	-2072.993	-55.7567	1.376	-2075.277	-54.0632	3.069	-2067.808	-54.8079	2.324
**Q**	-2077.21	-58.9142	-1.782				-2073.781	-54.8442	2.288
**E**	-2076.387	-56.5619	0.570	-2078.102	-53.8338	3.299	-2068.393	-50.321	6.811
**G**	-2075.524	-56.1769	0.955	-2074.099	-53.8018	3.331	-2070.87	-51.5659	5.566
**H**				-2077.636	-54.026	3.106	-2071.823	-56.1115	1.021
**I**	-2068.827	-53.3366	3.796	-2078.778	-54.1272	3.005	-2064.506	-52.8856	4.247
**L**	-2072.173	-57.2587	-0.126	-2079.427	-55.135	1.997	-2075.772	-54.933	2.199
**K**	-2075.855	-54.9393	2.193	-2079.221	-56.2299	0.902			
**M**	-2076.286	-58.3653	-1.233	-2078.496	-53.8281	3.304	-2073.167	-57.2724	-0.140
**F**	-2077.452	-58.6328	-1.501	-2076.563	-53.973	3.159	-2075.907	-54.8568	2.276
**P**	-2062.583	-56.1616	0.971	-2065.709	-52.2274	4.905	-1984.256	-53.6565	3.476
**S**	-2076.239	-55.6296	1.503	-2076.745	-53.8748	3.258	-2069.483	-56.5399	0.592
**T**	-2075.284	-58.5258	-1.394	-2076.591	-54.0055	3.127	-2067.071	-55.8814	1.251
**W**	-2076.567	-58.0243	-0.892	-2077.569	-54.8777	2.255	-2068.537	-55.7901	1.342
**Y**	-2077.384	-58.5473	-1.415	-2075.443	-54.2037	2.929	-2073.076	-56.874	0.258
**V**	-2078.676	-59.8262	-2.694	-2077.3	-53.9666	3.166	-2066.138	-55.3603	1.772
**WT**	-2048.31	-57.1323	0.000	-2048.31	-57.1323	0.000	-2048.31	-57.1323	0.000

Table S3. Apparent binding affinities of ACE2(614) variants measured on the surface of yeast with monomeric SARS-CoV-2 spike RBD. $K_{D,app}$ values reported as the average from the fit to all data in duplicate experiments, as shown in Figure 3D-E, with the errors of the fit. Aga2p-GFP constructs were used in all yeast surface display experiments (6). These are listed alongside the ACE2(614)-Fc and ACE2(740)-Fc constructs that include the same affinity-enhancing mutations for convenience.

**Mutations**	**Origin**	**$K_{D,app}$ (nM) (on yeast)**	**Aga2p-GFP construct**	**ACE2(614)-Fc construct**	**ACE2(740)-Fc construct**
-	WT	20.4 ± 1.8	Y208	CVD013	CVD208
H34V	Computational design	9.29 ± 0.78	Y295	CVD014, CVD127*	CVD295
N90Q	(5)	4.54 ± 0.55	Y117	CVD117	
K31F, H34I, E35Q	Computational design	1.71 ± 0.02	Y293	CVD019	CVD293
H34V, N90Q	Computational design	5.47 ± 0.68	Y292	CVD118*, CVD278†	CVD292
A25V, T27Y, H34A, F40D	DMS-guided design	0.40 ± 0.03	Y310		CVD310†
K31Y, W69V, L79T, L91P	DMS-guided design	0.64 ± 0.05	Y311		CVD311†
T27Y, H34A, N90Q	DMS-guided design	0.84 ± 0.09	Y312		CVD312†
S19P, Q42L, L79T, N90Q	DMS-guided design	0.94 ± 0.09	Y355		CVD355†
K31F, N33D, H34S, E35Q	Yeast display, round 3.1	0.52 ± 0.04	Y313		CVD313†
K31F, N33D, H34A, E35Q, N49D, N51S, N53S, E57G, N64D	Yeast display, round 3.2	0.45 ± 0.18	Y354		CVD354†
T27A, K31F, N33D, H34S, E35Q, N61D, K68R, L79P	Yeast display, round 4	0.19 ± 0.02	Y353		CVD353†
S19P, N33S, H34V, F40L, N49D, L100P	Yeast display, round 4	0.61 ± 0.04	Y375		CVD375†
Q18R, K31F, N33D, H34S, E35Q, W69R, Q76R	Yeast display, round 5	0.12 ± 0.01	Y373		CVD373†

* includes H374N/H378N inactivation mutations
† includes H345L inactivation mutation

Table S4. Half-maximal inhibitory concentrations (IC50s) of ACE2 variants. IC50 values were calculated from titration experiments in pseudotyped lentiviral SARS-CoV-2 neutralization assays and authentic SARS-CoV-2 neutralization assays in a biosafety level 3 facility with VeroE6 cells. IC50 values reported as means. Errors reported for pseudovirus experiments are standard deviations between 4 and 12 technical replicates. Errors reported for authentic SARS-CoV-2 experiments represent the error of the fit for biological replicates.

ID	Mutations	Scaffold	IC50 (μg/ml), pseudovirus	IC50 (μg/ml), VeroE6
CVD013	-	ACE2(614)-Fc	0.43 ± 0.39	
CVD208	-	ACE2(740)-Fc	0.71 ± 0.51	
CVD208e	-	ACE2(740)	2.19 ± 1.15	
CVD014	H34V	ACE2(614)-Fc	0.35 ± 0.19	
CVD019	K31F, H34I, E35Q	ACE2(614)-Fc	0.31 ± 0.16	
CVD118	H34V, N90Q, H374N†, H378N†	ACE2(614)-Fc		< 0.5
CVD293	K31F, H34I, E35Q	ACE2(740)-Fc	0.036 ± 0.01	0.136 ± 0.08
CVD310	A25V, T27Y, H34A, F40D, H345L†	ACE2(740)-Fc	0.058 ± 0.03	0.089 ± 0.01
CVD311	K31Y, W69V, L79T, L91P, H345L†	ACE2(740)-Fc	0.055 ± 0.03	
CVD313	K31F, N33D, H34S,E35Q, H345L†	ACE2(740)-Fc	0.028 ± 0.02	0.073 ± 0.02
CVD353	T27A, K31F, N33D, H34S,E35Q, N61D, K68R, L79P, H345L†	ACE2(740)-Fc	0.69 ± 0.38	

† mutation inactivating the ACE2 enzymatic function

Supplemental figures

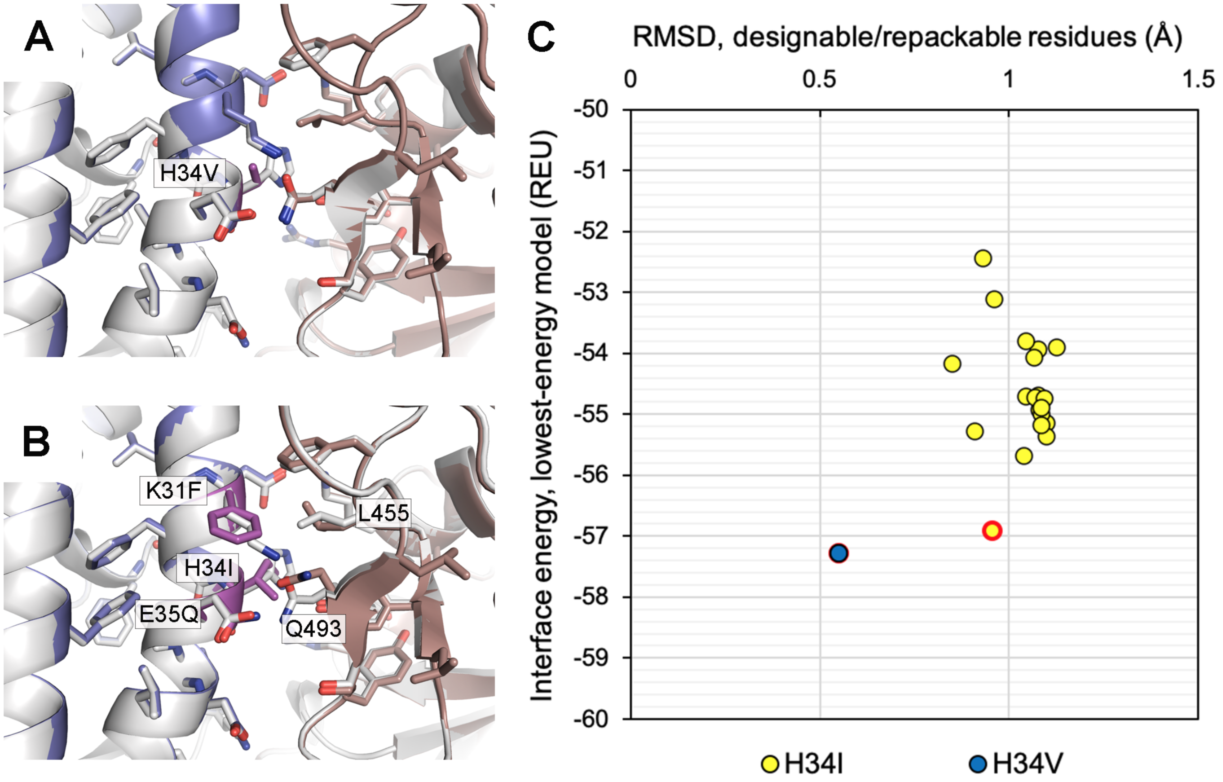

Figure S1. Comparison between models of redesigned ACE2-SARS-CoV2 spike RBD interfaces in ACE2 H34V and H34I backgrounds. Lowest-energy models for redesigned ACE2 in the **(A)** H34V and **(B)** H34I mutation backgrounds. The wild-type ACE2-RBD interface is shown in white. Redesigned ACE2 is shown in blue. Repacked RBD is shown in dark salmon. Mutated residues in the redesigned ACE2 are shown in magenta. Sidechains with any atoms within 6 Å of position 34 are shown as sticks. Mutated ACE2 residues and RBD residues that adopted different rotameric conformations are labeled. **(C)** Root-mean-square deviation (RMSD) from WT ACE2 for all backbone and sidechain heavy atoms belonging to mutable and redesignable residues vs. summed pairwise interface energies for lowest energy solutions for all trials of ACE2-spike RBD interface design in H34V and H34I backgrounds. All the solutions for the H34V models have very similar energies and RMSD, but solutions in the H34I background are diverse. Red-circled points are the lowest-energy solutions depicted in **(A)** and **(B)**. Models based on PDB 6LZG (7).

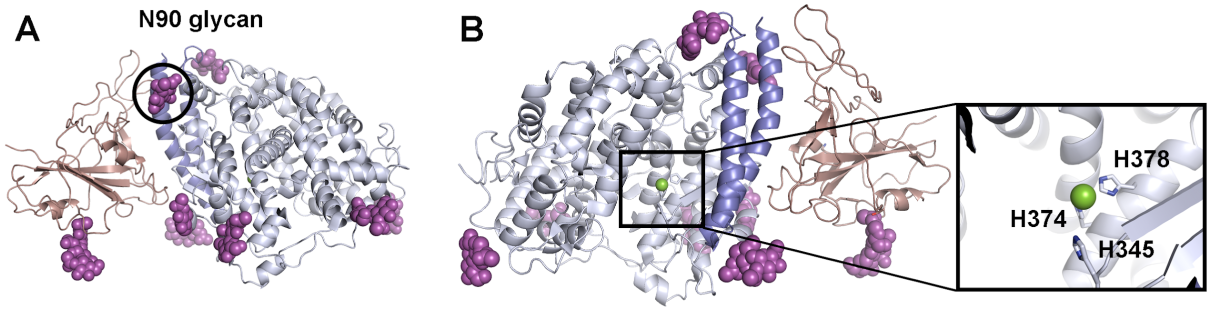

Figure S2. Additional ACE2 mutations for enzyme inactivation and improved affinity to the spike RBD. (A) Procko (5) showed that mutants in which the N90 glycan (circled) adjacent to the ACE2-RBD interface was knocked out were enriched in DMS selection experiments. **(B)** The active site of ACE2 (square region) binds a Zn^{2+} ion, which is coordinated by H378 and H374 (8). H345, also shown, is important for substrate binding in catalysis (9). The spike RBD is shown in dark salmon, ACE2 is shown in light blue with N-terminal helices (residues 18-90) shown in dark blue, glycans are shown in magenta, and the Zn^{2+} ion is shown in green. Structures are from PDB 6M17 (10).

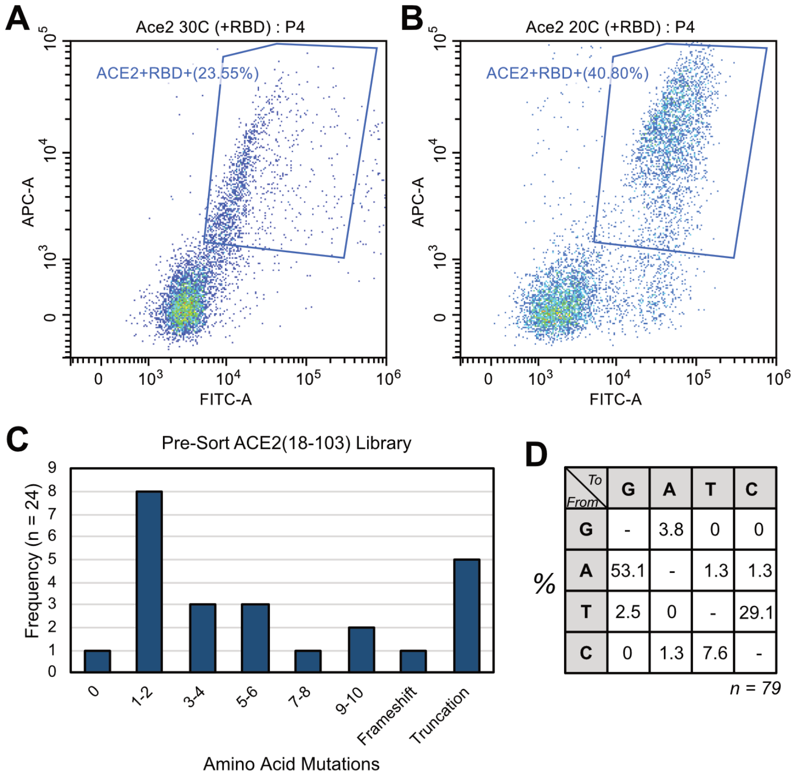

From \ To	G	A	T	C
G	-	3.8	0	0
A	53.1	-	1.3	1.3
T	2.5	0	-	29.1
C	0	1.3	7.6	-

Figure S3. Yeast surface display of ACE2(614) and library analysis. WT ACE2(614) was expressed as an Aga2p fusion at **(A)** 30 °C and **(B)** 20 °C and bound to 100 nM biotinylated Spike-RBD-Fc followed by streptavidin Alexa Fluor 647. In **(A)** and **(B)**, the FITC-A axis represents protein expression, and the APC-A axis represents binding to the RBD. **(C)** Analysis of 24 clones of ACE2(614) mutagenized at amino acids 18-103 by error-prone PCR showed a broad distribution of mutations per clone. **(D)** 79 total DNA mutations from these 24 clones had mutational bias expected from dNTP analog epPCR.

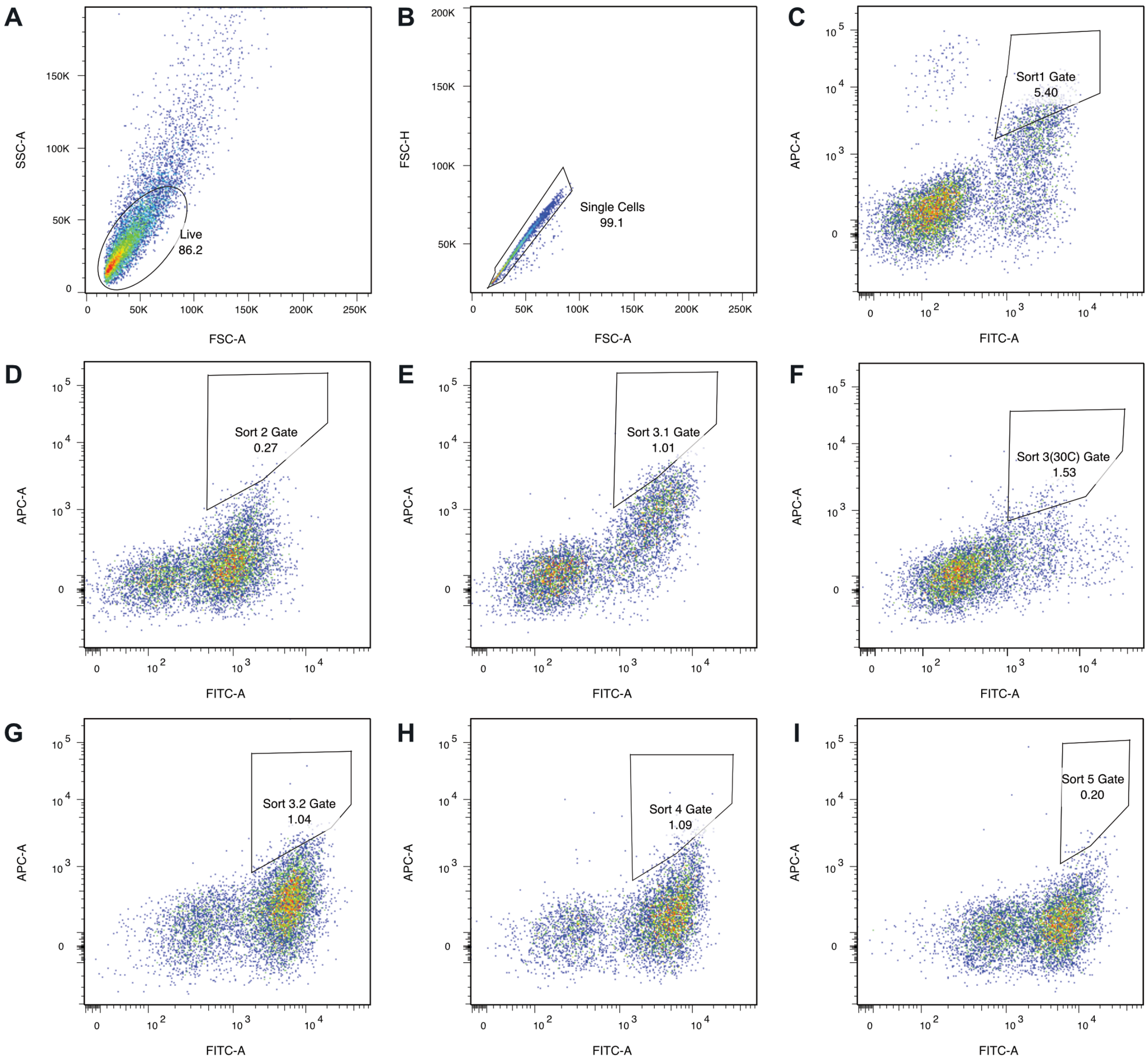

Figure S4. ACE2(614) affinity maturation sort gates. Each sample was gated on **(A)** live cells and **(B)** singlets. Approximate sort gates are shown for **(C)** Sort 1 (50 nM RBD monomer), **(D)** Sort 2 (5 nM RBD monomer), **(E)** Sort 3.1 (0.5 nM RBD monomer), **(F)** Sort 3 (0.5 nM RBD monomer, 30 °C expression), **(G)** Sort 3.2 (0.2 nM RBD monomer), **(H)** Sort 4 (8 hour dissociation with 20 nM soluble H34V-ACE2(614)-Fc competitor), and **(I)** Sort 5 (12 hour dissociation with 20 nM soluble H34V-ACE2(614)-Fc competitor).

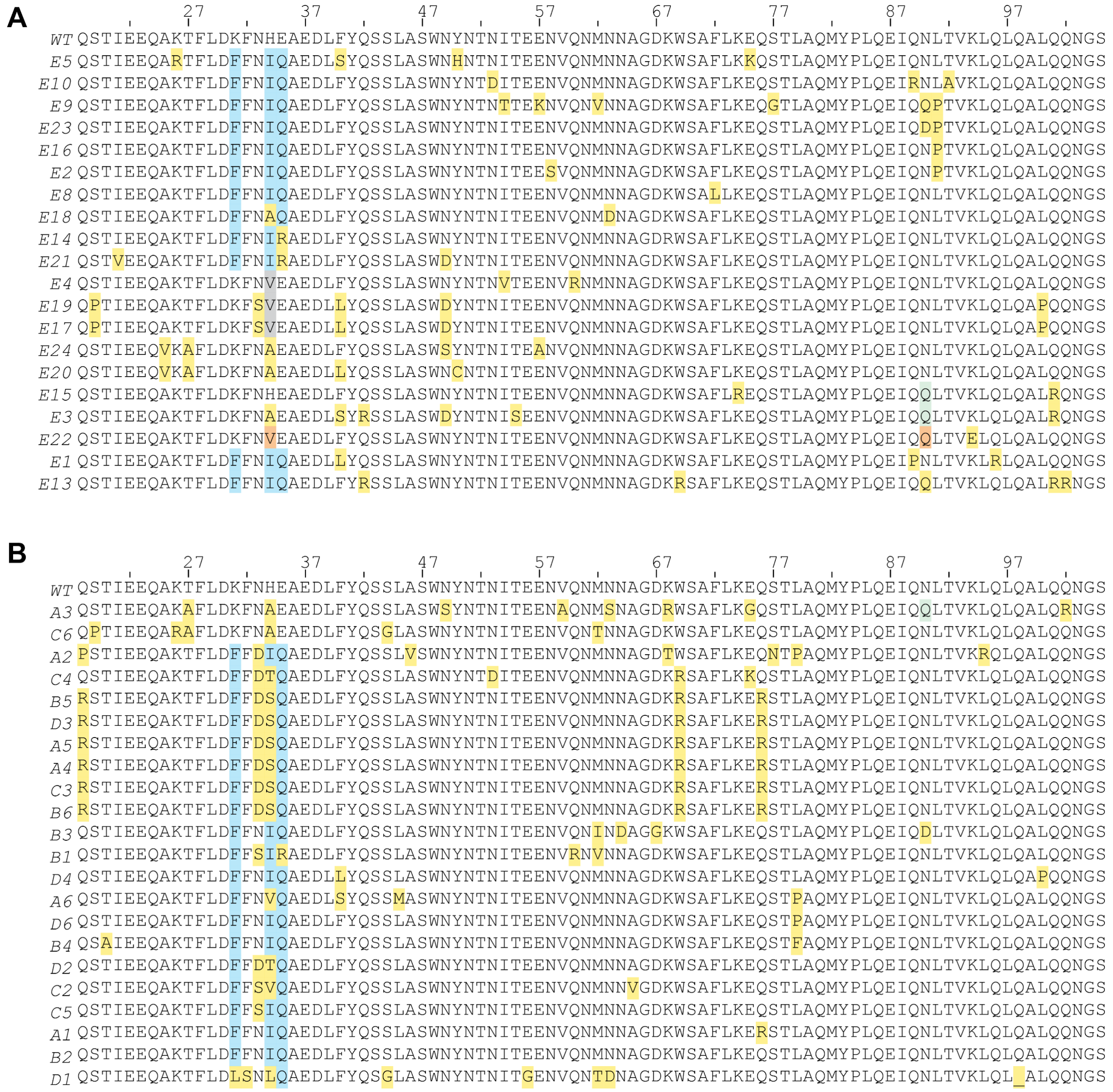

Figure S5. Sorts 4-5 ACE2 sequence alignments. (A) Sanger sequencing of individual clones from Sort 4 showed no convergence. **(B)** Sort 5 was enriched for Q18R/K31F/N33D/H34S/E35Q/W69R/Q76R ACE2(614). The colors are as follows: yellow, mutations from error-prone PCR; blue, mutation most likely from K31F/H34I/E35Q parent; gray, mutation most likely from H34V parent; green, mutation most likely from N90Q parent; brown, mutation most likely from H34V/N90Q parent.

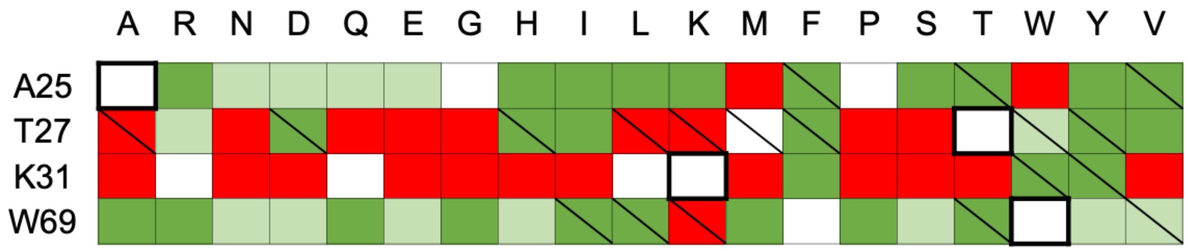

Figure S6. Computational saturation mutagenesis on additional positions in the ACE2-spike RBD interface. Interface energies for the whole ACE2-spike RBD interface were calculated on the lowest total energy models from computational saturation mutagenesis at several positions that were not alanine-scanning hotspots. Boxes are colored according to the interface energy difference of the point mutant model with the WT ACE2-RBD: red, DDG > 0.5 REU; white, DDG ≤ 0.5 REU and ≥ -0.6 REU; light green, DDG < -0.6 REU and ≥ -0.9 REU; dark green, DDG < -0.9 REU. Thick lines around boxes indicate the WT amino acid. Boxes with diagonal lines indicate amino acid substitutions identified as beneficial by DMS (5).

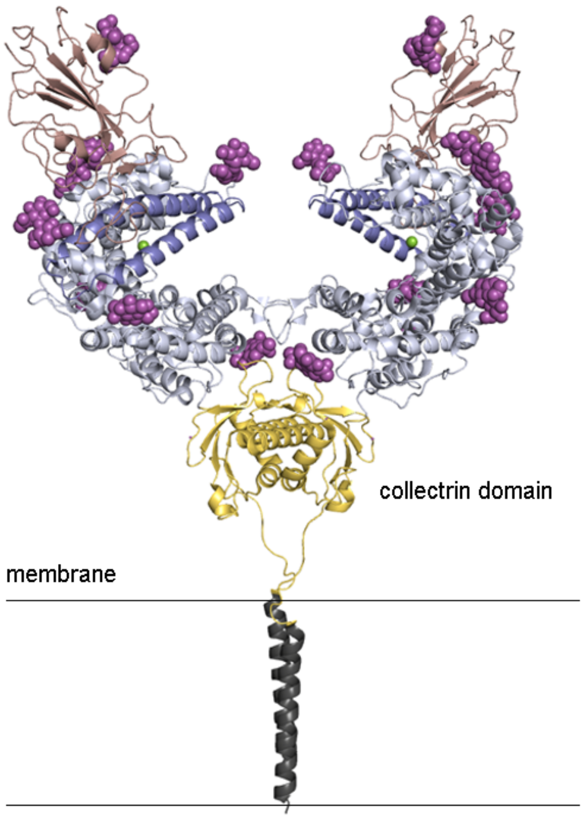

Figure S7. ACE2 residues 615-740 form a collectrin domain. In ACE2, the collectrin domain (yellow) connects the transmembrane helices (dark gray) to the soluble extracellular peptidase domain (residues 18-90 in blue, residues 91-614 in light blue). The SARS-CoV-2 spike RBD is shown in dark salmon. The Zn^{2+} ion is shown in green. All glycans are shown in magenta. PDB 6M17 (10).

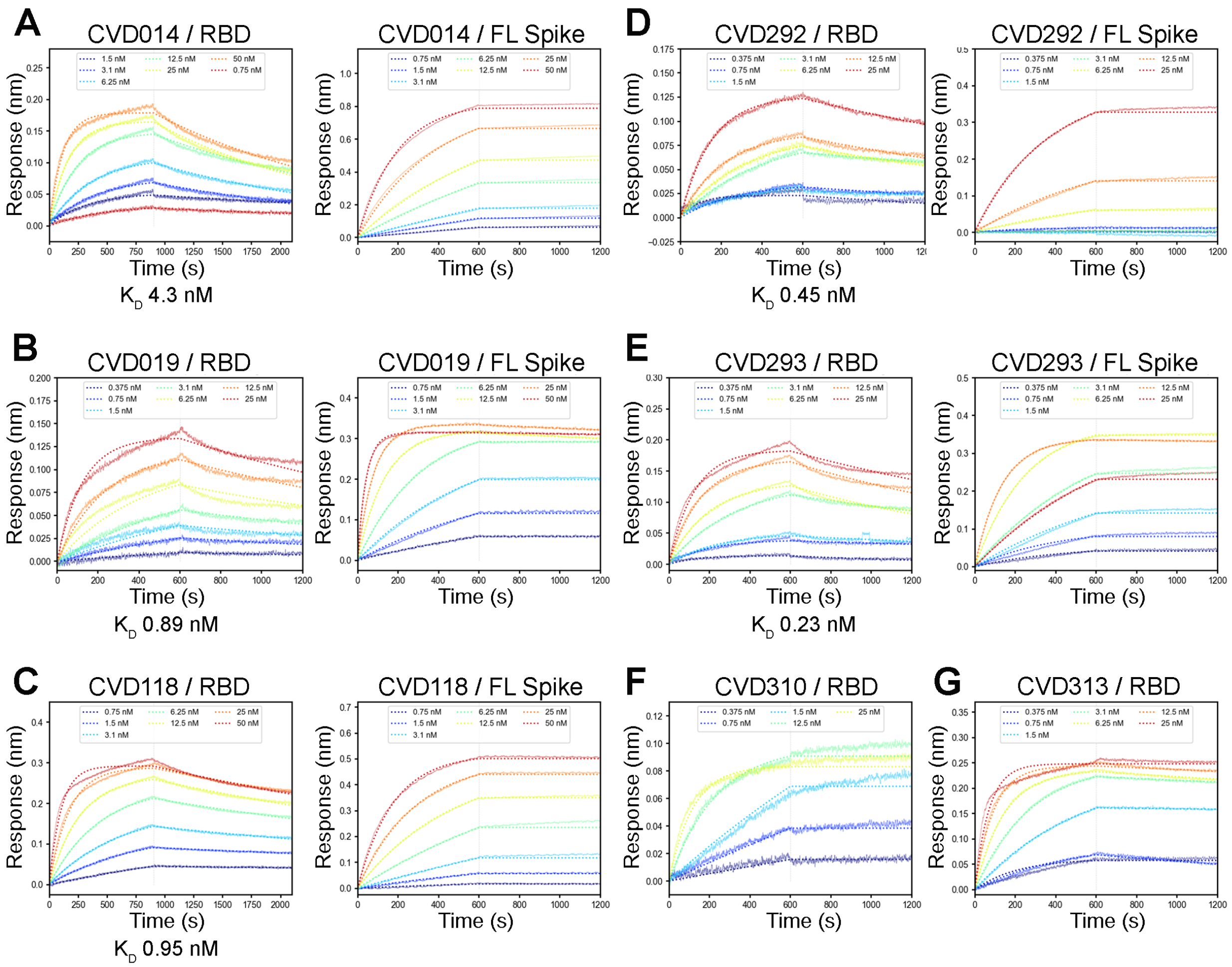

Figure S8. ACE2-Fc variants with and without collectrin domain bind full-length (FL) spike more tightly than spike RBD. Representative BLI measurements show that designed **(A-C)** ACE2(614)-Fc variants and **(D-E)** ACE2(740)-Fc variants have a higher binding affinity for FL spike (right) as compared to the monomeric spike RBD (left). K_D for all ACE2 variants binding to RBD are reported. Due to decreased off-rates, K_D for FL spike could not be calculated. **(F-G)** ACE2(740)-Fc variants from DMS-guided design and affinity maturation in yeast bind the spike RBD with decreased off-rates, precluding accurate estimation of binding affinity from BLI experiments.

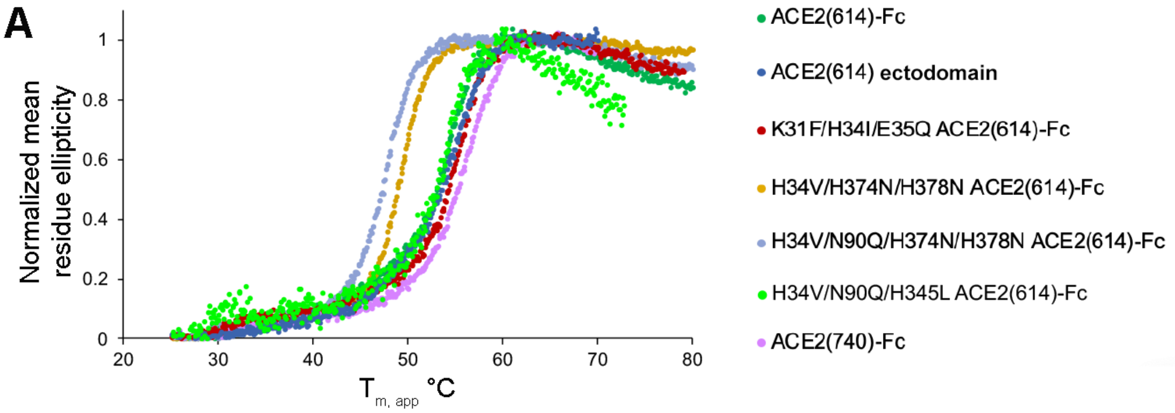

CVD118	CVD127	CVD013	CVD278	CVD013e	CVD019	CVD208
ACE2(18-614)-Fc H34V, N90Q, H374N, H378N	ACE2(18-614)-Fc H34V, H374N, H378N	ACE2(18-614)-Fc	ACE2(18-614)-Fc H34V, N90Q, H345L	ACE2(18-614) ectodomain	ACE2(18-614)-Fc K31F, H34I, E35Q	ACE2(18-740)-Fc
47.3 °C	48.9 °C	51.8 °C	52.7 °C	53.1 °C	54.1 °C	55.3 °C

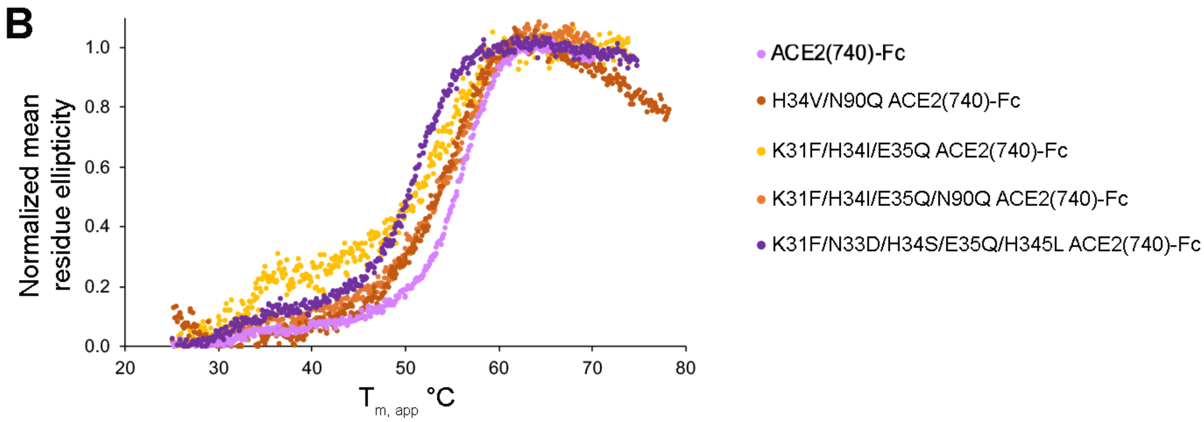

CVD293	CVD313	CVD294	CVD292	CVD208
ACE2(18-740)-Fc K31F, H34I, E35Q	ACE2(18-740)-Fc K31F, N33D, H34S, E35Q, H345L	ACE2(18-740)-Fc K31F, H34I, E35Q, N90Q	ACE2(18-740)-Fc H34V, N90Q	ACE2(18-740)-Fc
50.0 °C	50.2 °C	52.5 °C	52.6 °C	55.3 °C

Figure S9. Thermostability of ACE2-Fc constructs measured by circular dichroism spectroscopy (CD). (A) CD melt curves show that affinity-enhancing mutations do not greatly reduce protein stability, but that mutations to residues coordinating the Zn^{2+} ion (H374N, H378N) do destabilize the ACE2-Fc scaffold. Catalytic inactivation by the H345L mutation is not destabilizing and has similar affinity to the WT ACE2-Fc (Figure S10). Inclusion of the collectrin domain, ACE2 residues 615-740, further enhances stability. **(B)** CD melt curves show slight destabilization of ACE2(740)-Fc variants with computationally-designed (CVD292, 293, 294 variants) and yeast-selected (CVD313) affinity-enhancing mutations. These variants have apparent melting temperatures ($T_{m,app}$) in the 50-53 °C range.

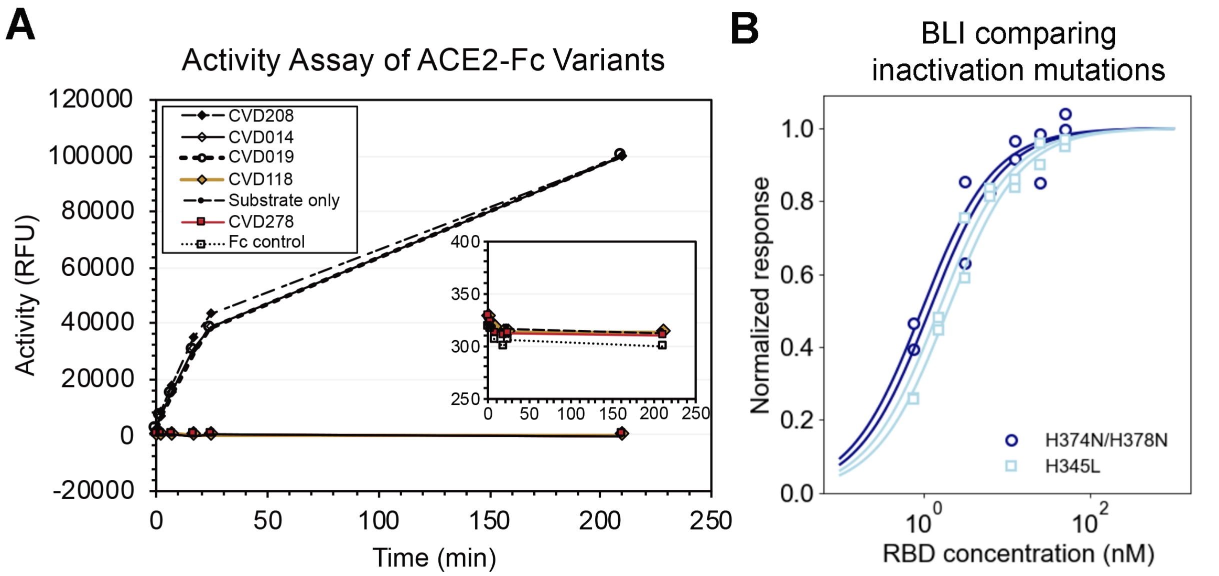

Figure S10. ***In vitro*** **binding and activity characterization of inactivation mutations in ACE2-Fc. (A)** Enzyme activity was assayed by monitoring the increase in fluorescence resulting from hydrolysis of Mca-APK-DNP. CVD014 and 019 had similar activity to wild-type CVD208 but the H374N/H378N mutations (in CVD118) and the H345L mutation (in CVD278) have no detectable catalytic activity. **(B)** BLI data comparing the effect on binding the spike RBD for the different inactivation mutations, H374N/H378N and H345L. Both ACE2-Fc constructs also contain affinity-enhancing mutations H34V and N90Q.

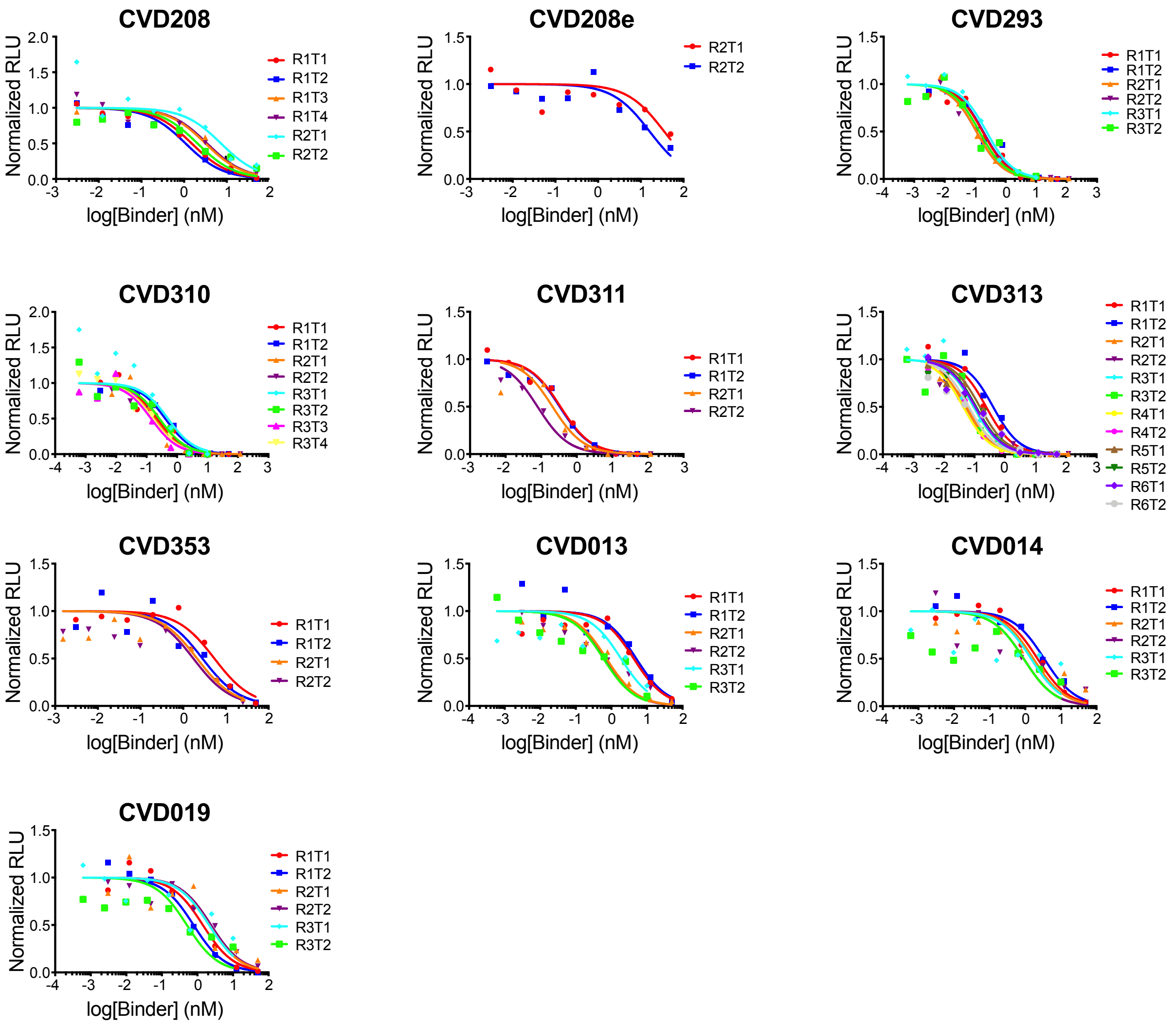

Figure S11. Pseudotyped SARS-CoV-2 neutralization IC50 curves for WT and engineered ACE2(614)-Fc and ACE2(740)-Fc molecules. Normalized luminescent response for each technical replicate is shown as a separate line. Labels indicate biological replicate (R) and technical replicate (T) for each experiment (e.g. R1T1, biological replicate 1 and technical replicate 1).

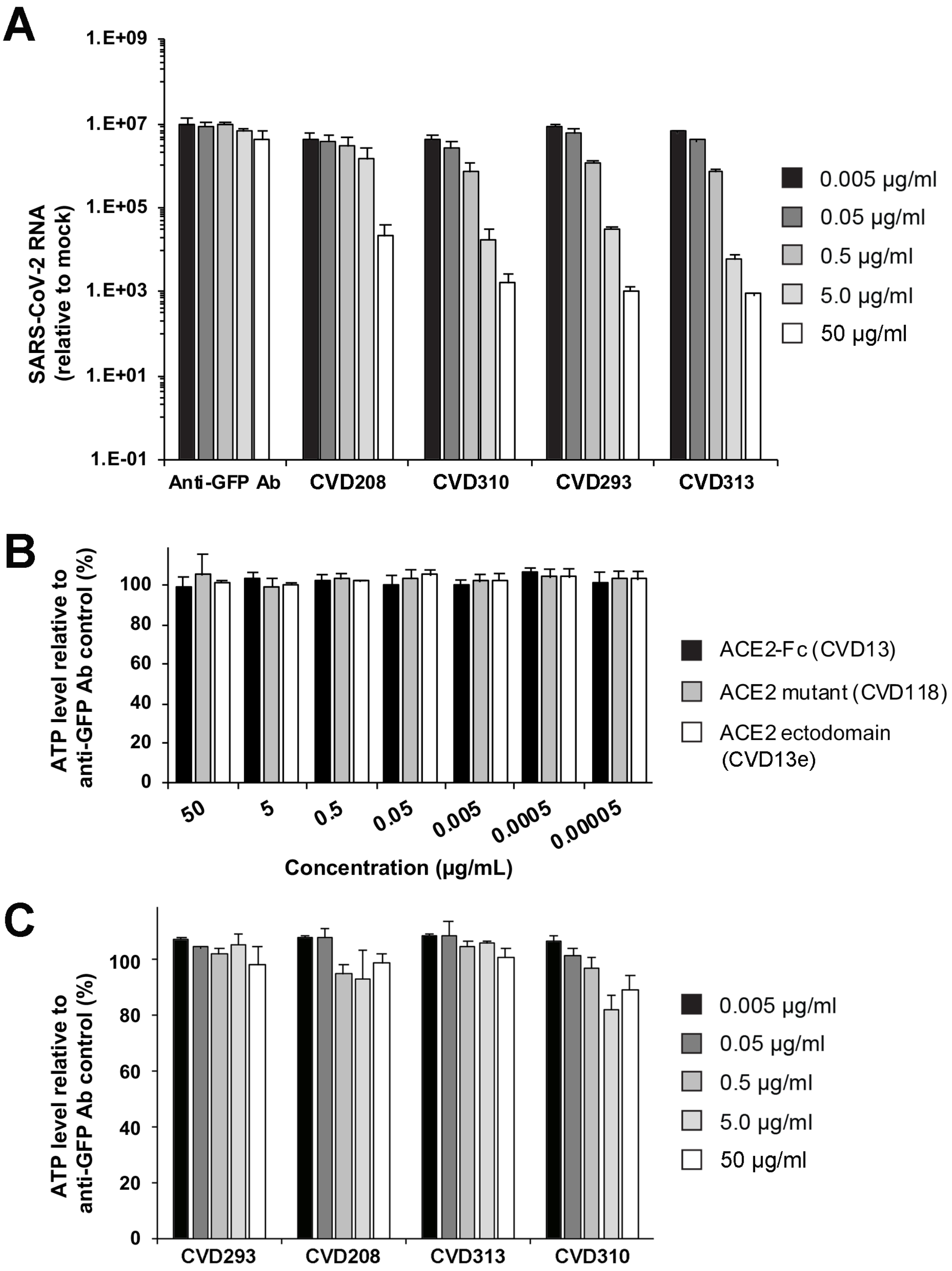

Figure S12. ACE2 variants (A) effectively neutralize SARS-CoV-2 in VeroE6 cells and (B-C) are not cytotoxic in uninfected VeroE6 cells after 24 hours of treatment. Panel **(A)** shows the same data from Fig. 4F without normalization to the anti-GFP antibody control. ATP release was measured by luminescence signal using the CellTiter-Glo assay (Promega) in ACE2 variants-treated cells relative to anti-GFP IgG-treated cells. Error bars represent standard error of the mean for biological duplicates.

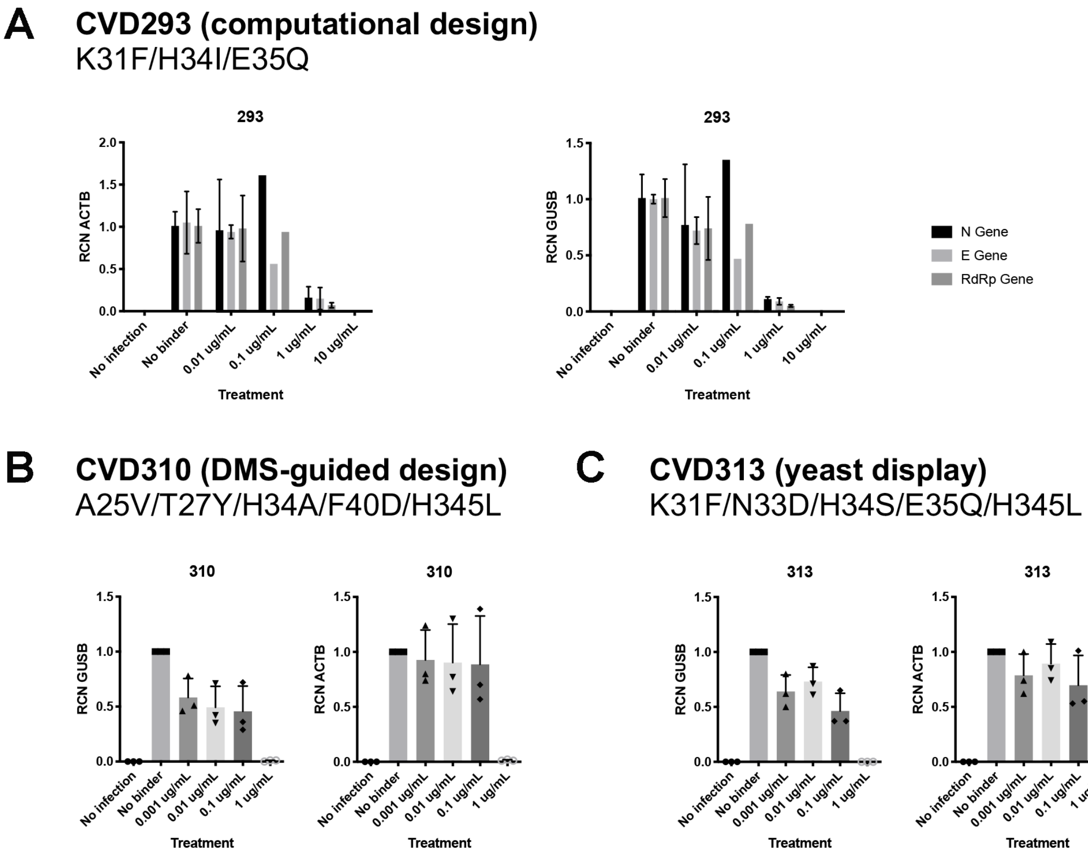

Figure S13. Computationally designed and affinity-matured ACE2(740)-Fc variants effectively block infection of VeroE6 cells by SARS-CoV-2. Live virus qPCR assays of three viral genes (N, E, RdRp) were run 16 hours post-infection using host genes BGUS or ACTB as normalization controls. **(A)** K31F/H34I/E35Q ACE2(740)-Fc (variant 293) has an IC50 between 0.1 and 1 µg/ml. Error bars represent technical duplicates. **(B)** A25V/T27Y/H34A/F40D/H345L ACE2(740)-Fc (variant 310) and **(C)** K31F/N33D/H34S/E35Q/H345L ACE2(740)-Fc (variant 313) neutralize completely at 1 µg/ml. Symbols in **(B)** and **(C)** represent the signal for the individual viral genes.

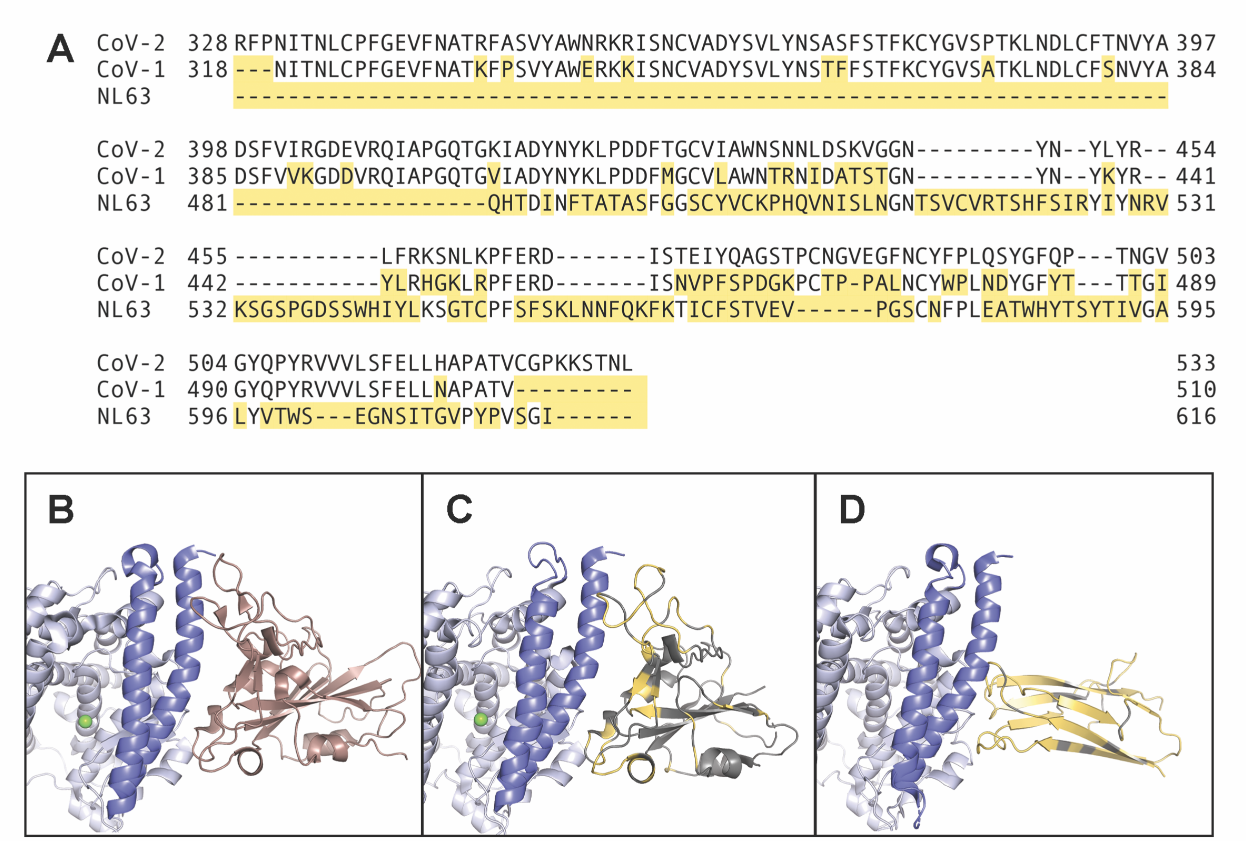

Figure S14. Sequences and structures of receptor binding domains from SARS-CoV-2, SARS-CoV-1, and HCoV-NL63 spike proteins. (A) Sequence alignment for RBDs from all three spike proteins. Yellow residues in the SARS-CoV-1 and HCoV-NL63 RBD sequences (bottom rows) are different from the residue at that position in the SARS-CoV-2 RBD (top row). Numbers flanking each row indicate residue positions for each RBD sequence. **(B)** Structure of the ACE2-SARS-CoV-2 RBD interface (PDB 6LZG) (7). **(C)** Structure of the ACE2-SARS-CoV-1 RBD interface (PDB 2AJF) (11). **(D)** Structure of the ACE2-NL63 RBD interface (PDB 3KBH) (12). In **(B-D)**, ACE2 residues 18-90 are colored dark blue, ACE2 residues 91-614 are colored light blue, and the active site Zn^{2+} ion is colored green. In **(B)**, the RBD is colored dark salmon. In **(C)** and **(D)**, the RBD residues are colored by sequence alignment: residues that are white in **(A)** are colored gray in the structures, and residues that are yellow in **(A)** are colored yellow in the structures.

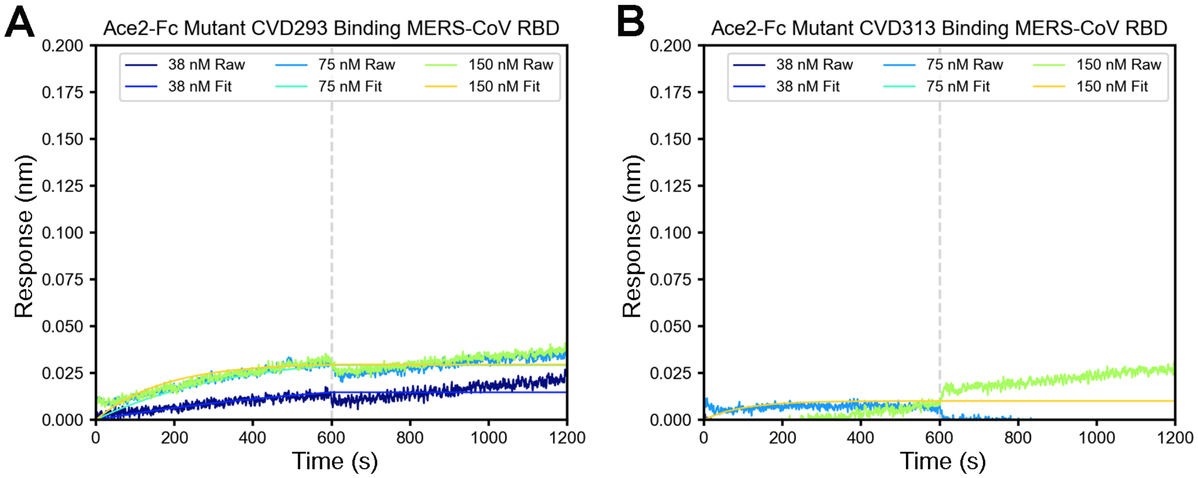

Figure S15. Computationally designed and affinity-matured ACE2(740)-Fc variants do not bind MERS RBD. BLI measurements for **(A)** K31F/H34I/E35Q ACE2(740)-Fc binding MERS-CoV RBD and **(B)** K31F/N33D/H34S/E35Q/H345L ACE2(740)-Fc binding the MERS-CoV RBD.

References

1. S. J. Fleishman, *et al.*, RosettaScripts: A Scripting Language Interface to the Rosetta Macromolecular Modeling Suite. *PLOS ONE* **6**, e20161 (2011).
2. N. Ollikainen, R. M. de Jong, T. Kortemme, Coupling Protein Side-Chain and Backbone Flexibility Improves the Re-design of Protein-Ligand Specificity. *PLoS Comput. Biol.* **11** (2015).
3. T. Kortemme, D. Baker, A simple physical model for binding energy hot spots in protein–protein complexes. *Proc. Natl. Acad. Sci.* **99**, 14116–14121 (2002).
4. T. Kortemme, D. E. Kim, D. Baker, Computational Alanine Scanning of Protein-Protein Interfaces. *Sci. STKE* **2004**, pl2–pl2 (2004).
5. E. Procko, "The sequence of human ACE2 is suboptimal for binding the S spike protein of SARS coronavirus 2" (Biochemistry, 2020) https:/doi.org/10.1101/2020.03.16.994236 (July 14, 2020).
6. S. Lim, J. E. Glasgow, M. F. Interrante, E. M. Storm, J. R. Cochran, Dual display of proteins on the yeast cell surface simplifies quantification of binding interactions and enzymatic bioconjugation reactions. *Biotechnol. J.* **12**, 1600696 (2017).
7. Q. Wang, *et al.*, Structural and Functional Basis of SARS-CoV-2 Entry by Using Human ACE2. *Cell* **181**, 894-904.e9 (2020).
8. C. Lei, *et al.*, Neutralization of SARS-CoV-2 spike pseudotyped virus by recombinant ACE2-Ig. *Nat. Commun.* **11**, 2070 (2020).
9. J. L. Guy, R. M. Jackson, H. A. Jensen, N. M. Hooper, A. J. Turner, Identification of critical active-site residues in angiotensin-converting enzyme-2 (ACE2) by site-directed mutagenesis. *FEBS J.* **272**, 3512–3520 (2005).
10. R. Yan, *et al.*, Structural basis for the recognition of SARS-CoV-2 by full-length human ACE2. *Science* **367**, 1444–1448 (2020).
11. F. Li, W. Li, M. Farzan, S. C. Harrison, Structure of SARS Coronavirus Spike Receptor-Binding Domain Complexed with Receptor. *Science* **309**, 1864–1868 (2005).
12. K. Wu, W. Li, G. Peng, F. Li, Crystal structure of NL63 respiratory coronavirus receptor-binding domain complexed with its human receptor. *Proc. Natl. Acad. Sci. U. S. A.* **106**, 19970–19974 (2009).

Appendix 1: Sequences of ACE2 mutants in this work

13	CAATCTACCATCGAAGAGCAGGCCAAAACATTCCTCGACAAGTTTAATCACGAGGCTGAAGACC<br>TTTTCTACCAATCAAGTCTGGCTAGCTGGAATTACAATACAAACATTACAGAGGAGAACGTACA<br>AAACATGAATAACGCAGGGGACAAGTGGAGCGCATTCCTTAAGGAACAAAGTACCCTTGCGCAA<br>ATGTATCCGCTGCAAGAGATTCAAAACCTGACGGTTAAGCTGCAACTTCAGGCCCTCCAACAAA<br>ATGGAAGTTCAGTCTTGTCAGAAGACAAAAGCAAGCGACTGAACACCATCCTTAACACCATGTC<br>AACCATATATTCAACAGGTAAAGTTTGCAATCCGGATAACCCCCAAGAATGTTTGCTTCTTGAA<br>CCCGGTCTCAACGAAATTATGGCCAACAGTCTTGATTACAACGAGCGATTGTGGGCATGGGAAA<br>GTTGGAGGAGTGAGGTAGGCAAACAGTTGAGACCTCTTTATGAAGAGTACGTTGTCCTTAAAAA<br>TGAAATGGCTCGCGCGAATCATTATGAAGACTATGGTGACTACTGGAGGGGGGATTATGAGGTG<br>AACGGGGTGGACGGATACGATTACTCTAGGGGCCAGCTGATAGAGGATGTCGAGCACACCTTTG<br>AGGAGATTAAGCCGTTGTACGAACATTTGCACGCCTATGTCAGGGCTAAGCTCATGAACGCTTA<br>TCCGAGTTATATCTCCCCGATAGGATGCTTGCCTGCTCACTTGTTGGGCGATATGTGGGGACGC<br>TTTTGGACCAACTTGTATTCCCTTACGGTACCGTTCGGCCAGAAACCAAATATCGACGTGACAG<br>ACGCAATGGTGGATCAAGCATGGGATGCGCAACGAATCTTCAAGGAGGCAGAAAAATTTTTCGT<br>TTCAGTTGGACTCCCAAACATGACGCAGGGTTTCTGGGAGAACTCAATGTTGACAGATCCAGGT<br>AATGTGCAGAAAGCGGTTTGCCACCCTACTGCATGGGATCTTGGTAAAGGGGACTTCCGCATAC<br>TCATGTGTACGAAAGTAACTATGGACGACTTTCTTACTGCGCACCACGAGATGGGGCACATACA<br>ATACGATATGGCGTACGCAGCTCAACCTTTCCTTCTGCGGAACGGGGCGAATGAAGGATTTCAC<br>GAGGCAGTGGGTGAGATTATGTCCCTGTCAGCTGCCACTCCGAAACATCTGAAAAGCATCGGCC<br>TGTTGAGCCCAGACTTCCAAGAAGATAATGAGACCGAAATAAACTTCCTTCTGAAGCAAGCACT<br>GACTATTGTAGGTACCTTGCCCTTTACCTACATGCTGGAGAAGTGGAGGTGGATGGTATTTAAG<br>GGGGAGATACCGAAAGATCAATGGATGAAAAAGTGGTGGGAAATGAAAAGGGAGATCGTTGGCG<br>TAGTTGAACCAGTACCGCATGATGAGACGTACTGCGATCCGGCTAGTCTGTTCCATGTCTCTAA<br>TGATTACTCTTTCATCCGCTACTACACCCGCACGCTGTATCAATTCCAGTTCCAAGAAGCTCTC<br>TGTCAGGCTGCCAAGCACGAAGGACCGCTGCACAAATGCGACATTAGCAATTCTACAGAGGCGG<br>GTCAGAAGTTGTTCAATATGCTTAGACTGGGGAAGAGCGAACCGTGGACGCTCGCTTTGGAGAA<br>CGTTGTTGGAGCTAAGAATATGAACGTCAGGCCCTTGCTGAATTACTTTGAACCTCTGTTTACG<br>TGGTTGAAAGACCAAAATAAAAACTCCTTTGTTGGGTGGAGTACTGACTGGTCCCCCTATGCG
208	CAATCTACCATCGAAGAGCAGGCCAAAACATTCCTCGACAAGTTTAATCACGAGGCTGAAGACC<br>TTTTCTACCAATCAAGTCTGGCTAGCTGGAATTACAATACAAACATTACAGAGGAGAACGTACA<br>AAACATGAATAACGCAGGGGACAAGTGGAGCGCATTCCTTAAGGAACAAAGTACCCTTGCGCAA<br>ATGTATCCGCTGCAAGAGATTCAAAACCTGACGGTTAAGCTGCAACTTCAGGCCCTCCAACAAA<br>ATGGAAGTTCAGTCTTGTCAGAAGACAAAAGCAAGCGACTGAACACCATCCTTAACACCATGTC<br>AACCATATATTCAACAGGTAAAGTTTGCAATCCGGATAACCCCCAAGAATGTTTGCTTCTTGAA<br>CCCGGTCTCAACGAAATTATGGCCAACAGTCTTGATTACAACGAGCGATTGTGGGCATGGGAAA<br>GTTGGAGGAGTGAGGTAGGCAAACAGTTGAGACCTCTTTATGAAGAGTACGTTGTCCTTAAAAA<br>TGAAATGGCTCGCGCGAATCATTATGAAGACTATGGTGACTACTGGAGGGGGGATTATGAGGTG<br>AACGGGGTGGACGGATACGATTACTCTAGGGGCCAGCTGATAGAGGATGTCGAGCACACCTTTG<br>AGGAGATTAAGCCGTTGTACGAACATTTGCACGCCTATGTCAGGGCTAAGCTCATGAACGCTTA<br>TCCGAGTTATATCTCCCCGATAGGATGCTTGCCTGCTCACTTGTTGGGCGATATGTGGGGACGC<br>TTTTGGACCAACTTGTATTCCCTTACGGTACCGTTCGGCCAGAAACCAAATATCGACGTGACAG<br>ACGCAATGGTGGATCAAGCATGGGATGCGCAACGAATCTTCAAGGAGGCAGAAAAATTTTTCGT<br>TTCAGTTGGACTCCCAAACATGACGCAGGGTTTCTGGGAGAACTCAATGTTGACAGATCCAGGT<br>AATGTGCAGAAAGCGGTTTGCCACCCTACTGCATGGGATCTTGGTAAAGGGGACTTCCGCATAC<br>TCATGTGTACGAAAGTAACTATGGACGACTTTCTTACTGCGCACCACGAGATGGGGCACATACA<br>ATACGATATGGCGTACGCAGCTCAACCTTTCCTTCTGCGGAACGGGGCGAATGAAGGATTTCAC

	GAGGCAGTGGGTGAGATTATGTCCCTGTCAGCTGCCACTCCGAAACATCTGAAAAGCATCGGCC<br>TGTTGAGCCCAGACTTCCAAGAAGATAATGAGACCGAAATAAACTTCCTTCTGAAGCAAGCACT<br>GACTATTGTAGGTACCTTGCCCTTTACATACATGCTGGAGAAGTGGAGGTGGATGGTATTTAAG<br>GGGGAGATACCGAAAGATCAATGGATGAAAAAGTGGTGGGAAATGAAAAGGGAGATCGTTGGCG<br>TAGTTGAACCAGTACCGCATGATGAGACGTACTGCGATCCGGCTAGTCTGTTCCATGTCTCTAA<br>TGATTACTCTTTCATCCGCTACTACACCCGCACGCTGTATCAATTCCAGTTCCAAGAAGCTCTC<br>TGTCAGGCTGCCAAGCACGAAGGACCGCTGCACAAATGCGACATTAGCAATTCTACAGAGGCGG<br>GTCAGAAGTTGTTCAATATGCTTAGACTGGGGAAGAGCGAACCGTGGACGCTCGCTTTGGAGAA<br>CGTTGTTGGAGCTAAGAATATGAACGTCAGGCCCTTGCTGAATTACTTTGAACCTCTGTTTACG<br>TGGTTGAAAGACCAAAATAAAAACTCCTTTGTTGGGTGGAGTACTGACTGGTCCCCCTATGCGG<br>ACCAAAGCATCAAAGTGAGGATAAGCCTAAAATCAGCTCTTGGAGATAAAGCATATGAATGGAA<br>CGACAATGAAATGTACCTGTTCCGATCATCTGTTGCATATGCTATGAGGCAGTACTTTTTAAAA<br>GTAAAAAATCAGATGATTCTTTTTGGGGAGGAGGATGTGCGAGTGGCTAATTTGAAACCAAGAA<br>TCTCCTTTAATTTCTTTGTCACTGCACCTAAAAATGTGTCTGATATCATTCCTAGAACTGAAGT<br>TGAAAAGGCCATCAGGATGTCCCGGAGCCGTATCAATGATGCTTTCCGTCTGAATGACAACAGC<br>CTAGAGTTTCTGGGGATACAGCCAACACTTGGACCTCCTAACCAGCCCCCTGTTTCC
Y208	CAATCTACCATCGAAGAGCAGGCCAAAACATTCCTCGACAAGTTTAATCACGAGGCTGAAGACC<br>TTTTCTACCAATCAAGTCTGGCTAGCTGGAATTACAATACAAACATTACAGAGGAGAACGTACA<br>AAACATGAATAACGCAGGGGACAAGTGGAGCGCATTCCTTAAGGAACAAAGTACCCTTGCGCAA<br>ATGTATCCGCTGCAAGAGATTCAAAACCTGACGGTTAAGCTGCAACTTCAGGCCCTCCAACAAA<br>ATGGATCCTCAGTCTTGTCAGAAGACAAAAGCAAGCGACTGAACACCATCCTTAACACCATGTC<br>AACCATATATTCAACAGGTAAAGTTTGCAATCCGGATAACCCCCAAGAATGTTTGCTTCTTGAA<br>CCCGGTCTCAACGAAATTATGGCCAACAGTCTTGATTACAACGAGCGATTGTGGGCATGGGAAA<br>GTTGGAGGAGTGAGGTAGGCAAACAGTTGAGACCTCTTTATGAAGAGTACGTTGTCCTTAAAAA<br>TGAAATGGCTCGCGCGAATCATTATGAAGACTATGGTGACTACTGGAGGGGGGATTATGAGGTG<br>AACGGGGTGGACGGATACGATTACTCTAGGGGCCAGCTGATAGAGGATGTCGAGCACACCTTTG<br>AGGAGATTAAGCCGTTGTACGAACATTTGCACGCCTATGTCAGGGCTAAGCTCATGAACGCTTA<br>TCCGAGTTATATCTCCCCGATAGGATGCTTGCCTGCTCACTTGTTGGGCGATATGTGGGGACGC<br>TTTTGGACCAACTTGTATTCCCTTACGGTACCGTTCGGCCAGAAACCAAATATCGACGTGACAG<br>ACGCAATGGTGGATCAAGCATGGGATGCGCAACGAATCTTCAAGGAGGCAGAAAAATTTTTCGT<br>TTCAGTTGGACTCCCAAACATGACGCAGGGTTTCTGGGAGAACTCAATGTTGACAGATCCAGGT<br>AATGTGCAGAAAGCGGTTTGCCACCCTACTGCATGGGATCTTGGTAAAGGGGACTTCCGCATAC<br>TCATGTGTACGAAAGTAACTATGGACGACTTTCTTACTGCGCACCACGAGATGGGGCACATACA<br>ATACGATATGGCGTACGCAGCTCAACCTTTCCTTCTGCGGAACGGGGCGAATGAAGGATTTCAC<br>GAGGCAGTGGGTGAGATTATGTCCCTGTCAGCTGCCACTCCGAAACATCTGAAAAGCATCGGCC<br>TGTTGAGCCCAGACTTCCAAGAAGATAATGAGACCGAAATAAACTTCCTTCTGAAGCAAGCACT<br>GACTATTGTAGGTACCTTGCCCTTTACATACATGCTGGAGAAGTGGAGGTGGATGGTATTTAAG<br>GGGGAGATACCGAAAGATCAATGGATGAAAAAGTGGTGGGAAATGAAAAGGGAGATCGTTGGCG<br>TAGTTGAACCAGTACCGCATGATGAGACGTACTGCGATCCGGCTAGTCTGTTCCATGTCTCTAA<br>TGATTACTCTTTCATCCGCTACTACACCCGCACGCTGTATCAATTCCAGTTCCAAGAAGCTCTC<br>TGTCAGGCTGCCAAGCACGAAGGACCGCTGCACAAATGCGACATTAGCAATTCTACAGAGGCGG<br>GTCAGAAGTTGTTCAATATGCTTAGACTGGGGAAGAGCGAACCGTGGACGCTCGCTTTGGAGAA<br>CGTTGTTGGAGCTAAGAATATGAACGTCAGGCCCTTGCTGAATTACTTTGAACCTCTGTTTACG<br>TGGTTGAAAGACCAAAATAAAAACTCCTTTGTTGGGTGGAGTACTGACTGGTCCCCCTATGCG
14	CAATCTACCATCGAAGAGCAGGCCAAAACATTCCTCGACAAGTTTAATGTCGAGGCTGAAGACC<br>TTTTCTACCAATCAAGTCTGGCTAGCTGGAATTACAATACAAACATTACAGAGGAGAACGTACA<br>AAACATGAATAACGCAGGGGACAAGTGGAGCGCATTCCTTAAGGAACAAAGTACCCTTGCGCAA

	ATGTATCCGCTGCAAGAGATTCAAAACCTGACGGTTAAGCTGCAACTTCAGGCCCTCCAACAAA<br>ATGGAAGTTCAGTCTTGTCAGAAGACAAAAGCAAGCGACTGAACACCATCCTTAACACCATGTC<br>AACCATATATTCAACAGGTAAAGTTTGCAATCCGGATAACCCCCAAGAATGTTTGCTTCTTGAA<br>CCCGGTCTCAACGAAATTATGGCCAACAGTCTTGATTACAACGAGCGATTGTGGGCATGGGAAA<br>GTTGGAGGAGTGAGGTAGGCAAACAGTTGAGACCTCTTTATGAAGAGTACGTTGTCCTTAAAAA<br>TGAAATGGCTCGCGCGAATCATTATGAAGACTATGGTGACTACTGGAGGGGGGATTATGAGGTG<br>AACGGGGTGGACGGATACGATTACTCTAGGGGCCAGCTGATAGAGGATGTCGAGCACACCTTTG<br>AGGAGATTAAGCCGTTGTACGAACATTTGCACGCCTATGTCAGGGCTAAGCTCATGAACGCTTA<br>TCCGAGTTATATCTCCCCGATAGGATGCTTGCCTGCTCACTTGTTGGGCGATATGTGGGGACGC<br>TTTTGGACCAACTTGTATTCCCTTACGGTACCGTTCGGCCAGAAACCAAATATCGACGTGACAG<br>ACGCAATGGTGGATCAAGCATGGGATGCGCAACGAATCTTCAAGGAGGCAGAAAAATTTTTCGT<br>TTCAGTTGGACTCCCAAACATGACGCAGGGTTTCTGGGAGAACTCAATGTTGACAGATCCAGGT<br>AATGTGCAGAAAGCGGTTTGCCACCCTACTGCATGGGATCTTGGTAAAGGGGACTTCCGCATAC<br>TCATGTGTACGAAAGTAACTATGGACGACTTTCTTACTGCGCACCACGAGATGGGGCACATACA<br>ATACGATATGGCGTACGCAGCTCAACCTTTCCTTCTGCGGAACGGGGCGAATGAAGGATTTCAC<br>GAGGCAGTGGGTGAGATTATGTCCCTGTCAGCTGCCACTCCGAAACATCTGAAAAGCATCGGCC<br>TGTTGAGCCCAGACTTCCAAGAAGATAATGAGACCGAAATAAACTTCCTTCTGAAGCAAGCACT<br>GACTATTGTAGGTACCTTGCCCTTTACCTACATGCTGGAGAAGTGGAGGTGGATGGTATTTAAG<br>GGGGAGATACCGAAAGATCAATGGATGAAAAAGTGGTGGGAAATGAAAAGGGAGATCGTTGGCG<br>TAGTTGAACCAGTACCGCATGATGAGACGTACTGCGATCCGGCTAGTCTGTTCCATGTCTCTAA<br>TGATTACTCTTTCATCCGCTACTACACCCGCACGCTGTATCAATTCCAGTTCCAAGAAGCTCTC<br>TGTCAGGCTGCCAAGCACGAAGGACCGCTGCACAAATGCGACATTAGCAATTCTACAGAGGCGG<br>GTCAGAAGTTGTTCAATATGCTTAGACTGGGGAAGAGCGAACCGTGGACGCTCGCTTTGGAGAA<br>CGTTGTTGGAGCTAAGAATATGAACGTCAGGCCCTTGCTGAATTACTTTGAACCTCTGTTTACG<br>TGGTTGAAAGACCAAAATAAAAACTCCTTTGTTGGGTGGAGTACTGACTGGTCCCCCTATGCG
295	CAATCTACCATCGAAGAGCAGGCCAAAACATTCCTCGACAAGTTTAATGTCGAGGCTGAAGACC<br>TTTTCTACCAATCAAGTCTGGCTAGCTGGAATTACAATACAAACATTACAGAGGAGAACGTACA<br>AAACATGAATAACGCAGGGGACAAGTGGAGCGCATTCCTTAAGGAACAAAGTACCCTTGCGCAA<br>ATGTATCCGCTGCAAGAGATTCAAAACCTGACGGTTAAGCTGCAACTTCAGGCCCTCCAACAAA<br>ATGGATCCTCAGTCTTGTCAGAAGACAAAAGCAAGCGACTGAACACCATCCTTAACACCATGTC<br>AACCATATATTCAACAGGTAAAGTTTGCAATCCGGATAACCCCCAAGAATGTTTGCTTCTTGAA<br>CCCGGTCTCAACGAAATTATGGCCAACAGTCTTGATTACAACGAGCGATTGTGGGCATGGGAAA<br>GTTGGAGGAGTGAGGTAGGCAAACAGTTGAGACCTCTTTATGAAGAGTACGTTGTCCTTAAAAA<br>TGAAATGGCTCGCGCGAATCATTATGAAGACTATGGTGACTACTGGAGGGGGGATTATGAGGTG<br>AACGGGGTGGACGGATACGATTACTCTAGGGGCCAGCTGATAGAGGATGTCGAGCACACCTTTG<br>AGGAGATTAAGCCGTTGTACGAACATTTGCACGCCTATGTCAGGGCTAAGCTCATGAACGCTTA<br>TCCGAGTTATATCTCCCCGATAGGATGCTTGCCTGCTCACTTGTTGGGCGATATGTGGGGACGC<br>TTTTGGACCAACTTGTATTCCCTTACGGTACCGTTCGGCCAGAAACCAAATATCGACGTGACAG<br>ACGCAATGGTGGATCAAGCATGGGATGCGCAACGAATCTTCAAGGAGGCAGAAAAATTTTTCGT<br>TTCAGTTGGACTCCCAAACATGACGCAGGGTTTCTGGGAGAACTCAATGTTGACAGATCCAGGT<br>AATGTGCAGAAAGCGGTTTGCCACCCTACTGCATGGGATCTTGGTAAAGGGGACTTCCGCATAC<br>TCATGTGTACGAAAGTAACTATGGACGACTTTCTTACTGCGCACCACGAGATGGGGCACATACA<br>ATACGATATGGCGTACGCAGCTCAACCTTTCCTTCTGCGGAACGGGGCGAATGAAGGATTTCAC<br>GAGGCAGTGGGTGAGATTATGTCCCTGTCAGCTGCCACTCCGAAACATCTGAAAAGCATCGGCC<br>TGTTGAGCCCAGACTTCCAAGAAGATAATGAGACCGAAATAAACTTCCTTCTGAAGCAAGCACT<br>GACTATTGTAGGTACCTTGCCCTTTACATACATGCTGGAGAAGTGGAGGTGGATGGTATTTAAG<br>GGGGAGATACCGAAAGATCAATGGATGAAAAAGTGGTGGGAAATGAAAAGGGAGATCGTTGGCG

	TAGTTGAACCAGTACCGCATGATGAGACGTACTGCGATCCGGCTAGTCTGTTCCATGTCTCTAA<br>TGATTACTCTTTCATCCGCTACTACACCCGCACGCTGTATCAATTCCAGTTCCAAGAAGCTCTC<br>TGTCAGGCTGCCAAGCACGAAGGACCGCTGCACAAATGCGACATTAGCAATTCTACAGAGGCGG<br>GTCAGAAGTTGTTCAATATGCTTAGACTGGGGAAGAGCGAACCGTGGACGCTCGCTTTGGAGAA<br>CGTTGTTGGAGCTAAGAATATGAACGTCAGGCCCTTGCTGAATTACTTTGAACCTCTGTTTACG<br>TGGTTGAAAGACCAAAATAAAAACTCCTTTGTTGGGTGGAGTACTGACTGGTCCCCCTATGCGG<br>ACCAAAGCATCAAAGTGAGGATAAGCCTAAAATCAGCTCTTGGAGATAAAGCATATGAATGGAA<br>CGACAATGAAATGTACCTGTTCCGATCATCTGTTGCATATGCTATGAGGCAGTACTTTTTAAAA<br>GTAAAAAATCAGATGATTCTTTTTGGGGAGGAGGATGTGCGAGTGGCTAATTTGAAACCAAGAA<br>TCTCCTTTAATTTCTTTGTCACTGCACCTAAAAATGTGTCTGATATCATTCCTAGAACTGAAGT<br>TGAAAAGGCCATCAGGATGTCCCGGAGCCGTATCAATGATGCTTTCCGTCTGAATGACAACAGC<br>CTAGAGTTTCTGGGGATACAGCCAACACTTGGACCTCCTAACCAGCCCCCTGTTTCC
19	CAATCTACCATCGAAGAGCAGGCCAAAACATTCCTCGACTTCTTTAATATCCAGGCTGAAGACC<br>TTTTCTACCAATCAAGTCTGGCTAGCTGGAATTACAATACAAACATTACAGAGGAGAACGTACA<br>AAACATGAATAACGCAGGGGACAAGTGGAGCGCATTCCTTAAGGAACAAAGTACCCTTGCGCAA<br>ATGTATCCGCTGCAAGAGATTCAAAACCTGACGGTTAAGCTGCAACTTCAGGCCCTCCAACAAA<br>ATGGAAGTTCAGTCTTGTCAGAAGACAAAAGCAAGCGACTGAACACCATCCTTAACACCATGTC<br>AACCATATATTCAACAGGTAAAGTTTGCAATCCGGATAACCCCCAAGAATGTTTGCTTCTTGAA<br>CCCGGTCTCAACGAAATTATGGCCAACAGTCTTGATTACAACGAGCGATTGTGGGCATGGGAAA<br>GTTGGAGGAGTGAGGTAGGCAAACAGTTGAGACCTCTTTATGAAGAGTACGTTGTCCTTAAAAA<br>TGAAATGGCTCGCGCGAATCATTATGAAGACTATGGTGACTACTGGAGGGGGGATTATGAGGTG<br>AACGGGGTGGACGGATACGATTACTCTAGGGGCCAGCTGATAGAGGATGTCGAGCACACCTTTG<br>AGGAGATTAAGCCGTTGTACGAACATTTGCACGCCTATGTCAGGGCTAAGCTCATGAACGCTTA<br>TCCGAGTTATATCTCCCCGATAGGATGCTTGCCTGCTCACTTGTTGGGCGATATGTGGGGACGC<br>TTTTGGACCAACTTGTATTCCCTTACGGTACCGTTCGGCCAGAAACCAAATATCGACGTGACAG<br>ACGCAATGGTGGATCAAGCATGGGATGCGCAACGAATCTTCAAGGAGGCAGAAAAATTTTTCGT<br>TTCAGTTGGACTCCCAAACATGACGCAGGGTTTCTGGGAGAACTCAATGTTGACAGATCCAGGT<br>AATGTGCAGAAAGCGGTTTGCCACCCTACTGCATGGGATCTTGGTAAAGGGGACTTCCGCATAC<br>TCATGTGTACGAAAGTAACTATGGACGACTTTCTTACTGCGCACCACGAGATGGGGCACATACA<br>ATACGATATGGCGTACGCAGCTCAACCTTTCCTTCTGCGGAACGGGGCGAATGAAGGATTTCAC<br>GAGGCAGTGGGTGAGATTATGTCCCTGTCAGCTGCCACTCCGAAACATCTGAAAAGCATCGGCC<br>TGTTGAGCCCAGACTTCCAAGAAGATAATGAGACCGAAATAAACTTCCTTCTGAAGCAAGCACT<br>GACTATTGTAGGTACCTTGCCCTTTACCTACATGCTGGAGAAGTGGAGGTGGATGGTATTTAAG<br>GGGGAGATACCGAAAGATCAATGGATGAAAAAGTGGTGGGAAATGAAAAGGGAGATCGTTGGCG<br>TAGTTGAACCAGTACCGCATGATGAGACGTACTGCGATCCGGCTAGTCTGTTCCATGTCTCTAA<br>TGATTACTCTTTCATCCGCTACTACACCCGCACGCTGTATCAATTCCAGTTCCAAGAAGCTCTC<br>TGTCAGGCTGCCAAGCACGAAGGACCGCTGCACAAATGCGACATTAGCAATTCTACAGAGGCGG<br>GTCAGAAGTTGTTCAATATGCTTAGACTGGGGAAGAGCGAACCGTGGACGCTCGCTTTGGAGAA<br>CGTTGTTGGAGCTAAGAATATGAACGTCAGGCCCTTGCTGAATTACTTTGAACCTCTGTTTACG<br>TGGTTGAAAGACCAAAATAAAAACTCCTTTGTTGGGTGGAGTACTGACTGGTCCCCCTATGCG
293	CAATCTACCATCGAAGAGCAGGCCAAAACATTCCTCGACTTCTTTAATATCCAGGCTGAAGACC<br>TTTTCTACCAATCAAGTCTGGCTAGCTGGAATTACAATACAAACATTACAGAGGAGAACGTACA<br>AAACATGAATAACGCAGGGGACAAGTGGAGCGCATTCCTTAAGGAACAAAGTACCCTTGCGCAA<br>ATGTATCCGCTGCAAGAGATTCAAAACCTGACGGTTAAGCTGCAACTTCAGGCCCTCCAACAAA<br>ATGGAAGTTCAGTCTTGTCAGAAGACAAAAGCAAGCGACTGAACACCATCCTTAACACCATGTC<br>AACCATATATTCAACAGGTAAAGTTTGCAATCCGGATAACCCCCAAGAATGTTTGCTTCTTGAA<br>CCCGGTCTCAACGAAATTATGGCCAACAGTCTTGATTACAACGAGCGATTGTGGGCATGGGAAA

	GTTGGAGGAGTGAGGTAGGCAAACAGTTGAGACCTCTTTATGAAGAGTACGTTGTCCTTAAAAA<br>TGAAATGGCTCGCGCGAATCATTATGAAGACTATGGTGACTACTGGAGGGGGGATTATGAGGTG<br>AACGGGGTGGACGGATACGATTACTCTAGGGGCCAGCTGATAGAGGATGTCGAGCACACCTTTG<br>AGGAGATTAAGCCGTTGTACGAACATTTGCACGCCTATGTCAGGGCTAAGCTCATGAACGCTTA<br>TCCGAGTTATATCTCCCCGATAGGATGCTTGCCTGCTCACTTGTTGGGCGATATGTGGGGACGC<br>TTTTGGACCAACTTGTATTCCCTTACGGTACCGTTCGGCCAGAAACCAAATATCGACGTGACAG<br>ACGCAATGGTGGATCAAGCATGGGATGCGCAACGAATCTTCAAGGAGGCAGAAAAATTTTTCGT<br>TTCAGTTGGACTCCCAAACATGACGCAGGGTTTCTGGGAGAACTCAATGTTGACAGATCCAGGT<br>AATGTGCAGAAAGCGGTTTGCCACCCTACTGCATGGGATCTTGGTAAAGGGGACTTCCGCATAC<br>TCATGTGTACGAAAGTAACTATGGACGACTTTCTTACTGCGCACCACGAGATGGGGCACATACA<br>ATACGATATGGCGTACGCAGCTCAACCTTTCCTTCTGCGGAACGGGGCGAATGAAGGATTTCAC<br>GAGGCAGTGGGTGAGATTATGTCCCTGTCAGCTGCCACTCCGAAACATCTGAAAAGCATCGGCC<br>TGTTGAGCCCAGACTTCCAAGAAGATAATGAGACCGAAATAAACTTCCTTCTGAAGCAAGCACT<br>GACTATTGTAGGTACCTTGCCCTTTACATACATGCTGGAGAAGTGGAGGTGGATGGTATTTAAG<br>GGGGAGATACCGAAAGATCAATGGATGAAAAAGTGGTGGGAAATGAAAAGGGAGATCGTTGGCG<br>TAGTTGAACCAGTACCGCATGATGAGACGTACTGCGATCCGGCTAGTCTGTTCCATGTCTCTAA<br>TGATTACTCTTTCATCCGCTACTACACCCGCACGCTGTATCAATTCCAGTTCCAAGAAGCTCTC<br>TGTCAGGCTGCCAAGCACGAAGGACCGCTGCACAAATGCGACATTAGCAATTCTACAGAGGCGG<br>GTCAGAAGTTGTTCAATATGCTTAGACTGGGGAAGAGCGAACCGTGGACGCTCGCTTTGGAGAA<br>CGTTGTTGGAGCTAAGAATATGAACGTCAGGCCCTTGCTGAATTACTTTGAACCTCTGTTTACG<br>TGGTTGAAAGACCAAAATAAAAACTCCTTTGTTGGGTGGAGTACTGACTGGTCCCCCTATGCGG<br>ACCAAAGCATCAAAGTGAGGATAAGCCTAAAATCAGCTCTTGGAGATAAAGCATATGAATGGAA<br>CGACAATGAAATGTACCTGTTCCGATCATCTGTTGCATATGCTATGAGGCAGTACTTTTTAAAA<br>GTAAAAAATCAGATGATTCTTTTTGGGGAGGAGGATGTGCGAGTGGCTAATTTGAAACCAAGAA<br>TCTCCTTTAATTTCTTTGTCACTGCACCTAAAAATGTGTCTGATATCATTCCTAGAACTGAAGT<br>TGAAAAGGCCATCAGGATGTCCCGGAGCCGTATCAATGATGCTTTCCGTCTGAATGACAACAGC<br>CTAGAGTTTCTGGGGATACAGCCAACACTTGGACCTCCTAACCAGCCCCCTGTTTCC
117	CAATCTACCATCGAAGAGCAGGCCAAAACATTCCTCGACAAGTTTAATCACGAGGCTGAAGACC<br>TTTTCTACCAATCAAGTCTGGCTAGCTGGAATTACAATACAAACATTACAGAGGAGAACGTACA<br>AAACATGAATAACGCAGGGGACAAGTGGAGCGCATTCCTTAAGGAACAAAGTACCCTTGCGCAA<br>ATGTATCCGCTGCAAGAGATTCAACAACTGACGGTTAAGCTGCAACTTCAGGCCCTCCAACAAA<br>ATGGATCCTCAGTCTTGTCAGAAGACAAAAGCAAGCGACTGAACACCATCCTTAACACCATGTC<br>AACCATATATTCAACAGGTAAAGTTTGCAATCCGGATAACCCCCAAGAATGTTTGCTTCTTGAA<br>CCCGGTCTCAACGAAATTATGGCCAACAGTCTTGATTACAACGAGCGATTGTGGGCATGGGAAA<br>GTTGGAGGAGTGAGGTAGGCAAACAGTTGAGACCTCTTTATGAAGAGTACGTTGTCCTTAAAAA<br>TGAAATGGCTCGCGCGAATCATTATGAAGACTATGGTGACTACTGGAGGGGGGATTATGAGGTG<br>AACGGGGTGGACGGATACGATTACTCTAGGGGCCAGCTGATAGAGGATGTCGAGCACACCTTTG<br>AGGAGATTAAGCCGTTGTACGAACATTTGCACGCCTATGTCAGGGCTAAGCTCATGAACGCTTA<br>TCCGAGTTATATCTCCCCGATAGGATGCTTGCCTGCTCACTTGTTGGGCGATATGTGGGGACGC<br>TTTTGGACCAACTTGTATTCCCTTACGGTACCGTTCGGCCAGAAACCAAATATCGACGTGACAG<br>ACGCAATGGTGGATCAAGCATGGGATGCGCAACGAATCTTCAAGGAGGCAGAAAAATTTTTCGT<br>TTCAGTTGGACTCCCAAACATGACGCAGGGTTTCTGGGAGAACTCAATGTTGACAGATCCAGGT<br>AATGTGCAGAAAGCGGTTTGCCACCCTACTGCATGGGATCTTGGTAAAGGGGACTTCCGCATAC<br>TCATGTGTACGAAAGTAACTATGGACGACTTTCTTACTGCGCACAACGAGATGGGGAACATACA<br>ATACGATATGGCGTACGCAGCTCAACCTTTCCTTCTGCGGAACGGGGCGAATGAAGGATTTCAC<br>GAGGCAGTGGGTGAGATTATGTCCCTGTCAGCTGCCACTCCGAAACATCTGAAAAGCATCGGCC<br>TGTTGAGCCCAGACTTCCAAGAAGATAATGAGACCGAAATAAACTTCCTTCTGAAGCAAGCACT

	GACTATTGTAGGTACCTTGCCCTTTACATACATGCTGGAGAAGTGGAGGTGGATGGTATTTAAG GGGGAGATACCGAAAGATCAATGGATGAAAAAGTGGTGGGAAATGAAAAGGGAGATCGTTGGCG TAGTTGAACCAGTACCGCATGATGAGACGTACTGCGATCCGGCTAGTCTGTTCCATGTCTCTAA TGATTACTCTTTCATCCGCTACTACACCCGCACGCTGTATCAATTCCAGTTCCAAGAAGCTCTC TGTCAGGCTGCCAAGCACGAAGGACCGCTGCACAAATGCGACATTAGCAATTCTACAGAGGCGG GTCAGAAGTTGTTCAATATGCTTAGACTGGGGAAGAGCGAACCGTGGACGCTCGCTTTGGAGAA CGTTGTTGGAGCTAAGAATATGAACGTCAGGCCCTTGCTGAATTACTTTGAACCTCTGTTTACG TGGTTGAAAGACCAAAATAAAAACTCCTTTGTTGGGTGGAGTACTGACTGGTCCCCCTATGCG
Y117	CAATCTACCATCGAAGAGCAGGCCAAAACATTCCTCGACAAGTTTAATCACGAGGCTGAAGACC TTTTCTACCAATCAAGTCTGGCTAGCTGGAATTACAATACAAACATTACAGAGGAGAACGTACA AAACATGAATAACGCAGGGGACAAGTGGAGCGCATTCCTTAAGGAACAAAGTACCCTTGCGCAA ATGTATCCGCTGCAAGAGATTCAACAACTGACGGTTAAGCTGCAACTTCAGGCCCTCCAACAAA ATGGATCCTCAGTCTTGTCAGAAGACAAAAGCAAGCGACTGAACACCATCCTTAACACCATGTC AACCATATATTCAACAGGTAAAGTTTGCAATCCGGATAACCCCCAAGAATGTTTGCTTCTTGAA CCCGGTCTCAACGAAATTATGGCCAACAGTCTTGATTACAACGAGCGATTGTGGGCATGGGAAA GTTGGAGGAGTGAGGTAGGCAAACAGTTGAGACCTCTTTATGAAGAGTACGTTGTCCTTAAAAA TGAAATGGCTCGCGCGAATCATTATGAAGACTATGGTGACTACTGGAGGGGGGATTATGAGGTG AACGGGGTGGACGGATACGATTACTCTAGGGGCCAGCTGATAGAGGATGTCGAGCACACCTTTG AGGAGATTAAGCCGTTGTACGAACATTTGCACGCCTATGTCAGGGCTAAGCTCATGAACGCTTA TCCGAGTTATATCTCCCCGATAGGATGCTTGCCTGCTCACTTGTTGGGCGATATGTGGGGACGC TTTTGGACCAACTTGTATTCCCTTACGGTACCGTTCGGCCAGAAACCAAATATCGACGTGACAG ACGCAATGGTGGATCAAGCATGGGATGCGCAACGAATCTTCAAGGAGGCAGAAAAATTTTTCGT TTCAGTTGGACTCCCAAACATGACGCAGGGTTTCTGGGAGAACTCAATGTTGACAGATCCAGGT AATGTGCAGAAAGCGGTTTGCCACCCTACTGCATGGGATCTTGGTAAAGGGGACTTCCGCATAC TCATGTGTACGAAAGTAACTATGGACGACTTTCTTACTGCGCACCACGAGATGGGGCACATACA ATACGATATGGCGTACGCAGCTCAACCTTTCCTTCTGCGGAACGGGGCGAATGAAGGATTTCAC GAGGCAGTGGGTGAGATTATGTCCCTGTCAGCTGCCACTCCGAAACATCTGAAAAGCATCGGCC TGTTGAGCCCAGACTTCCAAGAAGATAATGAGACCGAAATAAACTTCCTTCTGAAGCAAGCACT GACTATTGTAGGTACCTTGCCCTTTACATACATGCTGGAGAAGTGGAGGTGGATGGTATTTAAG GGGGAGATACCGAAAGATCAATGGATGAAAAAGTGGTGGGAAATGAAAAGGGAGATCGTTGGCG TAGTTGAACCAGTACCGCATGATGAGACGTACTGCGATCCGGCTAGTCTGTTCCATGTCTCTAA TGATTACTCTTTCATCCGCTACTACACCCGCACGCTGTATCAATTCCAGTTCCAAGAAGCTCTC TGTCAGGCTGCCAAGCACGAAGGACCGCTGCACAAATGCGACATTAGCAATTCTACAGAGGCGG GTCAGAAGTTGTTCAATATGCTTAGACTGGGGAAGAGCGAACCGTGGACGCTCGCTTTGGAGAA CGTTGTTGGAGCTAAGAATATGAACGTCAGGCCCTTGCTGAATTACTTTGAACCTCTGTTTACG TGGTTGAAAGACCAAAATAAAAACTCCTTTGTTGGGTGGAGTACTGACTGGTCCCCCTATGCG
118	CAATCTACCATCGAAGAGCAGGCCAAAACATTCCTCGACAAGTTTAATGTCGAGGCTGAAGACC TTTTCTACCAATCAAGTCTGGCTAGCTGGAATTACAATACAAACATTACAGAGGAGAACGTACA AAACATGAATAACGCAGGGGACAAGTGGAGCGCATTCCTTAAGGAACAAAGTACCCTTGCGCAA ATGTATCCGCTGCAAGAGATTCAACAACTGACGGTTAAGCTGCAACTTCAGGCCCTCCAACAAA ATGGATCCTCAGTCTTGTCAGAAGACAAAAGCAAGCGACTGAACACCATCCTTAACACCATGTC AACCATATATTCAACAGGTAAAGTTTGCAATCCGGATAACCCCCAAGAATGTTTGCTTCTTGAA CCCGGTCTCAACGAAATTATGGCCAACAGTCTTGATTACAACGAGCGATTGTGGGCATGGGAAA GTTGGAGGAGTGAGGTAGGCAAACAGTTGAGACCTCTTTATGAAGAGTACGTTGTCCTTAAAAA TGAAATGGCTCGCGCGAATCATTATGAAGACTATGGTGACTACTGGAGGGGGGATTATGAGGTG AACGGGGTGGACGGATACGATTACTCTAGGGGCCAGCTGATAGAGGATGTCGAGCACACCTTTG AGGAGATTAAGCCGTTGTACGAACATTTGCACGCCTATGTCAGGGCTAAGCTCATGAACGCTTA

	TCCGAGTTATATCTCCCCGATAGGATGCTTGCCTGCTCACTTGTTGGGCGATATGTGGGGACGC<br>TTTTGGACCAACTTGTATTCCCTTACGGTACCGTTCGGCCAGAAACCAAATATCGACGTGACAG<br>ACGCAATGGTGGATCAAGCATGGGATGCGCAACGAATCTTCAAGGAGGCAGAAAAATTTTTCGT<br>TTCAGTTGGACTCCCAAACATGACGCAGGGTTTCTGGGAGAACTCAATGTTGACAGATCCAGGT<br>AATGTGCAGAAAGCGGTTTGCCACCCTACTGCATGGGATCTTGGTAAAGGGGACTTCCGCATAC<br>TCATGTGTACGAAAGTAACTATGGACGACTTTCTTACTGCGCACAACGAGATGGGGAACATACA<br>ATACGATATGGCGTACGCAGCTCAACCTTTCCTTCTGCGGAACGGGGCGAATGAAGGATTTCAC<br>GAGGCAGTGGGTGAGATTATGTCCCTGTCAGCTGCCACTCCGAAACATCTGAAAAGCATCGGCC<br>TGTTGAGCCCAGACTTCCAAGAAGATAATGAGACCGAAATAAACTTCCTTCTGAAGCAAGCACT<br>GACTATTGTAGGTACCTTGCCCTTTACATACATGCTGGAGAAGTGGAGGTGGATGGTATTTAAG<br>GGGGAGATACCGAAAGATCAATGGATGAAAAAGTGGTGGGAAATGAAAAGGGAGATCGTTGGCG<br>TAGTTGAACCAGTACCGCATGATGAGACGTACTGCGATCCGGCTAGTCTGTTCCATGTCTCTAA<br>TGATTACTCTTTCATCCGCTACTACACCCGCACGCTGTATCAATTCCAGTTCCAAGAAGCTCTC<br>TGTCAGGCTGCCAAGCACGAAGGACCGCTGCACAAATGCGACATTAGCAATTCTACAGAGGCGG<br>GTCAGAAGTTGTTCAATATGCTTAGACTGGGGAAGAGCGAACCGTGGACGCTCGCTTTGGAGAA<br>CGTTGTTGGAGCTAAGAATATGAACGTCAGGCCCTTGCTGAATTACTTTGAACCTCTGTTTACG<br>TGGTTGAAAGACCAAAATAAAAACTCCTTTGTTGGGTGGAGTACTGACTGGTCCCCCTATGCG
278	CAATCTACCATCGAAGAGCAGGCCAAAACATTCCTCGACAAGTTTAATGTCGAGGCTGAAGACC<br>TTTTCTACCAATCAAGTCTGGCTAGCTGGAATTACAATACAAACATTACAGAGGAGAACGTACA<br>AAACATGAATAACGCAGGGGACAAGTGGAGCGCATTCCTTAAGGAACAAAGTACCCTTGCGCAA<br>ATGTATCCGCTGCAAGAGATTCAACAACTGACGGTTAAGCTGCAACTTCAGGCCCTCCAACAAA<br>ATGGATCCTCAGTCTTGTCAGAAGACAAAAGCAAGCGACTGAACACCATCCTTAACACCATGTC<br>AACCATATATTCAACAGGTAAAGTTTGCAATCCGGATAACCCCCAAGAATGTTTGCTTCTTGAA<br>CCCGGTCTCAACGAAATTATGGCCAACAGTCTTGATTACAACGAGCGATTGTGGGCATGGGAAA<br>GTTGGAGGAGTGAGGTAGGCAAACAGTTGAGACCTCTTTATGAAGAGTACGTTGTCCTTAAAAA<br>TGAAATGGCTCGCGCGAATCATTATGAAGACTATGGTGACTACTGGAGGGGGGATTATGAGGTG<br>AACGGGGTGGACGGATACGATTACTCTAGGGGCCAGCTGATAGAGGATGTCGAGCACACCTTTG<br>AGGAGATTAAGCCGTTGTACGAACATTTGCACGCCTATGTCAGGGCTAAGCTCATGAACGCTTA<br>TCCGAGTTATATCTCCCCGATAGGATGCTTGCCTGCTCACTTGTTGGGCGATATGTGGGGACGC<br>TTTTGGACCAACTTGTATTCCCTTACGGTACCGTTCGGCCAGAAACCAAATATCGACGTGACAG<br>ACGCAATGGTGGATCAAGCATGGGATGCGCAACGAATCTTCAAGGAGGCAGAAAAATTTTTCGT<br>TTCAGTTGGACTCCCAAACATGACGCAGGGTTTCTGGGAGAACTCAATGTTGACAGATCCAGGT<br>AATGTGCAGAAAGCGGTTTGCCTCCCTACTGCATGGGATCTTGGTAAAGGGGACTTCCGCATAC<br>TCATGTGTACGAAAGTAACTATGGACGACTTTCTTACTGCGCACCACGAGATGGGGCACATACA<br>ATACGATATGGCGTACGCAGCTCAACCTTTCCTTCTGCGGAACGGGGCGAATGAAGGATTTCAC<br>GAGGCAGTGGGTGAGATTATGTCCCTGTCAGCTGCCACTCCGAAACATCTGAAAAGCATCGGCC<br>TGTTGAGCCCAGACTTCCAAGAAGATAATGAGACCGAAATAAACTTCCTTCTGAAGCAAGCACT<br>GACTATTGTAGGTACCTTGCCCTTTACATACATGCTGGAGAAGTGGAGGTGGATGGTATTTAAG<br>GGGGAGATACCGAAAGATCAATGGATGAAAAAGTGGTGGGAAATGAAAAGGGAGATCGTTGGCG<br>TAGTTGAACCAGTACCGCATGATGAGACGTACTGCGATCCGGCTAGTCTGTTCCATGTCTCTAA<br>TGATTACTCTTTCATCCGCTACTACACCCGCACGCTGTATCAATTCCAGTTCCAAGAAGCTCTC<br>TGTCAGGCTGCCAAGCACGAAGGACCGCTGCACAAATGCGACATTAGCAATTCTACAGAGGCGG<br>GTCAGAAGTTGTTCAATATGCTTAGACTGGGGAAGAGCGAACCGTGGACGCTCGCTTTGGAGAA<br>CGTTGTTGGAGCTAAGAATATGAACGTCAGGCCCTTGCTGAATTACTTTGAACCTCTGTTTACG<br>TGGTTGAAAGACCAAAATAAAAACTCCTTTGTTGGGTGGAGTACTGACTGGTCCCCCTATGCG
292	CAATCTACCATCGAAGAGCAGGCCAAAACATTCCTCGACAAGTTTAATGTCGAGGCTGAAGACC<br>TTTTCTACCAATCAAGTCTGGCTAGCTGGAATTACAATACAAACATTACAGAGGAGAACGTACA

	AAACATGAATAACGCAGGGGACAAGTGGAGCGCATTCCTTAAGGAACAAAGTACCCTTGCGCAA<br>ATGTATCCGCTGCAAGAGATTCAACAACTGACGGTTAAGCTGCAACTTCAGGCCCTCCAACAAA<br>ATGGATCCTCAGTCTTGTCAGAAGACAAAAGCAAGCGACTGAACACCATCCTTAACACCATGTC<br>AACCATATATTCAACAGGTAAAGTTTGCAATCCGGATAACCCCCAAGAATGTTTGCTTCTTGAA<br>CCCGGTCTCAACGAAATTATGGCCAACAGTCTTGATTACAACGAGCGATTGTGGGCATGGGAAA<br>GTTGGAGGAGTGAGGTAGGCAAACAGTTGAGACCTCTTTATGAAGAGTACGTTGTCCTTAAAAA<br>TGAAATGGCTCGCGCGAATCATTATGAAGACTATGGTGACTACTGGAGGGGGGATTATGAGGTG<br>AACGGGGTGGACGGATACGATTACTCTAGGGGCCAGCTGATAGAGGATGTCGAGCACACCTTTG<br>AGGAGATTAAGCCGTTGTACGAACATTTGCACGCCTATGTCAGGGCTAAGCTCATGAACGCTTA<br>TCCGAGTTATATCTCCCCGATAGGATGCTTGCCTGCTCACTTGTTGGGCGATATGTGGGGACGC<br>TTTTGGACCAACTTGTATTCCCTTACGGTACCGTTCGGCCAGAAACCAAATATCGACGTGACAG<br>ACGCAATGGTGGATCAAGCATGGGATGCGCAACGAATCTTCAAGGAGGCAGAAAAATTTTTCGT<br>TTCAGTTGGACTCCCAAACATGACGCAGGGTTTCTGGGAGAACTCAATGTTGACAGATCCAGGT<br>AATGTGCAGAAAGCGGTTTGCCACCCTACTGCATGGGATCTTGGTAAAGGGGACTTCCGCATAC<br>TCATGTGTACGAAAGTAACTATGGACGACTTTCTTACTGCGCACCACGAGATGGGGCACATACA<br>ATACGATATGGCGTACGCAGCTCAACCTTTCCTTCTGCGGAACGGGGCGAATGAAGGATTTCAC<br>GAGGCAGTGGGTGAGATTATGTCCCTGTCAGCTGCCACTCCGAAACATCTGAAAAGCATCGGCC<br>TGTTGAGCCCAGACTTCCAAGAAGATAATGAGACCGAAATAAACTTCCTTCTGAAGCAAGCACT<br>GACTATTGTAGGTACCTTGCCCTTTACATACATGCTGGAGAAGTGGAGGTGGATGGTATTTAAG<br>GGGGAGATACCGAAAGATCAATGGATGAAAAAGTGGTGGGAAATGAAAAGGGAGATCGTTGGCG<br>TAGTTGAACCAGTACCGCATGATGAGACGTACTGCGATCCGGCTAGTCTGTTCCATGTCTCTAA<br>TGATTACTCTTTCATCCGCTACTACACCCGCACGCTGTATCAATTCCAGTTCCAAGAAGCTCTC<br>TGTCAGGCTGCCAAGCACGAAGGACCGCTGCACAAATGCGACATTAGCAATTCTACAGAGGCGG<br>GTCAGAAGTTGTTCAATATGCTTAGACTGGGGAAGAGCGAACCGTGGACGCTCGCTTTGGAGAA<br>CGTTGTTGGAGCTAAGAATATGAACGTCAGGCCCTTGCTGAATTACTTTGAACCTCTGTTTACG<br>TGGTTGAAAGACCAAAATAAAAACTCCTTTGTTGGGTGGAGTACTGACTGGTCCCCCTATGCGG<br>ACCAAAGCATCAAAGTGAGGATAAGCCTAAAATCAGCTCTTGGAGATAAAGCATATGAATGGAA<br>CGACAATGAAATGTACCTGTTCCGATCATCTGTTGCATATGCTATGAGGCAGTACTTTTTAAAA<br>GTAAAAAATCAGATGATTCTTTTTGGGGAGGAGGATGTGCGAGTGGCTAATTTGAAACCAAGAA<br>TCTCCTTTAATTTCTTTGTCACTGCACCTAAAAATGTGTCTGATATCATTCCTAGAACTGAAGT<br>TGAAAAGGCCATCAGGATGTCCCGGAGCCGTATCAATGATGCTTTCCGTCTGAATGACAACAGC<br>CTAGAGTTTCTGGGGATACAGCCAACACTTGGACCTCCTAACCAGCCCCCTGTTTCC
310	CAATCTACCATCGAAGAGCAGGTTAAATATTTCCTCGACAAGTTTAATGCTGAGGCTGAAGACC<br>TTGATTACCAATCAAGTCTGGCTAGCTGGAATTACAATACAAACATTACAGAGGAGAACGTACA<br>AAACATGAATAACGCAGGGGACAAGTGGAGCGCATTCCTTAAGGAACAAAGTACCCTTGCGCAA<br>ATGTATCCGCTGCAAGAGATTCAAAACCTGACGGTTAAGCTGCAACTTCAGGCCCTCCAACAAA<br>ATGGATCCTCAGTCTTGTCAGAAGACAAAAGCAAGCGACTGAACACCATCCTTAACACCATGTC<br>AACCATATATTCAACAGGTAAAGTTTGCAATCCGGATAACCCCCAAGAATGTTTGCTTCTTGAA<br>CCCGGTCTCAACGAAATTATGGCCAACAGTCTTGATTACAACGAGCGATTGTGGGCATGGGAAA<br>GTTGGAGGAGTGAGGTAGGCAAACAGTTGAGACCTCTTTATGAAGAGTACGTTGTCCTTAAAAA<br>TGAAATGGCTCGCGCGAATCATTATGAAGACTATGGTGACTACTGGAGGGGGGATTATGAGGTG<br>AACGGGGTGGACGGATACGATTACTCTAGGGGCCAGCTGATAGAGGATGTCGAGCACACCTTTG<br>AGGAGATTAAGCCGTTGTACGAACATTTGCACGCCTATGTCAGGGCTAAGCTCATGAACGCTTA<br>TCCGAGTTATATCTCCCCGATAGGATGCTTGCCTGCTCACTTGTTGGGCGATATGTGGGGACGC<br>TTTTGGACCAACTTGTATTCCCTTACGGTACCGTTCGGCCAGAAACCAAATATCGACGTGACAG<br>ACGCAATGGTGGATCAAGCATGGGATGCGCAACGAATCTTCAAGGAGGCAGAAAAATTTTTCGT<br>TTCAGTTGGACTCCCAAACATGACGCAGGGTTTCTGGGAGAACTCAATGTTGACAGATCCAGGT

	AATGTGCAGAAAGCGGTTTGCCTCCCTACTGCATGGGATCTTGGTAAAGGGGACTTCCGCATAC<br>TCATGTGTACGAAAGTAACTATGGACGACTTTCTTACTGCGCACCACGAGATGGGGCACATACA<br>ATACGATATGGCGTACGCAGCTCAACCTTTCCTTCTGCGGAACGGGGCGAATGAAGGATTTCAC<br>GAGGCAGTGGGTGAGATTATGTCCCTGTCAGCTGCCACTCCGAAACATCTGAAAAGCATCGGCC<br>TGTTGAGCCCAGACTTCCAAGAAGATAATGAGACCGAAATAAACTTCCTTCTGAAGCAAGCACT<br>GACTATTGTAGGTACCTTGCCCTTTACATACATGCTGGAGAAGTGGAGGTGGATGGTATTTAAG<br>GGGGAGATACCGAAAGATCAATGGATGAAAAAGTGGTGGGAAATGAAAAGGGAGATCGTTGGCG<br>TAGTTGAACCAGTACCGCATGATGAGACGTACTGCGATCCGGCTAGTCTGTTCCATGTCTCTAA<br>TGATTACTCTTTCATCCGCTACTACACCCGCACGCTGTATCAATTCCAGTTCCAAGAAGCTCTC<br>TGTCAGGCTGCCAAGCACGAAGGACCGCTGCACAAATGCGACATTAGCAATTCTACAGAGGCGG<br>GTCAGAAGTTGTTCAATATGCTTAGACTGGGGAAGAGCGAACCGTGGACGCTCGCTTTGGAGAA<br>CGTTGTTGGAGCTAAGAATATGAACGTCAGGCCCTTGCTGAATTACTTTGAACCTCTGTTTACG<br>TGGTTGAAAGACCAAAATAAAAACTCCTTTGTTGGGTGGAGTACTGACTGGTCCCCCTATGCGG<br>ACCAAAGCATCAAAGTGAGGATAAGCCTAAAATCAGCTCTTGGAGATAAAGCATATGAATGGAA<br>CGACAATGAAATGTACCTGTTCCGATCATCTGTTGCATATGCTATGAGGCAGTACTTTTTAAAA<br>GTAAAAAATCAGATGATTCTTTTTGGGGAGGAGGATGTGCGAGTGGCTAATTTGAAACCAAGAA<br>TCTCCTTTAATTTCTTTGTCACTGCACCTAAAAATGTGTCTGATATCATTCCTAGAACTGAAGT<br>TGAAAAGGCCATCAGGATGTCCCGGAGCCGTATCAATGATGCTTTCCGTCTGAATGACAACAGC<br>CTAGAGTTTCTGGGGATACAGCCAACACTTGGACCTCCTAACCAGCCCCCTGTTTCC
311	CAATCTACCATCGAAGAGCAGGCCAAAACATTCCTCGACTATTTTAATCACGAGGCTGAAGACC<br>TTTTCTACCAATCAAGTCTGGCTAGCTGGAATTACAATACAAACATTACAGAGGAGAACGTACA<br>AAACATGAATAACGCAGGGGACAAGGTTAGCGCATTCCTTAAGGAACAAAGTACCACTGCGCAA<br>ATGTATCCGCTGCAAGAGATTCAAAACCCAACGGTTAAGCTGCAACTTCAGGCCCTCCAACAAA<br>ATGGATCCTCAGTCTTGTCAGAAGACAAAAGCAAGCGACTGAACACCATCCTTAACACCATGTC<br>AACCATATATTCAACAGGTAAAGTTTGCAATCCGGATAACCCCCAAGAATGTTTGCTTCTTGAA<br>CCCGGTCTCAACGAAATTATGGCCAACAGTCTTGATTACAACGAGCGATTGTGGGCATGGGAAA<br>GTTGGAGGAGTGAGGTAGGCAAACAGTTGAGACCTCTTTATGAAGAGTACGTTGTCCTTAAAAA<br>TGAAATGGCTCGCGCGAATCATTATGAAGACTATGGTGACTACTGGAGGGGGGATTATGAGGTG<br>AACGGGGTGGACGGATACGATTACTCTAGGGGCCAGCTGATAGAGGATGTCGAGCACACCTTTG<br>AGGAGATTAAGCCGTTGTACGAACATTTGCACGCCTATGTCAGGGCTAAGCTCATGAACGCTTA<br>TCCGAGTTATATCTCCCCGATAGGATGCTTGCCTGCTCACTTGTTGGGCGATATGTGGGGACGC<br>TTTTGGACCAACTTGTATTCCCTTACGGTACCGTTCGGCCAGAAACCAAATATCGACGTGACAG<br>ACGCAATGGTGGATCAAGCATGGGATGCGCAACGAATCTTCAAGGAGGCAGAAAAATTTTTCGT<br>TTCAGTTGGACTCCCAAACATGACGCAGGGTTTCTGGGAGAACTCAATGTTGACAGATCCAGGT<br>AATGTGCAGAAAGCGGTTTGCCTCCCTACTGCATGGGATCTTGGTAAAGGGGACTTCCGCATAC<br>TCATGTGTACGAAAGTAACTATGGACGACTTTCTTACTGCGCACCACGAGATGGGGCACATACA<br>ATACGATATGGCGTACGCAGCTCAACCTTTCCTTCTGCGGAACGGGGCGAATGAAGGATTTCAC<br>GAGGCAGTGGGTGAGATTATGTCCCTGTCAGCTGCCACTCCGAAACATCTGAAAAGCATCGGCC<br>TGTTGAGCCCAGACTTCCAAGAAGATAATGAGACCGAAATAAACTTCCTTCTGAAGCAAGCACT<br>GACTATTGTAGGTACCTTGCCCTTTACATACATGCTGGAGAAGTGGAGGTGGATGGTATTTAAG<br>GGGGAGATACCGAAAGATCAATGGATGAAAAAGTGGTGGGAAATGAAAAGGGAGATCGTTGGCG<br>TAGTTGAACCAGTACCGCATGATGAGACGTACTGCGATCCGGCTAGTCTGTTCCATGTCTCTAA<br>TGATTACTCTTTCATCCGCTACTACACCCGCACGCTGTATCAATTCCAGTTCCAAGAAGCTCTC<br>TGTCAGGCTGCCAAGCACGAAGGACCGCTGCACAAATGCGACATTAGCAATTCTACAGAGGCGG<br>GTCAGAAGTTGTTCAATATGCTTAGACTGGGGAAGAGCGAACCGTGGACGCTCGCTTTGGAGAA<br>CGTTGTTGGAGCTAAGAATATGAACGTCAGGCCCTTGCTGAATTACTTTGAACCTCTGTTTACG<br>TGGTTGAAAGACCAAAATAAAAACTCCTTTGTTGGGTGGAGTACTGACTGGTCCCCCTATGCGG

	ACCAAAGCATCAAAGTGAGGATAAGCCTAAAATCAGCTCTTGGAGATAAAGCATATGAATGGAA<br>CGACAATGAAATGTACCTGTTCCGATCATCTGTTGCATATGCTATGAGGCAGTACTTTTTAAAA<br>GTAAAAAATCAGATGATTCTTTTTGGGGAGGAGGATGTGCGAGTGGCTAATTTGAAACCAAGAA<br>TCTCCTTTAATTTCTTTGTCACTGCACCTAAAAATGTGTCTGATATCATTCCTAGAACTGAAGT<br>TGAAAAGGCCATCAGGATGTCCCGGAGCCGTATCAATGATGCTTTCCGTCTGAATGACAACAGC<br>CTAGAGTTTCTGGGGATACAGCCAACACTTGGACCTCCTAACCAGCCCCCTGTTTCC
312	CAATCTACCATCGAAGAGCAGGCCAAATATTTCCTCGACAAGTTTAATGCTGAGGCTGAAGACC<br>TTTTCTACCAATCAAGTCTGGCTAGCTGGAATTACAATACAAACATTACAGAGGAGAACGTACA<br>AAACATGAATAACGCAGGGGACAAGTGGAGCGCATTCCTTAAGGAACAAAGTACCCTTGCGCAA<br>ATGTATCCGCTGCAAGAGATTCAACAACTGACGGTTAAGCTGCAACTTCAGGCCCTCCAACAAA<br>ATGGATCCTCAGTCTTGTCAGAAGACAAAAGCAAGCGACTGAACACCATCCTTAACACCATGTC<br>AACCATATATTCAACAGGTAAAGTTTGCAATCCGGATAACCCCCAAGAATGTTTGCTTCTTGAA<br>CCCGGTCTCAACGAAATTATGGCCAACAGTCTTGATTACAACGAGCGATTGTGGGCATGGGAAA<br>GTTGGAGGAGTGAGGTAGGCAAACAGTTGAGACCTCTTTATGAAGAGTACGTTGTCCTTAAAAA<br>TGAAATGGCTCGCGCGAATCATTATGAAGACTATGGTGACTACTGGAGGGGGGATTATGAGGTG<br>AACGGGGTGGACGGATACGATTACTCTAGGGGCCAGCTGATAGAGGATGTCGAGCACACCTTTG<br>AGGAGATTAAGCCGTTGTACGAACATTTGCACGCCTATGTCAGGGCTAAGCTCATGAACGCTTA<br>TCCGAGTTATATCTCCCCGATAGGATGCTTGCCTGCTCACTTGTTGGGCGATATGTGGGGACGC<br>TTTTGGACCAACTTGTATTCCCTTACGGTACCGTTCGGCCAGAAACCAAATATCGACGTGACAG<br>ACGCAATGGTGGATCAAGCATGGGATGCGCAACGAATCTTCAAGGAGGCAGAAAAATTTTTCGT<br>TTCAGTTGGACTCCCAAACATGACGCAGGGTTTCTGGGAGAACTCAATGTTGACAGATCCAGGT<br>AATGTGCAGAAAGCGGTTTGCCTCCCTACTGCATGGGATCTTGGTAAAGGGGACTTCCGCATAC<br>TCATGTGTACGAAAGTAACTATGGACGACTTTCTTACTGCGCACCACGAGATGGGGCACATACA<br>ATACGATATGGCGTACGCAGCTCAACCTTTCCTTCTGCGGAACGGGGCGAATGAAGGATTTCAC<br>GAGGCAGTGGGTGAGATTATGTCCCTGTCAGCTGCCACTCCGAAACATCTGAAAAGCATCGGCC<br>TGTTGAGCCCAGACTTCCAAGAAGATAATGAGACCGAAATAAACTTCCTTCTGAAGCAAGCACT<br>GACTATTGTAGGTACCTTGCCCTTTACATACATGCTGGAGAAGTGGAGGTGGATGGTATTTAAG<br>GGGGAGATACCGAAAGATCAATGGATGAAAAAGTGGTGGGAAATGAAAAGGGAGATCGTTGGCG<br>TAGTTGAACCAGTACCGCATGATGAGACGTACTGCGATCCGGCTAGTCTGTTCCATGTCTCTAA<br>TGATTACTCTTTCATCCGCTACTACACCCGCACGCTGTATCAATTCCAGTTCCAAGAAGCTCTC<br>TGTCAGGCTGCCAAGCACGAAGGACCGCTGCACAAATGCGACATTAGCAATTCTACAGAGGCGG<br>GTCAGAAGTTGTTCAATATGCTTAGACTGGGGAAGAGCGAACCGTGGACGCTCGCTTTGGAGAA<br>CGTTGTTGGAGCTAAGAATATGAACGTCAGGCCCTTGCTGAATTACTTTGAACCTCTGTTTACG<br>TGGTTGAAAGACCAAAATAAAAACTCCTTTGTTGGGTGGAGTACTGACTGGTCCCCCTATGCGG<br>ACCAAAGCATCAAAGTGAGGATAAGCCTAAAATCAGCTCTTGGAGATAAAGCATATGAATGGAA<br>CGACAATGAAATGTACCTGTTCCGATCATCTGTTGCATATGCTATGAGGCAGTACTTTTTAAAA<br>GTAAAAAATCAGATGATTCTTTTTGGGGAGGAGGATGTGCGAGTGGCTAATTTGAAACCAAGAA<br>TCTCCTTTAATTTCTTTGTCACTGCACCTAAAAATGTGTCTGATATCATTCCTAGAACTGAAGT<br>TGAAAAGGCCATCAGGATGTCCCGGAGCCGTATCAATGATGCTTTCCGTCTGAATGACAACAGC<br>CTAGAGTTTCTGGGGATACAGCCAACACTTGGACCTCCTAACCAGCCCCCTGTTTCC
313	CAATCTACCATCGAAGAGCAGGCCAAAACATTCCTCGACTTCTTTGATAGCCAGGCTGAAGACC<br>TTTTCTACCAATCAAGTCTGGCTAGCTGGAATTACAATACAAACATTACAGAGGAGAACGTACA<br>AAACATGAATAACGCAGGGGACAAGTGGAGCGCATTCCTTAAGGAACAAAGTACCCTTGCGCAA<br>ATGTATCCGCTGCAAGAGATTCAAAACCTGACGGTTAAGCTGCAACTTCAGGCCCTCCAACAAA<br>ATGGATCCTCAGTCTTGTCAGAAGACAAAAGCAAGCGACTGAACACCATCCTTAACACCATGTC<br>AACCATATATTCAACAGGTAAAGTTTGCAATCCGGATAACCCCCAAGAATGTTTGCTTCTTGAA<br>CCCGGTCTCAACGAAATTATGGCCAACAGTCTTGATTACAACGAGCGATTGTGGGCATGGGAAA

	GTTGGAGGAGTGAGGTAGGCAAACAGTTGAGACCTCTTTATGAAGAGTACGTTGTCCTTAAAAA<br>TGAAATGGCTCGCGCGAATCATTATGAAGACTATGGTGACTACTGGAGGGGGGATTATGAGGTG<br>AACGGGGTGGACGGATACGATTACTCTAGGGGCCAGCTGATAGAGGATGTCGAGCACACCTTTG<br>AGGAGATTAAGCCGTTGTACGAACATTTGCACGCCTATGTCAGGGCTAAGCTCATGAACGCTTA<br>TCCGAGTTATATCTCCCCGATAGGATGCTTGCCTGCTCACTTGTTGGGCGATATGTGGGGACGC<br>TTTTGGACCAACTTGTATTCCCTTACGGTACCGTTCGGCCAGAAACCAAATATCGACGTGACAG<br>ACGCAATGGTGGATCAAGCATGGGATGCGCAACGAATCTTCAAGGAGGCAGAAAAATTTTTCGT<br>TTCAGTTGGACTCCCAAACATGACGCAGGGTTTCTGGGAGAACTCAATGTTGACAGATCCAGGT<br>AATGTGCAGAAAGCGGTTTGCCTCCCTACTGCATGGGATCTTGGTAAAGGGGACTTCCGCATAC<br>TCATGTGTACGAAAGTAACTATGGACGACTTTCTTACTGCGCACCACGAGATGGGGCACATACA<br>ATACGATATGGCGTACGCAGCTCAACCTTTCCTTCTGCGGAACGGGGCGAATGAAGGATTTCAC<br>GAGGCAGTGGGTGAGATTATGTCCCTGTCAGCTGCCACTCCGAAACATCTGAAAAGCATCGGCC<br>TGTTGAGCCCAGACTTCCAAGAAGATAATGAGACCGAAATAAACTTCCTTCTGAAGCAAGCACT<br>GACTATTGTAGGTACCTTGCCCTTTACATACATGCTGGAGAAGTGGAGGTGGATGGTATTTAAG<br>GGGGAGATACCGAAAGATCAATGGATGAAAAAGTGGTGGGAAATGAAAAGGGAGATCGTTGGCG<br>TAGTTGAACCAGTACCGCATGATGAGACGTACTGCGATCCGGCTAGTCTGTTCCATGTCTCTAA<br>TGATTACTCTTTCATCCGCTACTACACCCGCACGCTGTATCAATTCCAGTTCCAAGAAGCTCTC<br>TGTCAGGCTGCCAAGCACGAAGGACCGCTGCACAAATGCGACATTAGCAATTCTACAGAGGCGG<br>GTCAGAAGTTGTTCAATATGCTTAGACTGGGGAAGAGCGAACCGTGGACGCTCGCTTTGGAGAA<br>CGTTGTTGGAGCTAAGAATATGAACGTCAGGCCCTTGCTGAATTACTTTGAACCTCTGTTTACG<br>TGGTTGAAAGACCAAAATAAAAACTCCTTTGTTGGGTGGAGTACTGACTGGTCCCCCTATGCGG<br>ACCAAAGCATCAAAGTGAGGATAAGCCTAAAATCAGCTCTTGGAGATAAAGCATATGAATGGAA<br>CGACAATGAAATGTACCTGTTCCGATCATCTGTTGCATATGCTATGAGGCAGTACTTTTTAAAA<br>GTAAAAAATCAGATGATTCTTTTTGGGGAGGAGGATGTGCGAGTGGCTAATTTGAAACCAAGAA<br>TCTCCTTTAATTTCTTTGTCACTGCACCTAAAAATGTGTCTGATATCATTCCTAGAACTGAAGT<br>TGAAAAGGCCATCAGGATGTCCCGGAGCCGTATCAATGATGCTTTCCGTCTGAATGACAACAGC<br>CTAGAGTTTCTGGGGATACAGCCAACACTTGGACCTCCTAACCAGCCCCCTGTTTCC
353	CAATCTACCATCGAAGAGCAGGCCAAAGCATTCCTCGACTTCTTTGATAGCCAGGCTGAAGACC<br>TTTTCTACCAATCAAGTCTGGCTAGCTGGAATTACAATACAAACATTACAGAGGAGAACGTACA<br>AGACATGAATAACGCAGGGGACAGGTGGAGCGCATTCCTTAAGGAACAAAGTACCCCTGCGCAA<br>ATGTATCCGCTGCAAGAGATTCAAAACCTGACGGTTAAGCTGCAACTTCAGGCCCTCCAACAAA<br>ATGGATCCTCAGTCTTGTCAGAAGACAAAAGCAAGCGACTGAACACCATCCTTAACACCATGTC<br>AACCATATATTCAACAGGTAAAGTTTGCAATCCGGATAACCCCCAAGAATGTTTGCTTCTTGAA<br>CCCGGTCTCAACGAAATTATGGCCAACAGTCTTGATTACAACGAGCGATTGTGGGCATGGGAAA<br>GTTGGAGGAGTGAGGTAGGCAAACAGTTGAGACCTCTTTATGAAGAGTACGTTGTCCTTAAAAA<br>TGAAATGGCTCGCGCGAATCATTATGAAGACTATGGTGACTACTGGAGGGGGGATTATGAGGTG<br>AACGGGGTGGACGGATACGATTACTCTAGGGGCCAGCTGATAGAGGATGTCGAGCACACCTTTG<br>AGGAGATTAAGCCGTTGTACGAACATTTGCACGCCTATGTCAGGGCTAAGCTCATGAACGCTTA<br>TCCGAGTTATATCTCCCCGATAGGATGCTTGCCTGCTCACTTGTTGGGCGATATGTGGGGACGC<br>TTTTGGACCAACTTGTATTCCCTTACGGTACCGTTCGGCCAGAAACCAAATATCGACGTGACAG<br>ACGCAATGGTGGATCAAGCATGGGATGCGCAACGAATCTTCAAGGAGGCAGAAAAATTTTTCGT<br>TTCAGTTGGACTCCCAAACATGACGCAGGGTTTCTGGGAGAACTCAATGTTGACAGATCCAGGT<br>AATGTGCAGAAAGCGGTTTGCCTCCCTACTGCATGGGATCTTGGTAAAGGGGACTTCCGCATAC<br>TCATGTGTACGAAAGTAACTATGGACGACTTTCTTACTGCGCACCACGAGATGGGGCACATACA<br>ATACGATATGGCGTACGCAGCTCAACCTTTCCTTCTGCGGAACGGGGCGAATGAAGGATTTCAC<br>GAGGCAGTGGGTGAGATTATGTCCCTGTCAGCTGCCACTCCGAAACATCTGAAAAGCATCGGCC<br>TGTTGAGCCCAGACTTCCAAGAAGATAATGAGACCGAAATAAACTTCCTTCTGAAGCAAGCACT

	GACTATTGTAGGTACCTTGCCCTTTACATACATGCTGGAGAAGTGGAGGTGGATGGTATTTAAG<br>GGGGAGATACCGAAAGATCAATGGATGAAAAAGTGGTGGGAAATGAAAAGGGAGATCGTTGGCG<br>TAGTTGAACCAGTACCGCATGATGAGACGTACTGCGATCCGGCTAGTCTGTTCCATGTCTCTAA<br>TGATTACTCTTTCATCCGCTACTACACCCGCACGCTGTATCAATTCCAGTTCCAAGAAGCTCTC<br>TGTCAGGCTGCCAAGCACGAAGGACCGCTGCACAAATGCGACATTAGCAATTCTACAGAGGCGG<br>GTCAGAAGTTGTTCAATATGCTTAGACTGGGGAAGAGCGAACCGTGGACGCTCGCTTTGGAGAA<br>CGTTGTTGGAGCTAAGAATATGAACGTCAGGCCCTTGCTGAATTACTTTGAACCTCTGTTTACG<br>TGGTTGAAAGACCAAAATAAAAACTCCTTTGTTGGGTGGAGTACTGACTGGTCCCCCTATGCGG<br>ACCAAAGCATCAAAGTGAGGATAAGCCTAAAATCAGCTCTTGGAGATAAAGCATATGAATGGAA<br>CGACAATGAAATGTACCTGTTCCGATCATCTGTTGCATATGCTATGAGGCAGTACTTTTTAAAA<br>GTAAAAAATCAGATGATTCTTTTTGGGGAGGAGGATGTGCGAGTGGCTAATTTGAAACCAAGAA<br>TCTCCTTTAATTTCTTTGTCACTGCACCTAAAAATGTGTCTGATATCATTCCTAGAACTGAAGT<br>TGAAAAGGCCATCAGGATGTCCCGGAGCCGTATCAATGATGCTTTCCGTCTGAATGACAACAGC<br>CTAGAGTTTCTGGGGATACAGCCAACACTTGGACCTCCTAACCAGCCCCCTGTTTCC
354	CAATCTACCATCGAAGAGCAGGCCAAAACATTCCTCGACTTCTTTGATGCCCAGGCTGAAGACC<br>TTTTCTACCAATCAAGTCTGGCTAGCTGGGATTACAGTACAAGCATTACAGAGGGGAACGTGCA<br>AAACATGAATGACGCAGGGGACAAGTGGAGCGCATTCCTTAAGGAGCAAAGTACCCTTGCGCAA<br>ATGTATCCGCTGCAAGAGATTCAAAACCTGACGGTTAAGCTGCAACTTCAGGCCCTCCAGCAAA<br>ATGGATCCTCAGTCTTGTCAGAAGACAAAAGCAAGCGACTGAACACCATCCTTAACACCATGTC<br>AACCATATATTCAACAGGTAAAGTTTGCAATCCGGATAACCCCCAAGAATGTTTGCTTCTTGAA<br>CCCGGTCTCAACGAAATTATGGCCAACAGTCTTGATTACAACGAGCGATTGTGGGCATGGGAAA<br>GTTGGAGGAGTGAGGTAGGCAAACAGTTGAGACCTCTTTATGAAGAGTACGTTGTCCTTAAAAA<br>TGAAATGGCTCGCGCGAATCATTATGAAGACTATGGTGACTACTGGAGGGGGGATTATGAGGTG<br>AACGGGGTGGACGGATACGATTACTCTAGGGGCCAGCTGATAGAGGATGTCGAGCACACCTTTG<br>AGGAGATTAAGCCGTTGTACGAACATTTGCACGCCTATGTCAGGGCTAAGCTCATGAACGCTTA<br>TCCGAGTTATATCTCCCCGATAGGATGCTTGCCTGCTCACTTGTTGGGCGATATGTGGGGACGC<br>TTTTGGACCAACTTGTATTCCCTTACGGTACCGTTCGGCCAGAAACCAAATATCGACGTGACAG<br>ACGCAATGGTGGATCAAGCATGGGATGCGCAACGAATCTTCAAGGAGGCAGAAAAATTTTTCGT<br>TTCAGTTGGACTCCCAAACATGACGCAGGGTTTCTGGGAGAACTCAATGTTGACAGATCCAGGT<br>AATGTGCAGAAAGCGGTTTGCCTCCCTACTGCATGGGATCTTGGTAAAGGGGACTTCCGCATAC<br>TCATGTGTACGAAAGTAACTATGGACGACTTTCTTACTGCGCACCACGAGATGGGGCACATACA<br>ATACGATATGGCGTACGCAGCTCAACCTTTCCTTCTGCGGAACGGGGCGAATGAAGGATTTCAC<br>GAGGCAGTGGGTGAGATTATGTCCCTGTCAGCTGCCACTCCGAAACATCTGAAAAGCATCGGCC<br>TGTTGAGCCCAGACTTCCAAGAAGATAATGAGACCGAAATAAACTTCCTTCTGAAGCAAGCACT<br>GACTATTGTAGGTACCTTGCCCTTTACATACATGCTGGAGAAGTGGAGGTGGATGGTATTTAAG<br>GGGGAGATACCGAAAGATCAATGGATGAAAAAGTGGTGGGAAATGAAAAGGGAGATCGTTGGCG<br>TAGTTGAACCAGTACCGCATGATGAGACGTACTGCGATCCGGCTAGTCTGTTCCATGTCTCTAA<br>TGATTACTCTTTCATCCGCTACTACACCCGCACGCTGTATCAATTCCAGTTCCAAGAAGCTCTC<br>TGTCAGGCTGCCAAGCACGAAGGACCGCTGCACAAATGCGACATTAGCAATTCTACAGAGGCGG<br>GTCAGAAGTTGTTCAATATGCTTAGACTGGGGAAGAGCGAACCGTGGACGCTCGCTTTGGAGAA<br>CGTTGTTGGAGCTAAGAATATGAACGTCAGGCCCTTGCTGAATTACTTTGAACCTCTGTTTACG<br>TGGTTGAAAGACCAAAATAAAAACTCCTTTGTTGGGTGGAGTACTGACTGGTCCCCCTATGCGG<br>ACCAAAGCATCAAAGTGAGGATAAGCCTAAAATCAGCTCTTGGAGATAAAGCATATGAATGGAA<br>CGACAATGAAATGTACCTGTTCCGATCATCTGTTGCATATGCTATGAGGCAGTACTTTTTAAAA<br>GTAAAAAATCAGATGATTCTTTTTGGGGAGGAGGATGTGCGAGTGGCTAATTTGAAACCAAGAA<br>TCTCCTTTAATTTCTTTGTCACTGCACCTAAAAATGTGTCTGATATCATTCCTAGAACTGAAGT

	TGAAAAGGCCATCAGGATGTCCCGGAGCCGTATCAATGATGCTTTCCGTCTGAATGACAACAGC<br>CTAGAGTTTCTGGGGATACAGCCAACACTTGGACCTCCTAACCAGCCCCCTGTTTCC
355	CAACCAACCATCGAAGAGCAGGCCAAAACATTCCTCGACAAGTTTAATCACGAGGCTGAAGACC<br>TTTTCTACTTGTCAAGTCTGGCTAGCTGGAATTACAATACAAACATTACAGAGGAGAACGTACA<br>AAACATGAATAACGCAGGGGACAAGTGGAGCGCATTCCTTAAGGAACAAAGTACCACTGCGCAA<br>ATGTATCCGCTGCAAGAGATTCAACAGCTGACGGTTAAGCTGCAACTTCAGGCCCTCCAACAAA<br>ATGGATCCTCAGTCTTGTCAGAAGACAAAAGCAAGCGACTGAACACCATCCTTAACACCATGTC<br>AACCATATATTCAACAGGTAAAGTTTGCAATCCGGATAACCCCCAAGAATGTTTGCTTCTTGAA<br>CCCGGTCTCAACGAAATTATGGCCAACAGTCTTGATTACAACGAGCGATTGTGGGCATGGGAAA<br>GTTGGAGGAGTGAGGTAGGCAAACAGTTGAGACCTCTTTATGAAGAGTACGTTGTCCTTAAAAA<br>TGAAATGGCTCGCGCGAATCATTATGAAGACTATGGTGACTACTGGAGGGGGGATTATGAGGTG<br>AACGGGGTGGACGGATACGATTACTCTAGGGGCCAGCTGATAGAGGATGTCGAGCACACCTTTG<br>AGGAGATTAAGCCGTTGTACGAACATTTGCACGCCTATGTCAGGGCTAAGCTCATGAACGCTTA<br>TCCGAGTTATATCTCCCCGATAGGATGCTTGCCTGCTCACTTGTTGGGCGATATGTGGGGACGC<br>TTTTGGACCAACTTGTATTCCCTTACGGTACCGTTCGGCCAGAAACCAAATATCGACGTGACAG<br>ACGCAATGGTGGATCAAGCATGGGATGCGCAACGAATCTTCAAGGAGGCAGAAAAATTTTTCGT<br>TTCAGTTGGACTCCCAAACATGACGCAGGGTTTCTGGGAGAACTCAATGTTGACAGATCCAGGT<br>AATGTGCAGAAAGCGGTTTGCCTCCCTACTGCATGGGATCTTGGTAAAGGGGACTTCCGCATAC<br>TCATGTGTACGAAAGTAACTATGGACGACTTTCTTACTGCGCACCACGAGATGGGGCACATACA<br>ATACGATATGGCGTACGCAGCTCAACCTTTCCTTCTGCGGAACGGGGCGAATGAAGGATTTCAC<br>GAGGCAGTGGGTGAGATTATGTCCCTGTCAGCTGCCACTCCGAAACATCTGAAAAGCATCGGCC<br>TGTTGAGCCCAGACTTCCAAGAAGATAATGAGACCGAAATAAACTTCCTTCTGAAGCAAGCACT<br>GACTATTGTAGGTACCTTGCCCTTTACATACATGCTGGAGAAGTGGAGGTGGATGGTATTTAAG<br>GGGGAGATACCGAAAGATCAATGGATGAAAAAGTGGTGGGAAATGAAAAGGGAGATCGTTGGCG<br>TAGTTGAACCAGTACCGCATGATGAGACGTACTGCGATCCGGCTAGTCTGTTCCATGTCTCTAA<br>TGATTACTCTTTCATCCGCTACTACACCCGCACGCTGTATCAATTCCAGTTCCAAGAAGCTCTC<br>TGTCAGGCTGCCAAGCACGAAGGACCGCTGCACAAATGCGACATTAGCAATTCTACAGAGGCGG<br>GTCAGAAGTTGTTCAATATGCTTAGACTGGGGAAGAGCGAACCGTGGACGCTCGCTTTGGAGAA<br>CGTTGTTGGAGCTAAGAATATGAACGTCAGGCCCTTGCTGAATTACTTTGAACCTCTGTTTACG<br>TGGTTGAAAGACCAAAATAAAAACTCCTTTGTTGGGTGGAGTACTGACTGGTCCCCCTATGCGG<br>ACCAAAGCATCAAAGTGAGGATAAGCCTAAAATCAGCTCTTGGAGATAAAGCATATGAATGGAA<br>CGACAATGAAATGTACCTGTTCCGATCATCTGTTGCATATGCTATGAGGCAGTACTTTTTAAAA<br>GTAAAAAATCAGATGATTCTTTTTGGGGAGGAGGATGTGCGAGTGGCTAATTTGAAACCAAGAA<br>TCTCCTTTAATTTCTTTGTCACTGCACCTAAAAATGTGTCTGATATCATTCCTAGAACTGAAGT<br>TGAAAAGGCCATCAGGATGTCCCGGAGCCGTATCAATGATGCTTTCCGTCTGAATGACAACAGC<br>CTAGAGTTTCTGGGGATACAGCCAACACTTGGACCTCCTAACCAGCCCCCTGTTTCC
373	CGATCTACCATCGAAGAGCAGGCCAAAACATTCCTCGACTTCTTTGATAGCCAGGCTGAAGACC<br>TTTTCTACCAATCAAGTCTGGCAAGCTGGAATTACAACACAAACATTACAGAGGAGAACGTACA<br>AAACATGAATAACGCAGGGGACAAGCGGAGCGCATTCCTTAAGGAACGAAGTACCCTTGCGCAG<br>ATGTATCCGCTGCAAGAGATTCAAAACCTGACGGTTAAGCTGCAACTTCAGGCCCTCCAACAAA<br>ATGGATCCTCAGTCTTGTCAGAAGACAAAAGCAAGCGACTGAACACCATCCTTAACACCATGTC<br>AACCATATATTCAACAGGTAAAGTTTGCAATCCGGATAACCCCCAAGAATGTTTGCTTCTTGAA<br>CCCGGTCTCAACGAAATTATGGCCAACAGTCTTGATTACAACGAGCGATTGTGGGCATGGGAAA<br>GTTGGAGGAGTGAGGTAGGCAAACAGTTGAGACCTCTTTATGAAGAGTACGTTGTCCTTAAAAA<br>TGAAATGGCTCGCGCGAATCATTATGAAGACTATGGTGACTACTGGAGGGGGGATTATGAGGTG<br>AACGGGGTGGACGGATACGATTACTCTAGGGGCCAGCTGATAGAGGATGTCGAGCACACCTTTG<br>AGGAGATTAAGCCGTTGTACGAACATTTGCACGCCTATGTCAGGGCTAAGCTCATGAACGCTTA

	TCCGAGTTATATCTCCCCGATAGGATGCTTGCCTGCTCACTTGTTGGGCGATATGTGGGGACGC<br>TTTTGGACCAACTTGTATTCCCTTACGGTACCGTTCGGCCAGAAACCAAATATCGACGTGACAG<br>ACGCAATGGTGGATCAAGCATGGGATGCGCAACGAATCTTCAAGGAGGCAGAAAAATTTTTCGT<br>TTCAGTTGGACTCCCAAACATGACGCAGGGTTTCTGGGAGAACTCAATGTTGACAGATCCAGGT<br>AATGTGCAGAAAGCGGTTTGCCTCCCTACTGCATGGGATCTTGGTAAAGGGGACTTCCGCATAC<br>TCATGTGTACGAAAGTAACTATGGACGACTTTCTTACTGCGCACCACGAGATGGGGCACATACA<br>ATACGATATGGCGTACGCAGCTCAACCTTTCCTTCTGCGGAACGGGGCGAATGAAGGATTTCAC<br>GAGGCAGTGGGTGAGATTATGTCCCTGTCAGCTGCCACTCCGAAACATCTGAAAAGCATCGGCC<br>TGTTGAGCCCAGACTTCCAAGAAGATAATGAGACCGAAATAAACTTCCTTCTGAAGCAAGCACT<br>GACTATTGTAGGTACCTTGCCCTTTACATACATGCTGGAGAAGTGGAGGTGGATGGTATTTAAG<br>GGGGAGATACCGAAAGATCAATGGATGAAAAAGTGGTGGGAAATGAAAAGGGAGATCGTTGGCG<br>TAGTTGAACCAGTACCGCATGATGAGACGTACTGCGATCCGGCTAGTCTGTTCCATGTCTCTAA<br>TGATTACTCTTTCATCCGCTACTACACCCGCACGCTGTATCAATTCCAGTTCCAAGAAGCTCTC<br>TGTCAGGCTGCCAAGCACGAAGGACCGCTGCACAAATGCGACATTAGCAATTCTACAGAGGCGG<br>GTCAGAAGTTGTTCAATATGCTTAGACTGGGGAAGAGCGAACCGTGGACGCTCGCTTTGGAGAA<br>CGTTGTTGGAGCTAAGAATATGAACGTCAGGCCCTTGCTGAATTACTTTGAACCTCTGTTTACG<br>TGGTTGAAAGACCAAAATAAAAACTCCTTTGTTGGGTGGAGTACTGACTGGTCCCCCTATGCGG<br>ACCAAAGCATCAAAGTGAGGATAAGCCTAAAATCAGCTCTTGGAGATAAAGCATATGAATGGAA<br>CGACAATGAAATGTACCTGTTCCGATCATCTGTTGCATATGCTATGAGGCAGTACTTTTTAAAA<br>GTAAAAAATCAGATGATTCTTTTTGGGGAGGAGGATGTGCGAGTGGCTAATTTGAAACCAAGAA<br>TCTCCTTTAATTTCTTTGTCACTGCACCTAAAAATGTGTCTGATATCATTCCTAGAACTGAAGT<br>TGAAAAGGCCATCAGGATGTCCCGGAGCCGTATCAATGATGCTTTCCGTCTGAATGACAACAGC<br>CTAGAGTTTCTGGGGATACAGCCAACACTTGGACCTCCTAACCAGCCCCCTGTTTCC
375	CAACCTACCATCGAAGAGCAGGCCAAAACATTCCTCGACAAGTTTAGTGTCGAGGCTGAAGACC<br>TTCTCTACCAATCAAGTCTGGCTAGCTGGGATTACAACACAAACATTACAGAGGAGAACGTACA<br>AAACATGAATAACGCAGGGGACAAATGGAGCGCATTCCTCAAGGAACAAAGTACCCTTGCGCAA<br>ATGTATCCGCTGCAAGAGATTCAAAACCTGACGGTTAAGCTGCAACTTCAGGCCCCCCAACAAA<br>ATGGATCCTCAGTCTTGTCAGAAGACAAAAGCAAGCGACTGAACACCATCCTTAACACCATGTC<br>AACCATATATTCAACAGGTAAAGTTTGCAATCCGGATAACCCCCAAGAATGTTTGCTTCTTGAA<br>CCCGGTCTCAACGAAATTATGGCCAACAGTCTTGATTACAACGAGCGATTGTGGGCATGGGAAA<br>GTTGGAGGAGTGAGGTAGGCAAACAGTTGAGACCTCTTTATGAAGAGTACGTTGTCCTTAAAAA<br>TGAAATGGCTCGCGCGAATCATTATGAAGACTATGGTGACTACTGGAGGGGGGATTATGAGGTG<br>AACGGGGTGGACGGATACGATTACTCTAGGGGCCAGCTGATAGAGGATGTCGAGCACACCTTTG<br>AGGAGATTAAGCCGTTGTACGAACATTTGCACGCCTATGTCAGGGCTAAGCTCATGAACGCTTA<br>TCCGAGTTATATCTCCCCGATAGGATGCTTGCCTGCTCACTTGTTGGGCGATATGTGGGGACGC<br>TTTTGGACCAACTTGTATTCCCTTACGGTACCGTTCGGCCAGAAACCAAATATCGACGTGACAG<br>ACGCAATGGTGGATCAAGCATGGGATGCGCAACGAATCTTCAAGGAGGCAGAAAAATTTTTCGT<br>TTCAGTTGGACTCCCAAACATGACGCAGGGTTTCTGGGAGAACTCAATGTTGACAGATCCAGGT<br>AATGTGCAGAAAGCGGTTTGCCTCCCTACTGCATGGGATCTTGGTAAAGGGGACTTCCGCATAC<br>TCATGTGTACGAAAGTAACTATGGACGACTTTCTTACTGCGCACCACGAGATGGGGCACATACA<br>ATACGATATGGCGTACGCAGCTCAACCTTTCCTTCTGCGGAACGGGGCGAATGAAGGATTTCAC<br>GAGGCAGTGGGTGAGATTATGTCCCTGTCAGCTGCCACTCCGAAACATCTGAAAAGCATCGGCC<br>TGTTGAGCCCAGACTTCCAAGAAGATAATGAGACCGAAATAAACTTCCTTCTGAAGCAAGCACT<br>GACTATTGTAGGTACCTTGCCCTTTACATACATGCTGGAGAAGTGGAGGTGGATGGTATTTAAG<br>GGGGAGATACCGAAAGATCAATGGATGAAAAAGTGGTGGGAAATGAAAAGGGAGATCGTTGGCG<br>TAGTTGAACCAGTACCGCATGATGAGACGTACTGCGATCCGGCTAGTCTGTTCCATGTCTCTAA<br>TGATTACTCTTTCATCCGCTACTACACCCGCACGCTGTATCAATTCCAGTTCCAAGAAGCTCTC

	TGTCAGGCTGCCAAGCACGAAGGACCGCTGCACAAATGCGACATTAGCAATTCTACAGAGGCGG<br>GTCAGAAGTTGTTCAATATGCTTAGACTGGGGAAGAGCGAACCGTGGACGCTCGCTTTGGAGAA<br>CGTTGTTGGAGCTAAGAATATGAACGTCAGGCCCTTGCTGAATTACTTTGAACCTCTGTTTACG<br>TGGTTGAAAGACCAAAATAAAAACTCCTTTGTTGGGTGGAGTACTGACTGGTCCCCCTATGCGG<br>ACCAAAGCATCAAAGTGAGGATAAGCCTAAAATCAGCTCTTGGAGATAAAGCATATGAATGGAA<br>CGACAATGAAATGTACCTGTTCCGATCATCTGTTGCATATGCTATGAGGCAGTACTTTTTAAAA<br>GTAAAAAATCAGATGATTCTTTTTGGGGAGGAGGATGTGCGAGTGGCTAATTTGAAACCAAGAA<br>TCTCCTTTAATTTCTTTGTCACTGCACCTAAAAATGTGTCTGATATCATTCCTAGAACTGAAGT<br>TGAAAAGGCCATCAGGATGTCCCGGAGCCGTATCAATGATGCTTTCCGTCTGAATGACAACAGC<br>CTAGAGTTTCTGGGGATACAGCCAACACTTGGACCTCCTAACCAGCCCCCTGTTTCC
